# Supplementary material for: Control cell migration by engineering integrin ligand assembly
Source: Nat Commun. 2022 Aug 25;13:5002. doi: 10.1038/s41467-022-32686-2 (PMC9411606; doi:10.1038/s41467-022-32686-2)
Supplement: Supplementary file 1 — Supplementary Information [file 41467_2022_32686_MOESM1_ESM.pdf]

Supplementary Materials for

Control Cell Migration by Engineering Integrin Ligand Assembly

Xunwu Hu, Sona Rani Roy, Chengzhi Jin, Guanying Li, Qizheng Zhang, Natsuko Asano, Shunsuke Asahina, Tomoko Kajiwara, Chenjie Xu, Kazuhiro Aoki, Atsushi Takahara, Ye Zhang*

Correspondence to: zhangye@sslab.org.cn

This PDF file includes:

Supplementary Note
Supplementary Figures 1 to 49

Supplementary Note

Molecular dynamics simulation and polymorph prediction (six-step process)

1. Import the crystal structure of FFF to Materials Studio and draw FFFIKLLI based on the conformation of FFF. The geometrical energy minimization scans were performed using the Forcite module of Materials Studio. The molecule in Supplementary Figure 11a was found to have the lowest energy conformation. The force field used was Dreiding 2.21 with Gasteiger charges as implemented in the Materials Studio packages.
2. Import the FFF unit cell obtained via single crystal XRD measurements as described in the protocol to Materials Studio. Replacing FFF by optimized FFFIKLLI to 1:1, 2:1 and 4:1 ratio, respectively. The initial placement of FFFIKLLI was determined by π-interactions between adjacent aromatic rings along the c-axis. The geometrical energy minimization scans were performed using the Forcite module. The optimized gas phase conformations as presented in Supplementary Figure 11b were used as the starting points for crystal structure prediction using the Materials Studio Polymorph Predictor (PP).
3. The PP was set to its default fine setting (this sets the simulated annealing algorithm to a temperature range of 300-100000.0 K with a heating factor of 0.025, requiring 12 consecutive steps to be accepted before cooling and a maximum of 7000 steps) with the force field Dreiding 2.21 with Gasteiger charges. The 10 most common space groups found in organic crystals registered in the CSD were selected, including $P2_1/c$, $P1$, $P2_12_12_1$, $P2_1$, $C2/c$, *Pbca*, $Pna2_1$, *Pbcn*, *Cc*, and *C2*. Clustering of the predicted polymorphs was done using the polymorph clustering routine in Materials Studio. After the final clustering, hydrogen bonding analysis (as implemented in the Materials Studio packages) was performed on the calculated crystal structures (Supplementary Figure 12). According to the FTIR spectra, self-assembled FFF presents NH-O hydrogen bonding, self-assembled FFFIKLLI presents NH-N hydrogen bonding, co-assembled FFF and FFFIKLLI present both NH-O and NH-N hydrogen bonding. The reported FFF unit cell shows both intermolecular and intramolecular NH-O hydrogen bonding which matches to the FTIR results. Based on the summarized hydrogen bonding analysis of the calculated crystal structures (Supplementary Table 1), we highlighted the structures that match to the FTIR results in black frames.
4. The TEM images and SEM images of self-assembled FFF, self-assembled FFFIKLLI, co-assembled FFF with FFFIKLLI demonstrated that the assembled nanofilaments all shared similar morphologies that were barely influenced by the variation of components' proportion. Taking advantage of unified space group symmetry to reduce the number of variables in searches of molecular packing modes in nanofilaments assembled by FFF and FFFIKLLI at various proportions, we selected the clustering results of *Pbcn* space group (Supplementary Figure 13) which generated matching structures at different proportions to ease the comparisons in regard of ligand (IKLLI) distribution density.
5. After extending the structure along the unit axes, the surface that exposes most integrin ligand IKLLI was presented in a defined square area (10 x10 nm^2) within the dimension range of nanofilaments (Supplementary Figure 14). The ligand density on the surface of nanofilaments formed by co-assembly of FFFIKLLI and FFF at 1:44, 1: 89, and 1:249 ratio was roughly estimated statistically based on the crystal structure of FFF unit cell by replacing one FFF with FFFIKLLI on the filament that composed of 45, 90, and 250 FFF, respectively. The alteration of packing dimension induced by the insertion of FFFIKLLI was ignored since the majority of the packing molecules are FFF. Because the C-terminus of FFF that can be covalently linked with IKLLI only exposes toward the zx plain, we took the area size of this plain for surface estimation. The detailed calculation results are presented in Supplementary Figure 15.
6. The exposed ligand IKLLI was identified as effective ligand, and the distance between the effective ligands was measured using the distance measurement functions of Materials Studio by

calculating the distance between the C-terminals of the effective ligands (Supplementary Figure 14).

Peptides

FFF

Purity: 99.51%

Molecular weight: 459.57. ESI-MS (m/z) $[M+H]^+$ = 460.15 (Supplementary Figure 37)

^{1}H NMR (500 MHz, DMSO-d_6) δ 12.83 (s, 1H), 8.74 (d, J = 7.5 Hz, 1H), 8.56 (d, J = 7.8 Hz, 1H), 7.99 (s, 2H), 7.30 – 7.17 (m, 15H), 4.64 (td, J = 8.7, 4.6 Hz, 1H), 4.50 (td, J = 8.3, 5.3 Hz, 1H), 3.97 (s, 1H), 3.13 – 3.00 (m, 3H), 2.94 (dd, J = 14.0, 8.8 Hz, 1H), 2.86 (dd, J = 14.3, 8.5 Hz, 1H), 2.79 (dd, J = 14.0, 9.3 Hz, 1H). (Supplementary Figure 38)

FFIKLLI

Purity: 98.94%

Molecular weight: 893.17. ESI-MS (m/z) $[M-H]^-$ = 891.56 (Supplementary Figure 39)

FFFIKLLI

Purity: 98.32%

Molecular weight: 1040.44. ESI-MS (m/z) $[M+H]^+$ = 1040.60 (Supplementary Figure 40)

^{1}H NMR (500 MHz, DMSO-d_6) δ 12.61 (1H, s), 8.72-8.65 (1H, m), 8.40 (1H, d, J = 8.0 Hz), 8.07 (1H, d, J = 8.8 Hz), 8.05 (1H, d, J = 8.2 Hz), 8.01-7.94 (4H, m), 7.74 (1H, d, J = 8.4 Hz), 7.66 (2H, s), 7.31-7.13 (15H, m), 4.72-4.66 (1H, m), 4.64-4.58 (1H, m), 4.40-4.22 (4H, m), 4.16 (1H, dd, J = 8.3, 5.9 Hz), 3.97 (1H, s), 3.06-2.98 (3H, m), 2.87-2.77 (3H, m), 2.77-2.70 (2H, m), 1.81-1.69 (2H, m), 1.66-1.34 (12H, m), 1.34-1.24 (2H, m), 1.21-1.04 (2H, m), 0.91-0.75 (24H, m) ppm. (Supplementary Figure 41)

FFFKLIIL

Purity: 98.72%

Molecular weight: 1040.34. ESI-MS (m/z) $[M+H]^+$ = 1040.75 (Supplementary Figure 42)

^{1}H NMR (500 MHz, DMSO-d_6) δ 12.48 (s, 1H), 8.68 (d, J = 8.2 Hz, 1H), 8.40 (d, J = 7.9 Hz, 1H), 8.21 (d, J = 8.0 Hz, 1H), 8.12 (d, J = 7.9 Hz, 1H), 8.05 – 7.97 (m, 3H), 7.93 (d, J = 8.8 Hz, 1H), 7.81 – 7.73 (m, 3H), 7.29 – 7.13 (m, 15H), 4.66 – 4.58 (m, 2H), 4.37 (dd, J = 15.1, 7.7 Hz, 1H), 4.31 (dd, J = 13.8, 7.9 Hz, 1H), 4.25 – 4.16 (m, 3H), 3.97 (s, 1H), 3.08 – 2.99 (m, 3H), 2.88 – 2.70 (m, 5H), 1.75 – 1.36 (m, 12H), 1.35 – 1.25 (m, 2H), 1.05 (dt, J = 13.6, 7.2 Hz, 2H), 0.91 – 0.74 (m, 24H). (Supplementary Figure 43)

FFFGRGDSP

Purity: 98.54%.

Molecular weight: 1029.20. ESI-MS (m/z) $[M+H]^+$ = 1029.40 (Supplementary Figure 44)

^{1}H NMR (500 MHz, DMSO-d_6) δ 9.43 (s, 1H), 8.65 (s, 1H), 8.60 (s, 1H), 8.57 – 8.43 (m, 3H), 8.24 (t, J = 5.4 Hz, 1H), 8.21 – 8.09 (m, 1H), 7.42 (s, 1H), 7.32 – 7.12 (m, 17H), 4.62 – 4.52 (m, 3H), 4.44 – 4.27 (m, 3H), 4.22 (dd, J = 8.7, 4.1 Hz, 1H), 3.86 – 3.72 (m, 4H), 3.66 – 3.51 (m, 3H), 3.50 – 3.32 (m, 2H), 3.20 – 3.10 (m, 1H), 3.09 – 2.94 (m, 5H), 2.86 (dd, J = 13.9, 9.6 Hz, 1H), 2.82 – 2.70 (m, 2H), 2.57 (dd, J = 16.5, 5.0 Hz, 1H), 2.15 – 2.03 (m, 1H), 1.89 – 1.77 (m, 2H), 1.75 – 1.40 (m, 4H). (Supplementary Figure 45)

FFFLRGDN

Purity: 98.73%.

Molecular weight: 1015.21. ESI-MS (m/z) $[M-H]^-$ = 1013.60 (Supplementary Figure 46)

^{1}H NMR (500 MHz, DMSO-d_6) δ 12.46 (s, 2H), 8.69 (d, J = 8.3 Hz, 1H), 8.41 (d, J = 8.1 Hz, 1H), 8.24 (d, J = 8.2 Hz, 1H), 8.21 (d, J = 7.8 Hz, 1Hz), 8.17 – 8.14 (m, 1H), 8.11 (d, J = 8.0 Hz, 1H), 8.02 (d, J = 7.6 Hz, 1H), 7.98 (s, 2H), 7.59 (s, 1H), 7.40 (s, 1H), 7.31 – 7.17 (m, 15H), 6.93 (s, 1H), 4.65 – 4.59 (m, 1H), 4.51 – 4.46 (m, 1H), 4.44 – 4.28 (m, 2H), 4.13 (s, 2H), 3.97 (s, 1H), 3.82 – 3.67 (m, 2H), 3.62 (d, J = 3.3

Hz, 1H), 3.59 (d, J = 3.3 Hz, 1H), 3.55 (s, 1H), 3.53 (s, 1H), 3.48 – 3.42 (m, 2H), 3.05 – 2.99 (m, 2H), 2.88 – 2.75 (m, 3H), 2.68 (dd, J = 16.7, 5.0 Hz, 1H), 1.75 – 1.60 (m, 2H), 1.58 – 1.43 (m, 5H), 0.89 (d, J = 6.6 Hz, 3H), 0.86 (d, J = 6.5 Hz, 3H). (Supplementary Figure 47)

FFFIKVAV
Purity: 98.70%.
Molecular weight: 970.21. ESI-MS (m/z) $[M+H]^+$ = 970.55 (Supplementary Figure 48)
^{1}H NMR (500 MHz, DMSO-d_6) δ 12.64 (s, 1H), 8.70 (d, J = 8.3 Hz, 1H), 8.41 (d, J = 8.1 Hz, 1H), 8.13 – 8.07 (m, 2H), 8.03 (d, J = 7.4 Hz, 1H), 7.99 (s, 2H), 7.88 (d, J = 8.6 Hz, 1H), 7.77 (d, J = 8.9 Hz, 1H), 7.72 (s, 2H), 7.31 – 7.15 (m, 15H), 4.69 (td, J = 8.8, 4.3 Hz, 1H), 4.61 (td, J = 8.6, 4.4 Hz, 1H), 4.41 – 4.36 (m, 1H), 4.36 – 4.30 (m, 1H), 4.28 – 4.23 (m, 1H), 4.19 (dd, J = 8.8, 6.5 Hz, 1H), 4.14 (dd, J = 8.6, 5.6 Hz, 1H), 3.97 (s, 1H), 3.06 – 2.99 (m, 3H), 2.87 – 2.71 (m, 5H), 2.05 (dq, J = 13.5, 6.8 Hz, 1H), 1.96 (dq, J = 13.5, 6.7 Hz, 1H), 1.78 – 1.70 (m, 1H), 1.70 – 1.62 (m, 1H), 1.58 – 1.40 (m, 4H), 1.37 – 1.26 (m, 2H), 1.19 (d, J = 7.0 Hz, 3H), 1.13 – 1.06 (m, 1H), 0.89 – 0.78 (m, 18H). (Supplementary Figure 49)

Supplementary Figures:

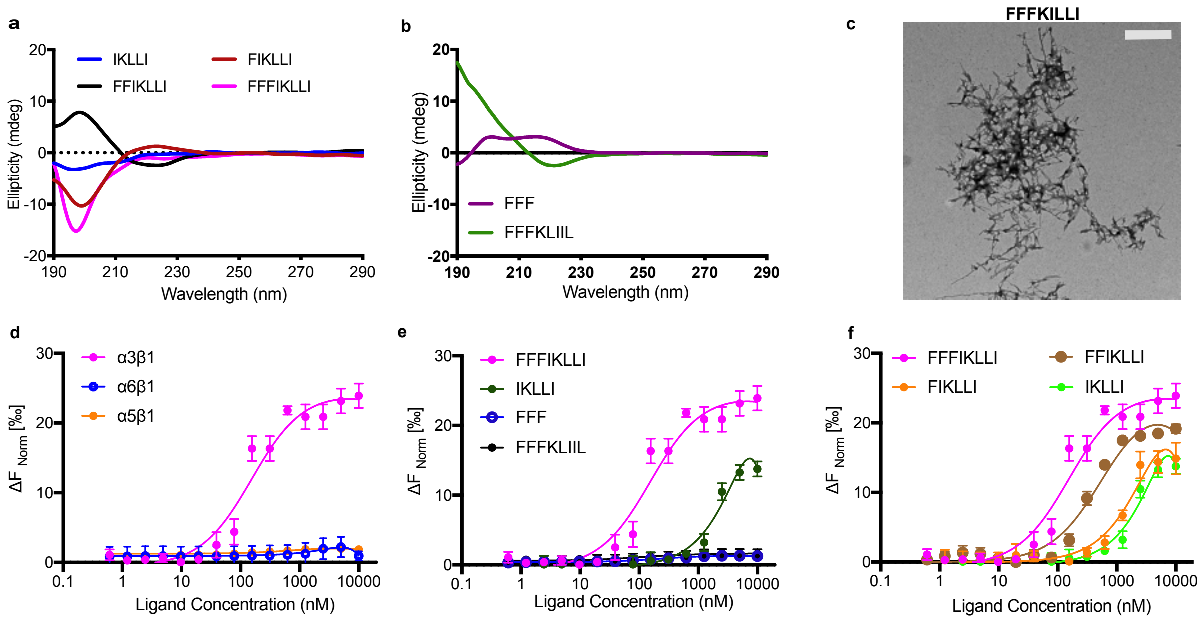

Supplementary Figure 1. Characterization of engineered integrin ligand in solution.

(**a**) Circular dichroism (CD) spectra of IKLLI, FIKLLI, FFIKLLI, FFFIKLLI at 100 μM concentration. (**b**) CD spectra of FFFKLIIL and FFF at 100 μM concentration. Three independent experiments were performed. (**c**) Representative TEM images of self-assembly of FFFKLIIL (scrambled peptide sequence was synthesized as negative control) in water at 100 μM concentration. Scale bar represents 500 nm. Three independent experiments were performed. (**d**) The binding affinity of FFFIKLLI, IKLLI, FFF and FFFKLIIL to integrin α3β1 measured by MicroScale Thermophoresis. Protein concentration, 2nM. n = 3 biological replicates. (**e**) The binding affinity of FFFIKLLI, FFIKLLI, FIKLLI and KLIIL to integrin α3β1 measured by MicroScale Thermophoresis. Protein concentration, 2nM. n = 3 biological replicates. (**f**) The binding affinity of FFFIKLLI to integrin α3β1, α5β1and α6β1 measured by MicroScale Thermophoresis. Protein concentration, 2 nM. n = 3 biological replicates. Data are presented as mean ± s.d.. Source numerical data are available in source data.

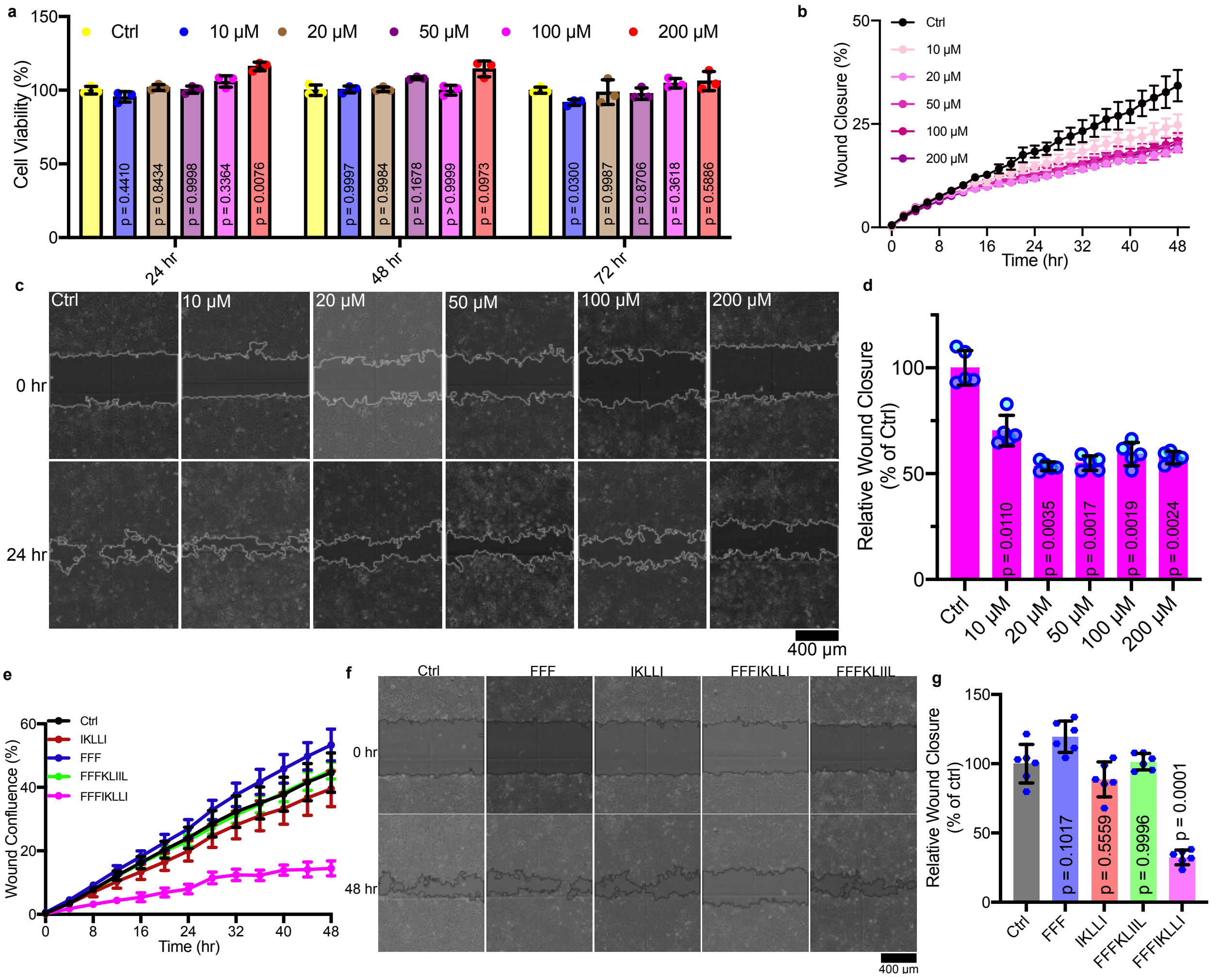

Supplementary Figure 2. Evaluation of engineered ligand on HuH-7 cells.

(**a**) HuH-7 cell viability using MTT assay. Cells were incubated with 10, 20, 50, 100, 200 μM of FFFIKLLI for 24, 48, or 72 hr. Kruskal-Wallis with Dunn's multiple comparisons test was used for analysis of the data. n = 3 biological replicates. Data are presented as mean ± s.d.. (**b-d**) Wound closure rate and representative images of HuH-7 cells with or without the treatment of FFFIKLLI at 10, 20, 50, 100, and 200 μM. Scale bar represents 400 μm. Kruskal-Wallis with Dunn's multiple comparisons test was used for analysis of the data. n = 5 biological replicates. Data are presented as mean ± s.d.. (**e-g**) Wound closure rate and representative images of HuH-7 cells with or without the treatment of FFF, IKLLI, FFFIKLLI, and FFFKLIIL at 100 μM. Scale bar represents 400 μm. Kruskal-Wallis with Dunn's multiple comparisons test was used for analysis of the data. n = 6 biological replicates. Data are presented as mean ± s.d.. Source numerical data are available in source data.

a

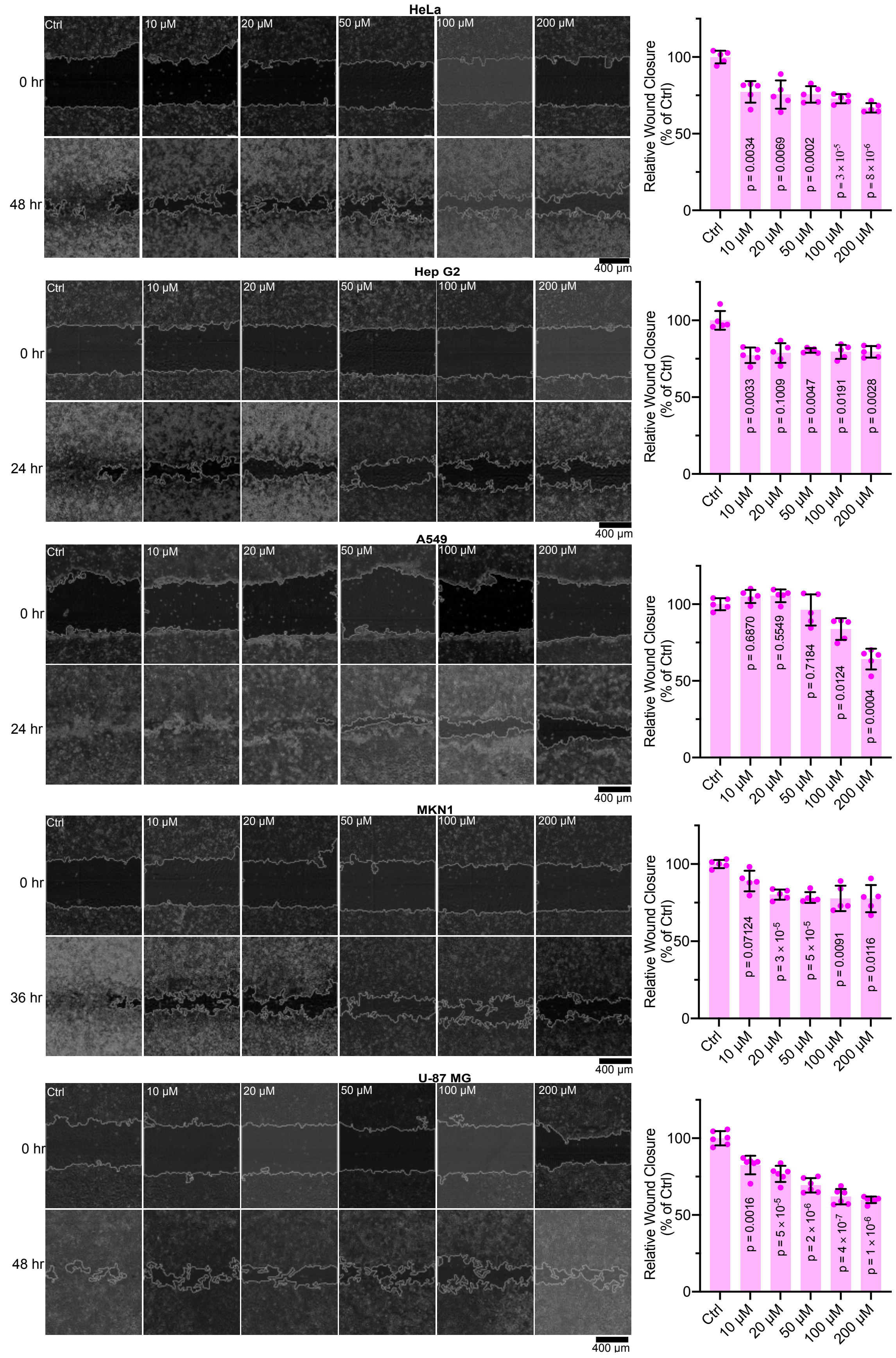
HeLa
Ctrl
10 μM
20 μM
50 μM
100 μM
200 μM
0 hr
48 hr
400 μm
Relative Wound Closure (% of Ctrl)
p = 0.0034
p = 0.0069
p = 0.0002
$p = 3 \times 10^{-5}$
$p = 8 \times 10^{-6}$
Hep G2
0 hr
24 hr
p = 0.0033
p = 0.1009
p = 0.0047
p = 0.0191
p = 0.0028
A549
0 hr
24 hr
p = 0.6870
p = 0.5549
p = 0.7184
p = 0.0124
p = 0.0004
MKN1
0 hr
36 hr
p = 0.07124
$p = 3 \times 10^{-5}$
$p = 5 \times 10^{-5}$
p = 0.0091
p = 0.0116
U-87 MG
0 hr
48 hr
p = 0.0016
$p = 5 \times 10^{-5}$
$p = 2 \times 10^{-6}$
$p = 4 \times 10^{-7}$
$p = 1 \times 10^{-6}$

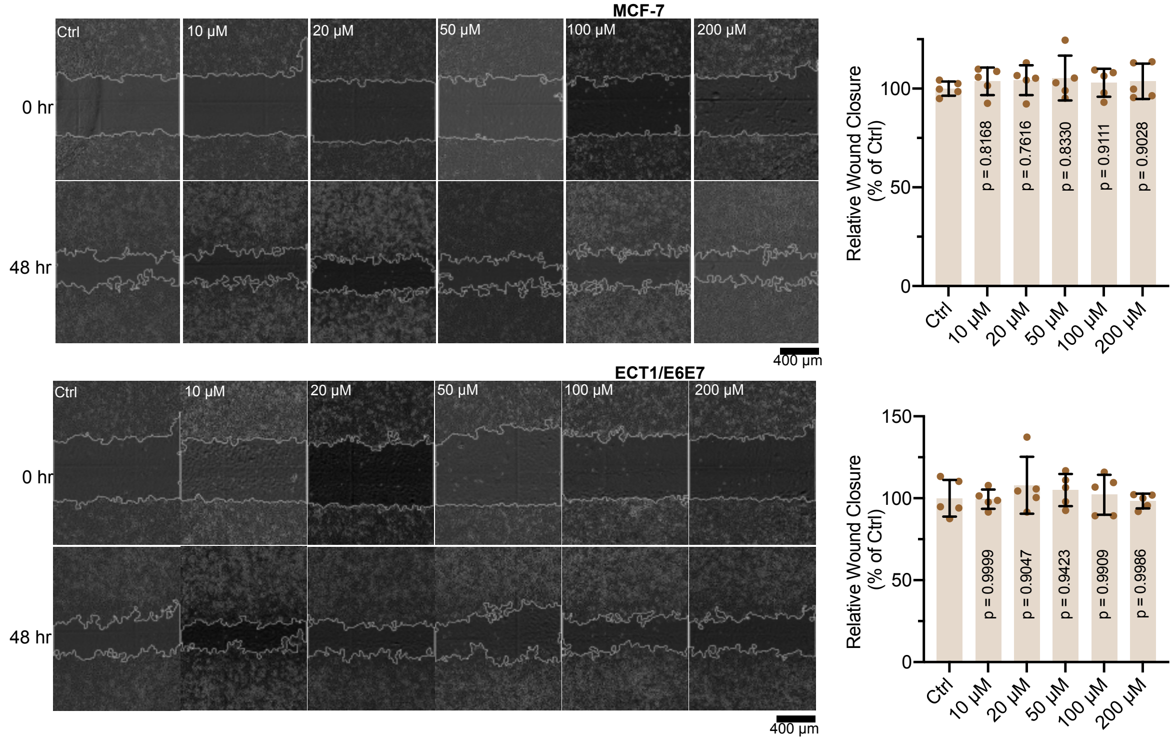

Supplementary Figure 3. Evaluation of FFFIKLLI on multiple cell lines.

Wound closure rate and the representative images of peptide FFFIKLLI treatment on HeLa, Hep G2, A549, MKN1, U-87 MG MCF-7 and Ect1/E6E7 cell lines. Scale bar represents 400 µm. Kruskal-Wallis with Dunn's multiple comparisons test was used for analysis of the data. At least five biological replicates were collected. Data are presented as mean ± s.d.. Source numerical data are available in source data.

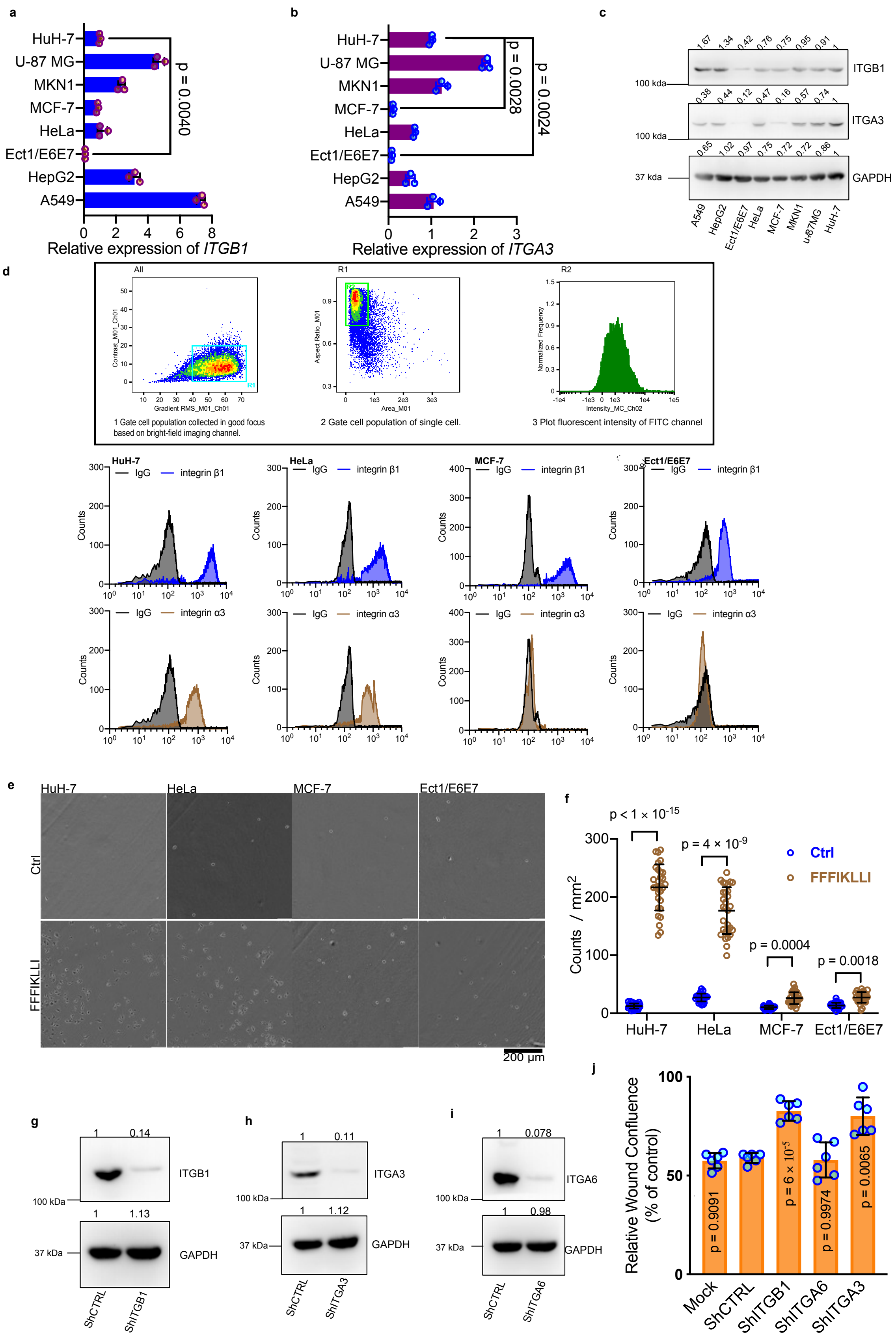

a
HuH-7
U-87 MG
MKN1
MCF-7
HeLa
Ect1/E6E7
HepG2
A549
p = 0.0040
Relative expression of ITGB1
b
p = 0.0028
p = 0.0024
Relative expression of ITGA3
c
ITGB1
ITGA3
GAPDH
100 kda
37 kda
A549
HepG2
Ect1/E6E7
HeLa
MCF-7
MKN1
u-87MG
HuH-7
d
1 Gate cell population collected in good focus based on bright-field imaging channel.
2 Gate cell population of single cell.
3 Plot fluorescent intensity of FITC channel
IgG
integrin β1
integrin α3
Counts
e
Ctrl
FFFIKLLI
200 μm
f
p < 1 × 10-15
p = 4 × 10-9
p = 0.0004
p = 0.0018
Counts / mm2
g
h
i
ShCTRL
ShITGB1
ShITGA3
ShITGA6
ITGA6
100 kDa
37 kDa
j
Relative Wound Confluence (% of control)
Mock
p = 0.9091
p = 6 × 10-5
p = 0.9974
p = 0.0065

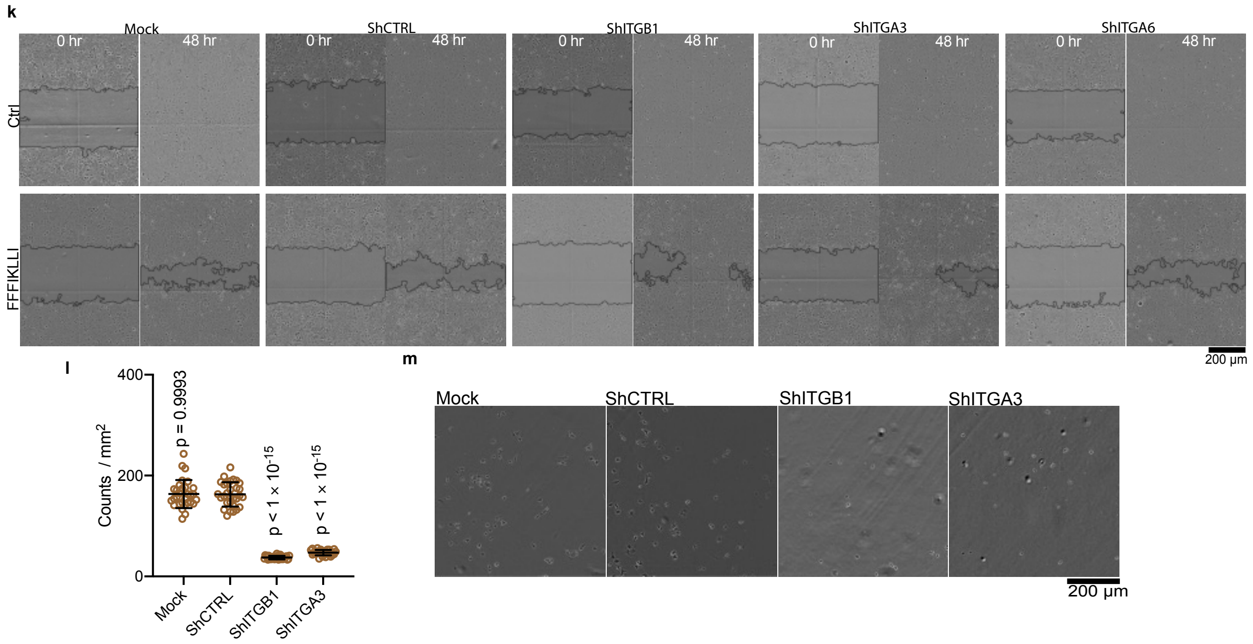

Supplementary Figure 4. FFFIKLLI selectively targets integrin β1.

(**a-b**) mRNA expression of integrin β1 and integrin α3 in different cell lines by quantitative RT-PCR analysis. Kruskal-Wallis with Dunn's multiple comparisons test was used for analysis of the data. n = 3 biological replicates. Data are presented as mean ± s.d.. **(c)** Representative western-blotting images showing the protein expression of integrin β1 and integrin α3 in different cell lines. Three independent experiments were performed. (**d**) Representative flow cytometry results showing the cell surface protein expression of integrin β1 and integrin α3 in HuH-7, HeLa, MCF-7 and Ect1/E6E7 cell lines. Three independent experiments were performed. (**e-f**) Representative images and quantitative analysis showing attached HuH-7 cells in plates coated with or without 100 μM FFFIKLLI. Scale bar represents 200 μm. n = 30 biological replicates. Two-sided Mann-Whitney test was used for analysis of the data. Data are presented as mean ± s.d.. (**g-i**) Representative western-blotting images showing the knockdown efficiency of integrins in HuH-7. At least four independent experiments were performed. **(j, k)** 48 hr wound healing rate and the representative images of peptide FFFIKLLI treatment on integrin knockdown HuH-7 cells. Peptide concentration, 100 μM. Scale bar represents 200 μm. Kruskal-Wallis with Dunn's multiple comparisons test was used for analysis of the data. n = 6 biological replicates. Data are presented as mean ± s.d.. (**l, m**) Representative images and quantitative analysis showing attached HuH-7 cells in plates coated with 100 μM FFFIKLLI. Scale bar represents 200 μm. n = 30 biological replicates. Kruskal-Wallis with Dunn's multiple comparisons test was used for analysis of the data. Data are presented as mean ± s.d.. Source numerical data are available in source data.

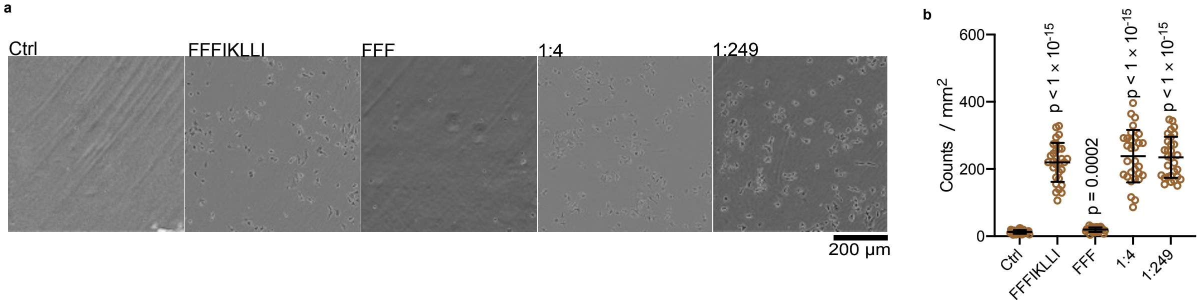

Supplementary Figure 5. Effects of peptide assembly on cell adhesion.

Representative images (**a**) and quantitative analysis (**b**) showing attached HuH-7 cells in plates coated with peptide self-assembly or co-assembly. The concentration of FFFIKLLI is kept 100 μM in every mixture. Scale bar represents 200 μm. n = 30 biological replicates. Kruskal-Wallis with Dunn's multiple comparisons test was used for analysis of the data. Data are presented as mean ± s.d.. Source numerical data are available in source data.

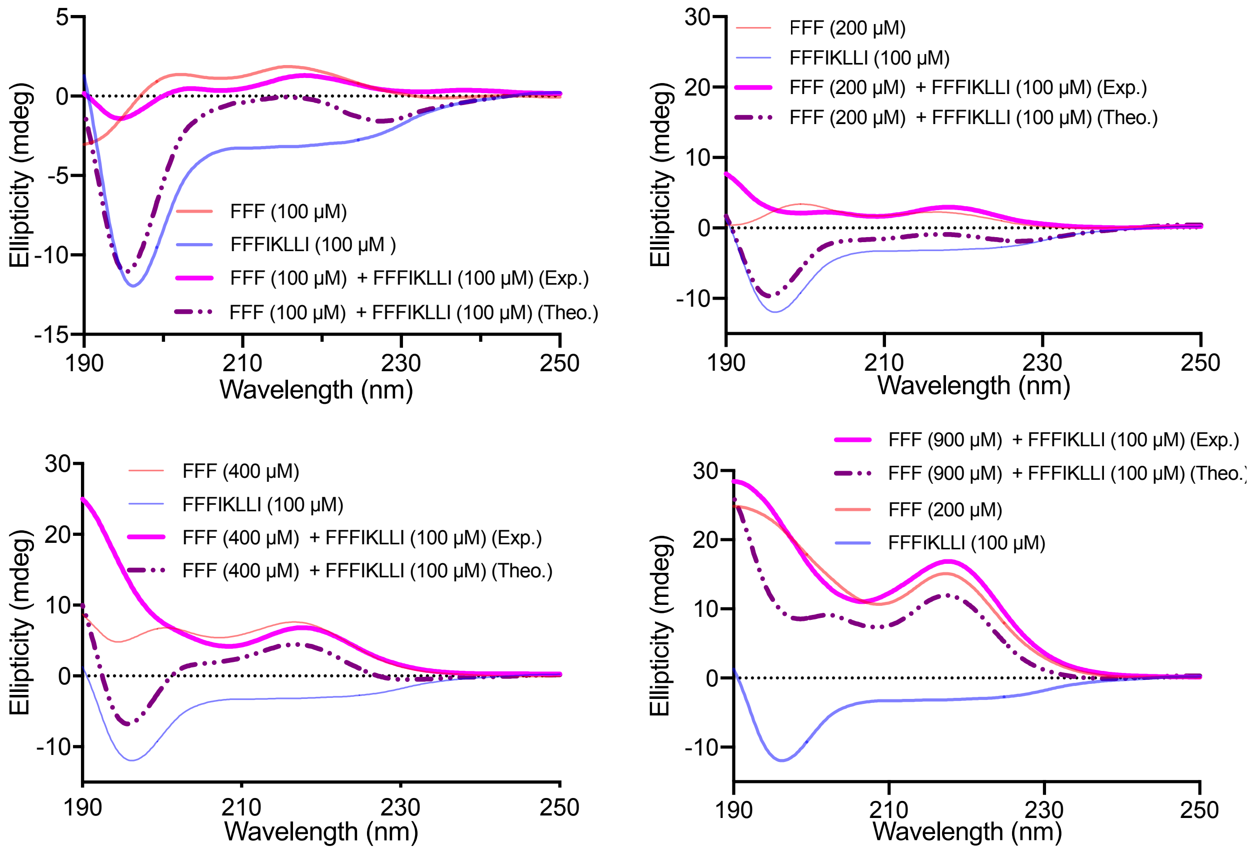

Supplementary Figure 6. CD spectroscopy of peptide assembly.

CD spectra of FFFIKLLI (100 μM), FFF (100, 200, 400, and 900 μM), and their mixtures. Exp. represents the experimental spectra of the mixture, while Theo. represents the simple sum of single component CD spectra of FFFIKLLI and FFF. Three independent experiments were performed. Source numerical data are available in source data.

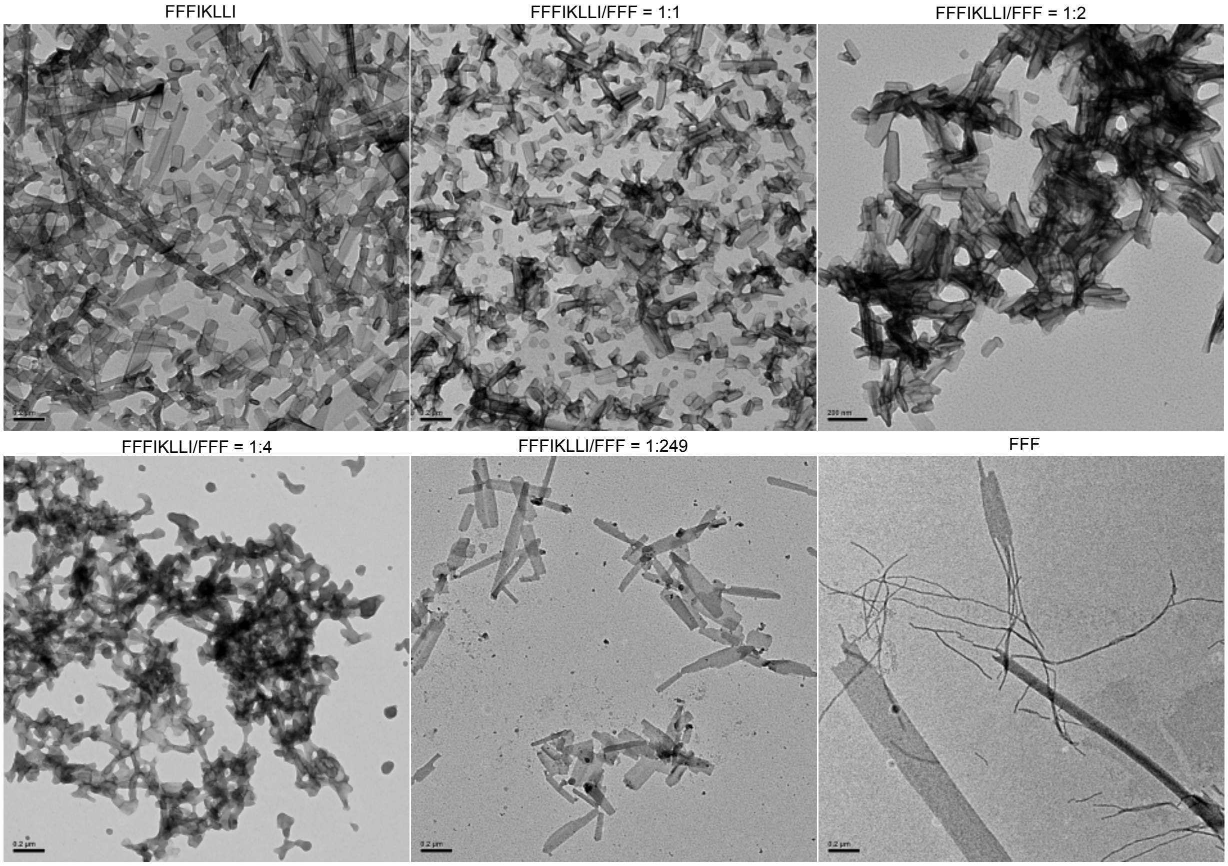

Supplementary Figure 7. TEM images of peptide assembly.

Representative TEM images of nanofilaments formed via peptide self-assembly or co-assembly in water. The concentration of FFFIKLLI is kept 100 μM in every mixture. Self-assembly of FFF was imaged in solution of 100 μM FFF. Scale bar, 200 nm. At least three independent experiments were performed.

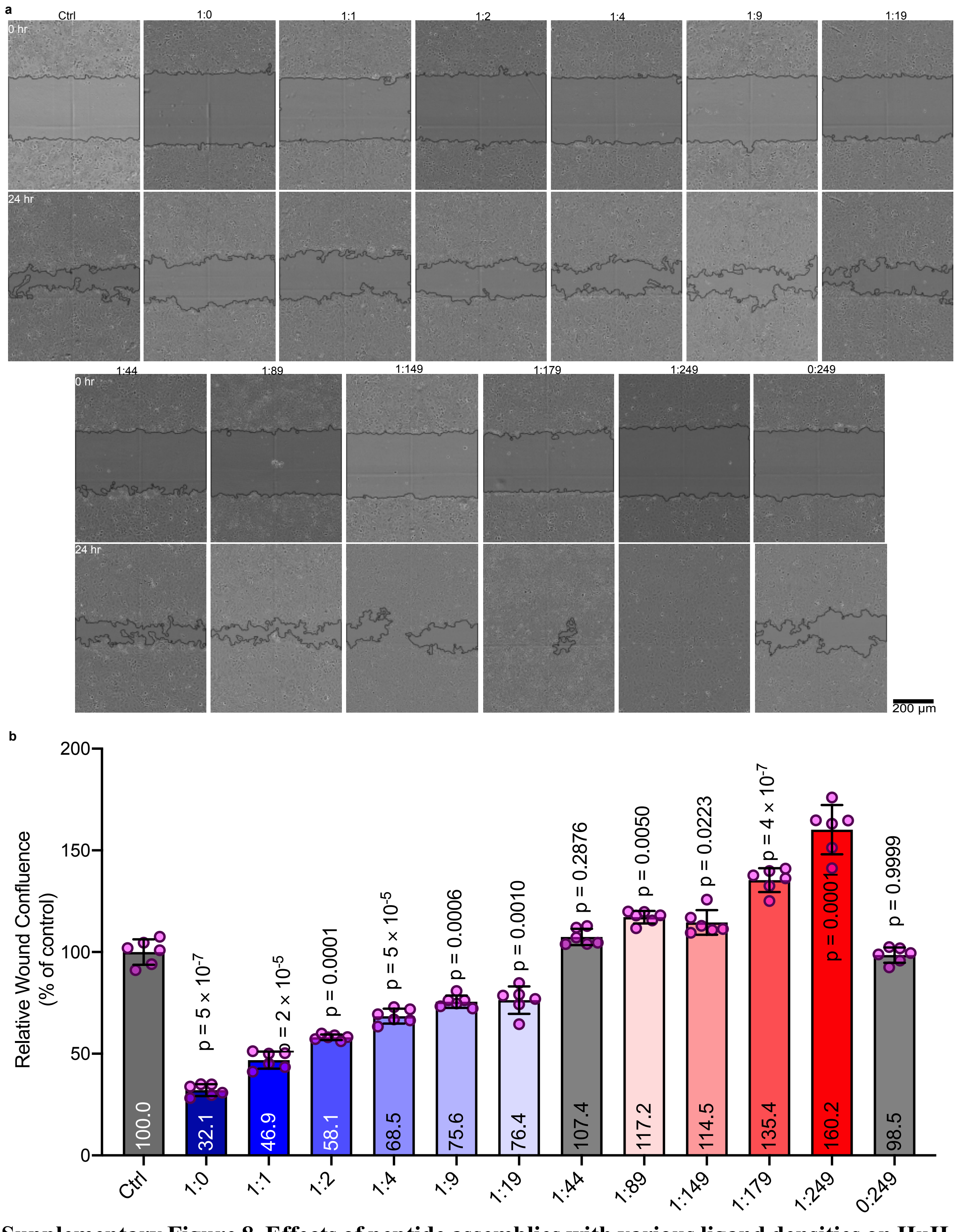

Supplementary Figure 8. Effects of peptide assemblies with various ligand densities on HuH-7 cell migration.

Representative images (**a**) and relative wound closure rate (**b**) of HuH-7 cells upon the treatment of co-assembled FFFIKLLI (fixed at 100 μM) and FFF at various ratios. Kruskal-Wallis with Dunn's multiple comparisons test was used for analysis of the data. n = 6 biological replicates. Data are presented as mean ± s.d.. Source numerical data are available in source data.

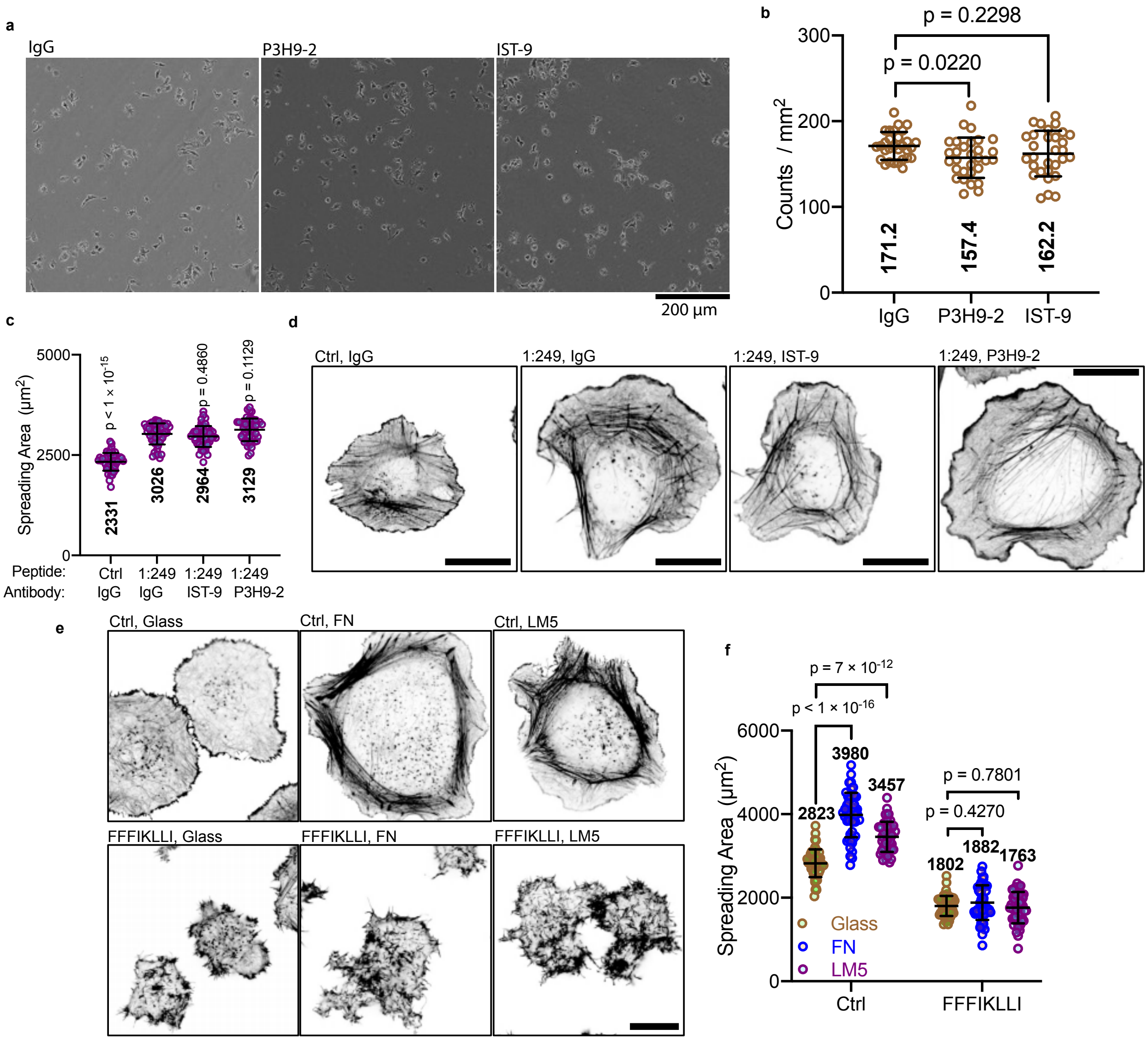

Supplementary Figure 9. Effects of FFFIKLLI on HuH-7 cells without certain cell secreted ECM components.

(**a, b**) Cell adhesion assay was performed by seeding HuH-7 cells on FFFIKLLI pre-coated dishes, and treated with IgG (Ctrl), laminin-5 function-inhibitory antibody P3H9-2, and fibronectin-blocking antibody IST-9. Representative images are shown in (**a**), and the quantitative results are presented in (**b**). Kruskal-Wallis with Dunn's multiple comparisons test was used for analysis of the data. n = 30 biological replicates. Data are presented as mean ± s.d.. (**c, d**) Cell spreading evaluation was performed by treating the HuH-7 cells with IgG (Ctrl), FFFIKLLI/FFF (1/249) and IgG, FFFIKLLI/FFF (1/249) and IST-9, FFFIKLLI/FFF (1/249) and P3H9-2 for 12 hr. Representative images are shown in (**c**), scale bar represents 10 µm, and the quantitative results are presented in (**d**). At least three independent experiments were performed. Kruskal-Wallis with Dunn's multiple comparisons test was used for analysis of the data. n = 50 cells. Data are presented as mean ± s.d.. **(e, f)** Cell spreading evaluation was performed by seeding HuH-7 cells on glass, fibronectin (FN) coated dish, LM-5 coated dish, culturing for 4 hr, followed by the treatment of FFFIKLLI for 12 hr. Representative images are shown in (**e**), scale bar represents 10 µm, and the

quantitative results are presented in (**f**). At least three independent experiments were performed. Two-sided Mann-Whitney test was used for analysis of the data. n = 50 cells. Data are presented as mean ± s.d.. Source numerical data are available in source data.

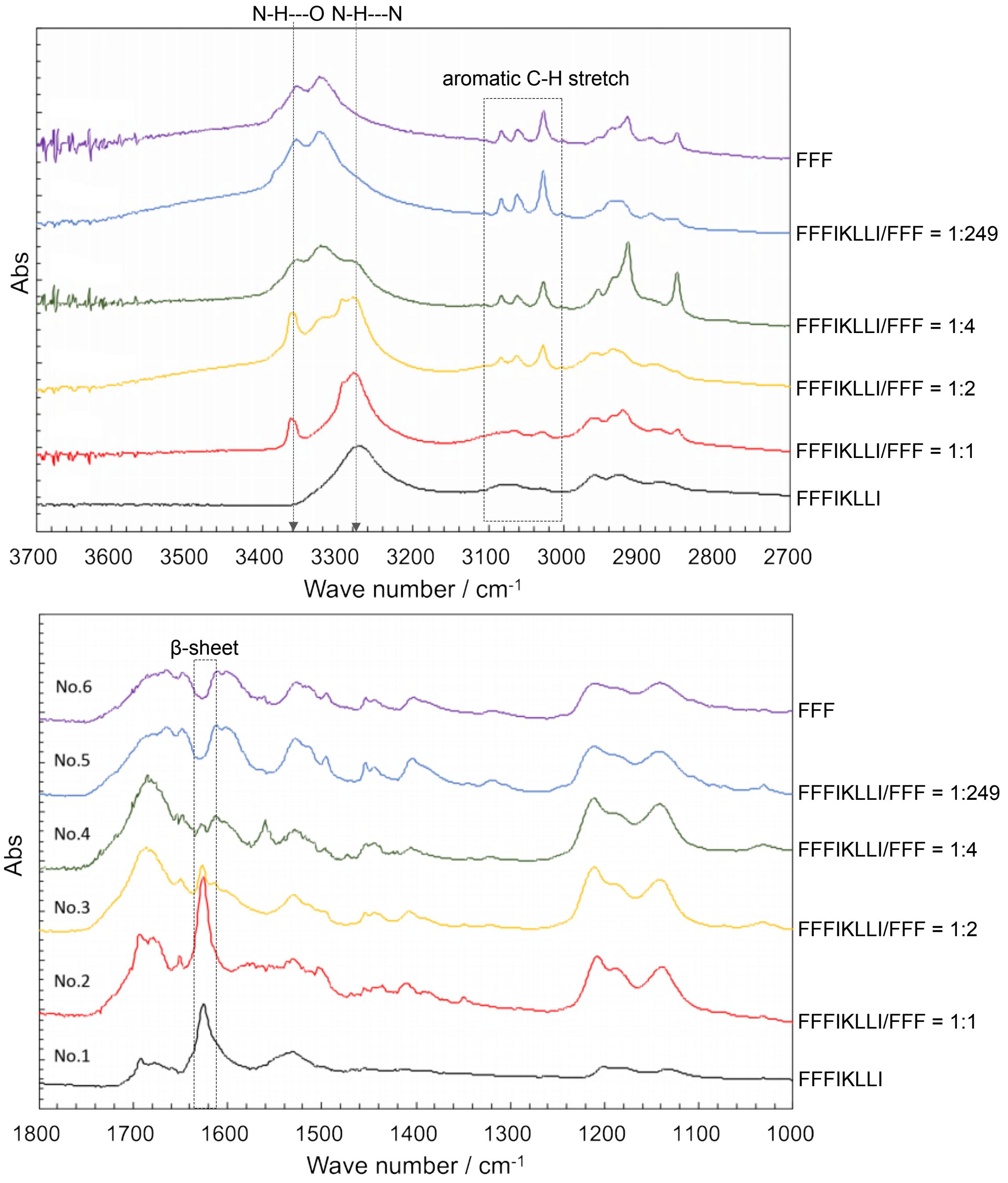

Supplementary Figure 10. FTIR spectroscopy of peptide assemblies with various ligand densities.

FTIR spectra of peptide assemblies of FFFIKLLI (100 µM), mixture of FFFIKLLI (100 µM) and FFF in various ratios, and FFF (100 µM) formed in aqueous solution at room temperature. Two independent experiments were performed.

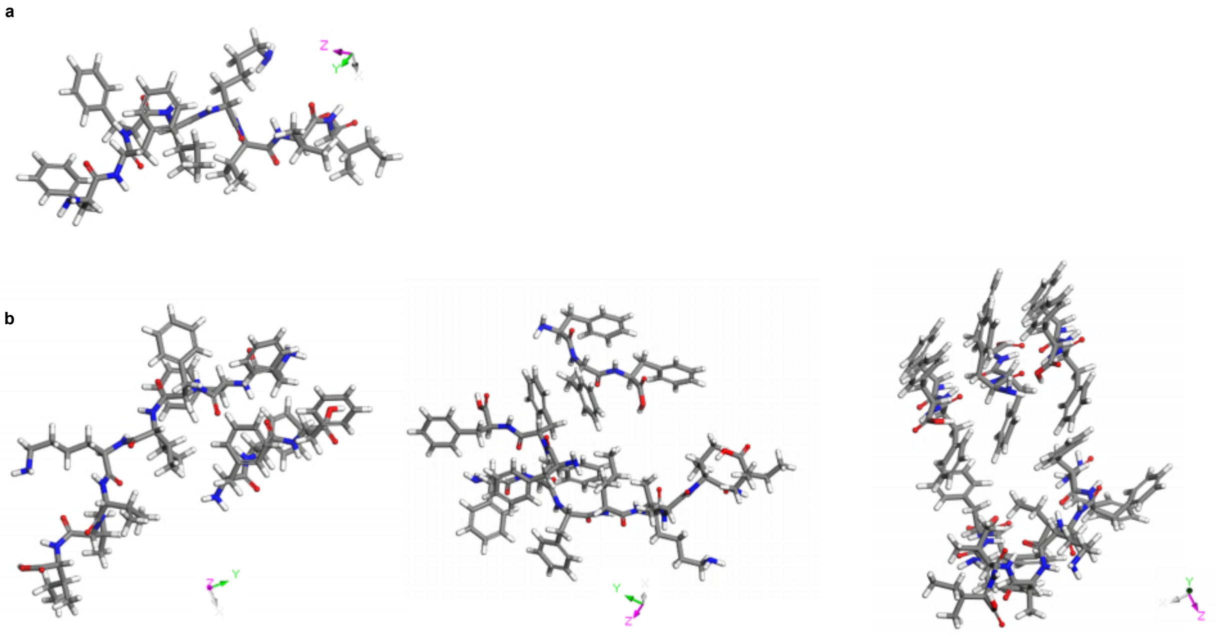

Supplementary Figure 11. Optimized molecular structures.
The molecular and geometry-optimized structures of FFFIKLLI (**a**), FFF and FFFIKLLI at 1:1, 1:2, and 1:4 ratio (**b**) presented in stick model.

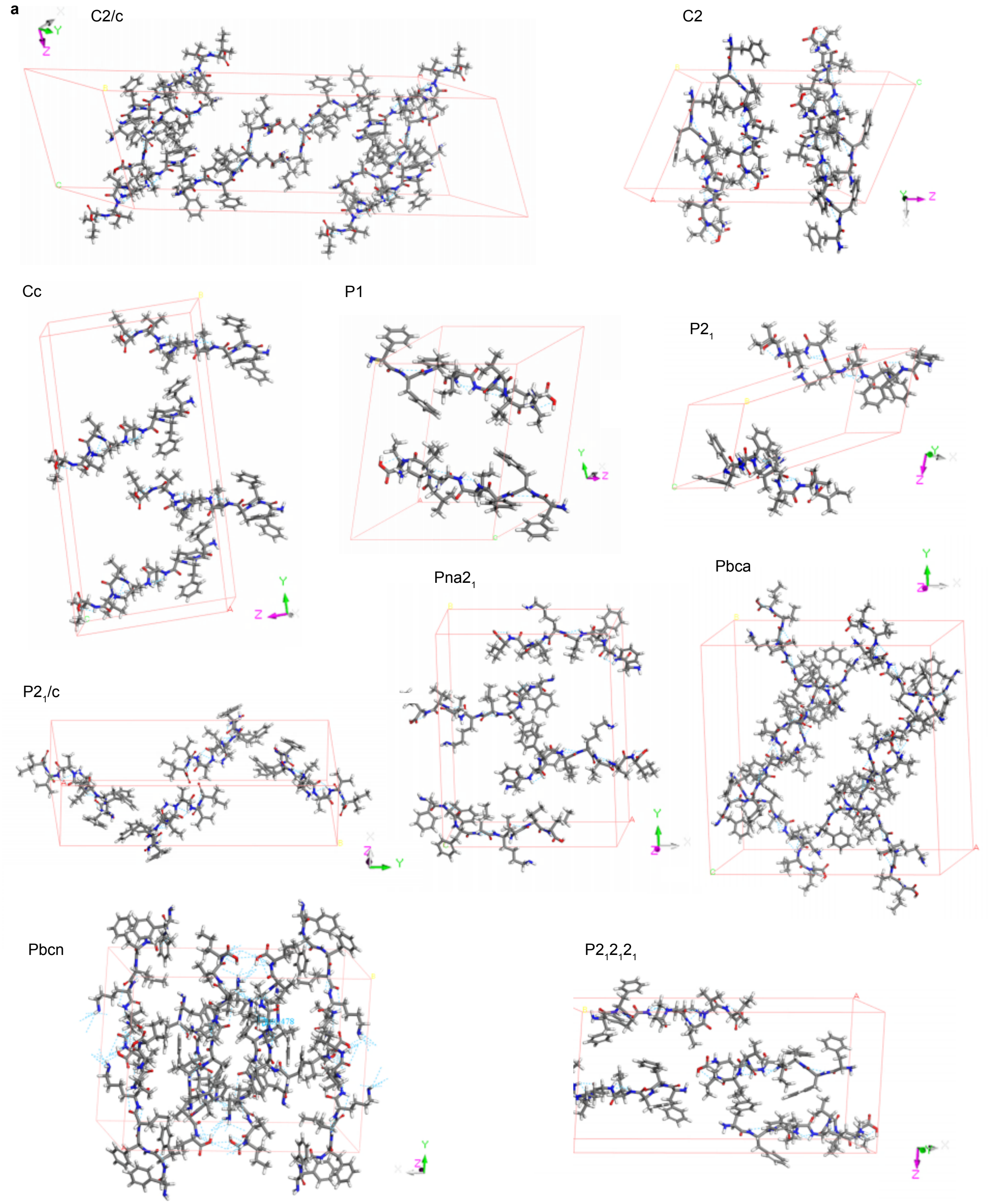
a
C2/c
C2
Cc
P1
P2$_1$
Pna2$_1$
Pbca
P2$_1$/c
Pbcn
P2$_1$2$_1$2$_1$

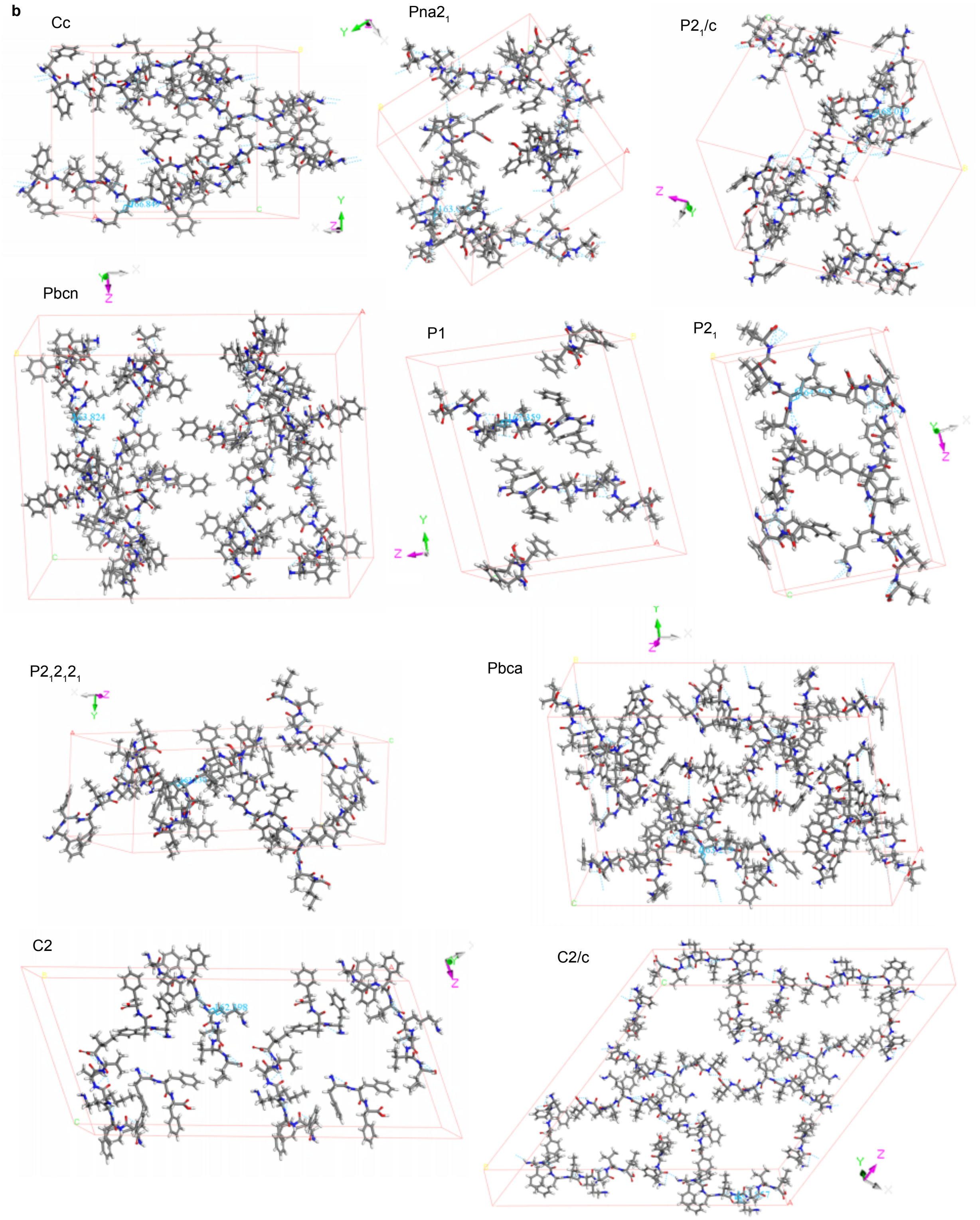
b
Cc
$Pna2_1$
$P2_1/c$
Pbcn
P1
$P2_1$
$P2_12_12_1$
Pbca
C2
C2/c

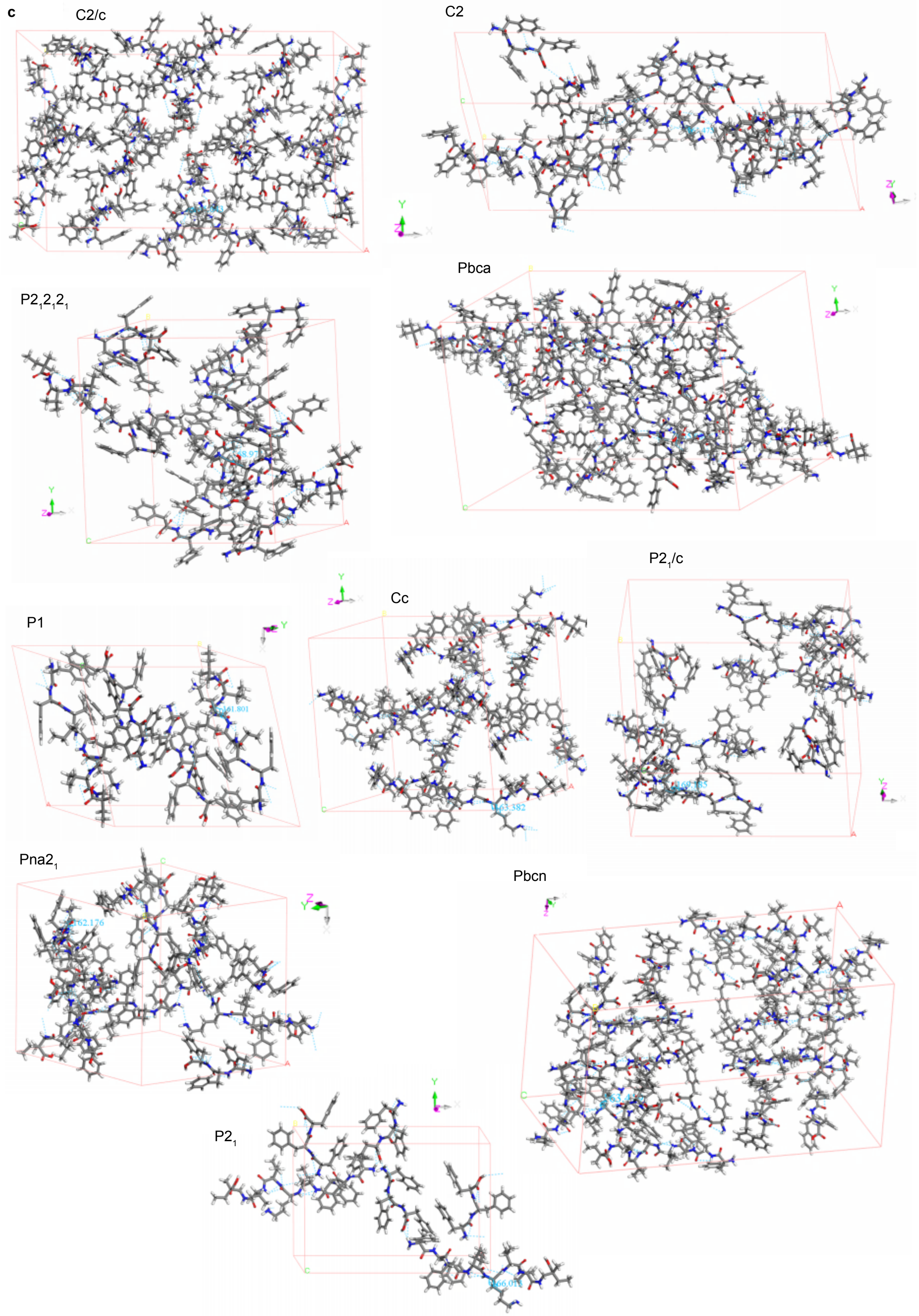
c
C2/c
C2
P2₁2₁2₁
Pbca
P1
Cc
P2₁/c
Pna2₁
Pbcn
P2₁

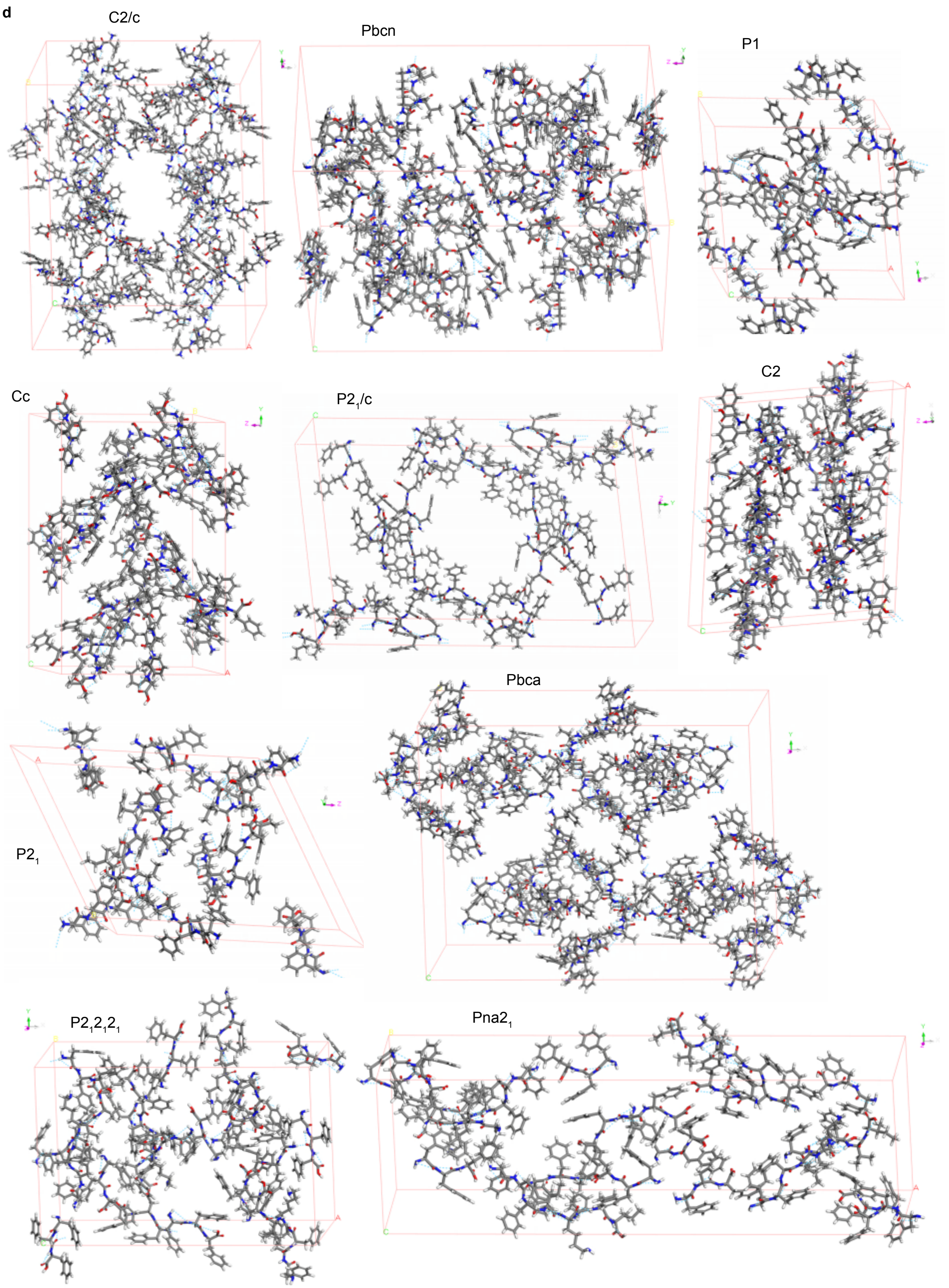
d
C2/c
Pbcn
P1
Cc
P2$_1$/c
C2
Pbca
P2$_1$
P2$_1$2$_1$2$_1$
Pna2$_1$

Supplementary Figure 12. Predicted crystal structure unit cells of peptide assembly.

Predicted crystal structure unit cell of FFFIKLLI (**a**), FFFIKLLI:FFF = 1:1 (**b**), FFFIKLLI:FFF = 1:2 (**c**), FFFIKLLI:FFF = 1:4 (**d**), with the $P2_1/c$, $P1$, $P2_12_12_1$, $P2_1$, $C2/c$, $Pbca$, $Pna2_1$, $Pbcn$, Cc, and $C2$ space group symmetry. The structures are presented in stick model.

Supplementary Table 1. Summary of inter-/intra-hydrogen bonding in peptide assembly. Hydrogen bonding analysis of predicted crystal structure unit cells. Intra represent intramolecular hydrogen bonding and inter represent intermolecular hydrogen bonding.

					a		b					
	FFF		FFFIKLLI:FFF=1:4		FFFIKLLI:FFF=1:2		FFFIKLLI:FFF=1:2		FFFIKLLI:FFF=1:1		FFFIKLLI	
Sapce group	NH-O	NH-N	NH-O	NH-N	NH-O	NH-N	NH-O	NH-N	NH-O	NH-N	NH-O	NH-N
XRD intra	P											
XRD inter	P											
C2-C intra			P		P	P	P		P	P	P	
C2-C inter					P		P					
C2 intra			P		P	P	P	P	P	P	P	
C2 inter			P								P	
CC intra			P		P	P	P		P	P	P	
CC inter					P		P					
P-1 intra			P		P		P		P		P	
P-1 inter				P					P			
P21-C intra			P		P	P	P	P	P			
P21-C inter			P	P	P		P					
P21 intra			P		P	P	P	P	P		P	P
P21 inter			P	P	P	P			P			
P212121 intra			P	P	P		P		P		P	P
P212121 inter			P	P	P		P				P	
PBCA intra			P		P	P	P	P	P			P
PBCA inter			P		P	P	P	P	P		P	
PBCN intra			P		P	P	P	P	P	P		P
PBCN inter				P	P		P		P			
PNA21 intra			P		P		P	P	P		P	P
PNA21 inter						P		P	P		P	P

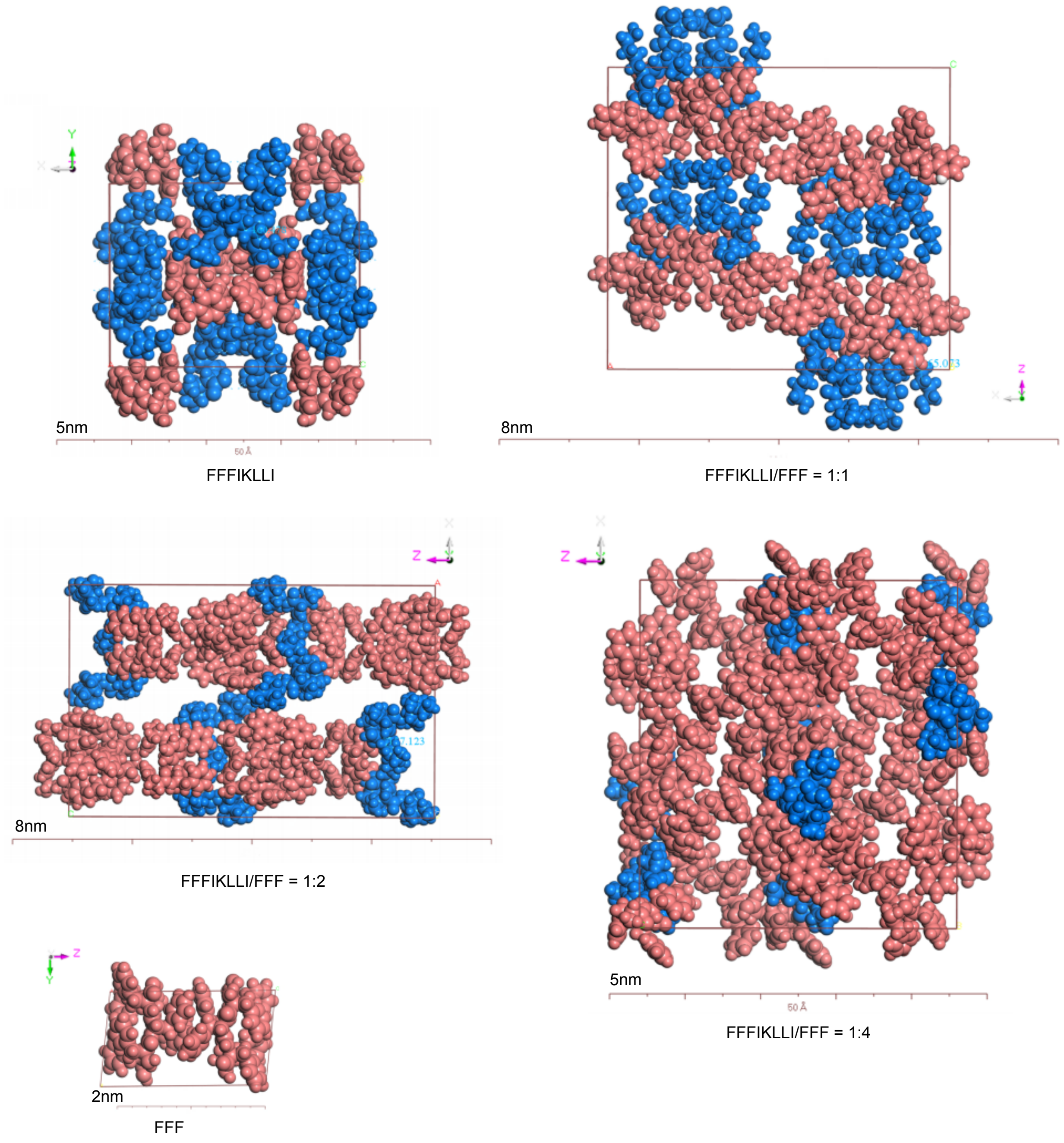

Supplementary Figure 13. Crystal unit cells of peptide assemblies.

Predictions of crystal structure unit cell with proper hydrogen bonding types matching to the FTIR analysis presented in space-filling model. Pink represents FFF motif, and blue represents IKLLI motif.

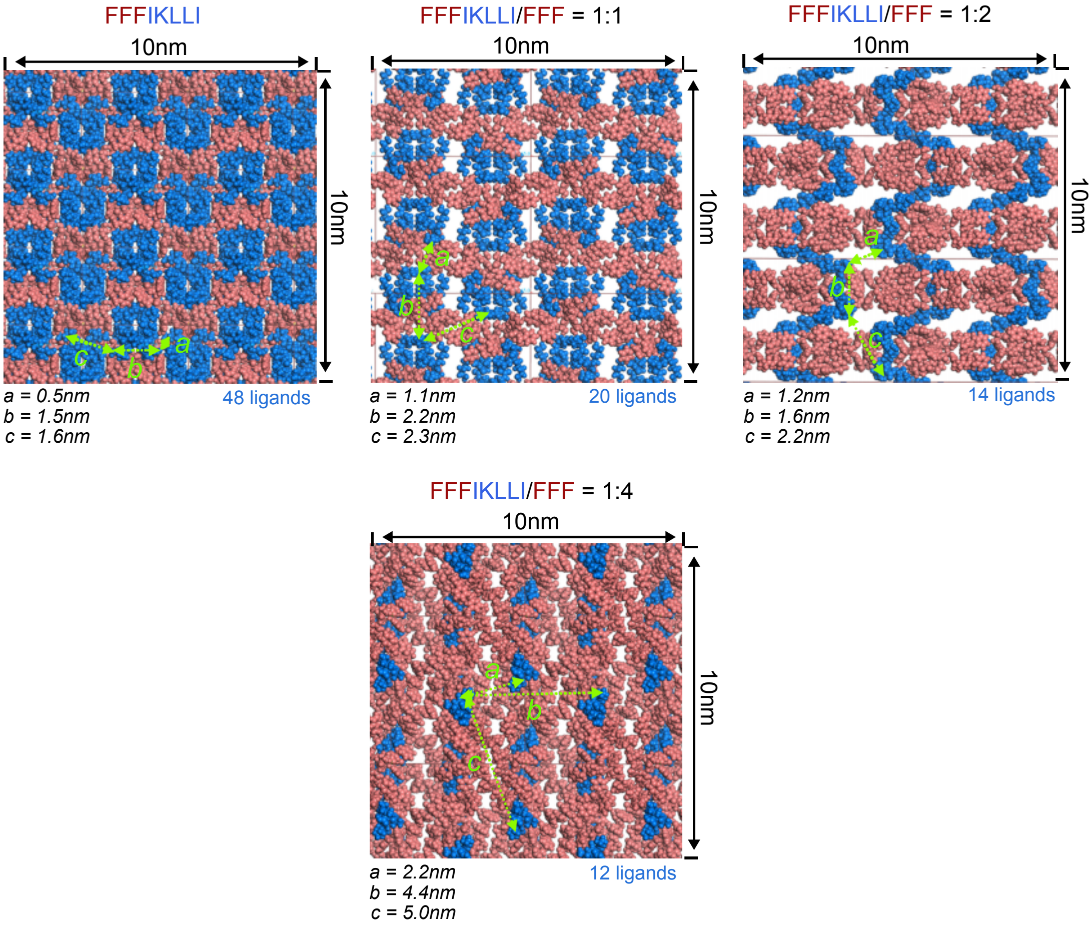

Supplementary Figure 14. Space-filling model of surface structure formed by extending the unit cells of polymorph predictions.

FFF motif was presented in pink, while IKLLI motif was presented in blue.

a

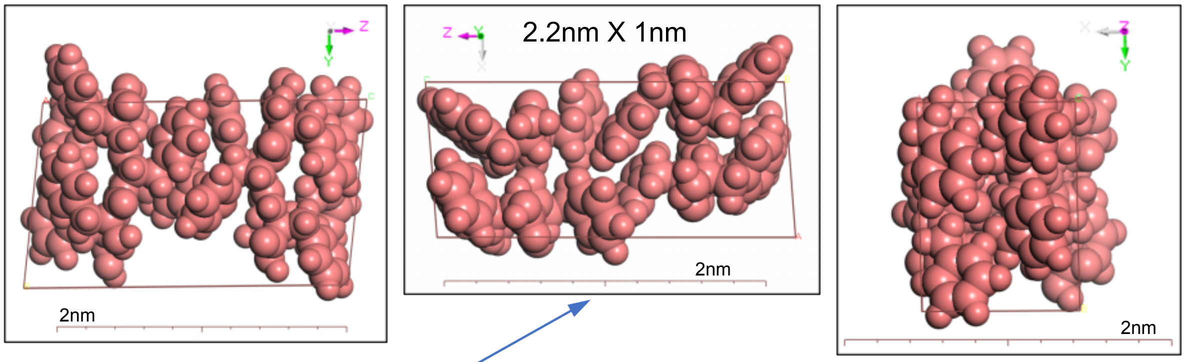

b

surface area of unit cell 2.2 nm^2
each unit cell contains 4 FFF

For FFFIKLLI : FFF = 1:249	2 FFFIKLLI per 125 unit cell<br>2/(125 x 2.2) x 137 = 1 ligand/137 nm^2
For FFFIKLLI : FFF = 1:179	1 FFFIKLLI per 45 unit cell<br>1/(45 x 2.2) x 100 = 1 ligand/100 nm^2
For FFFIKLLI : FFF = 1:149	2 FFFIKLLI per 75 unit cell<br>2/(75 x 2.2) x 100 = 1.2 ligand/100 nm^2
For FFFIKLLI : FFF = 1:89	2 FFFIKLLI per 45 unit cell<br>2/(45 x 2.2) x 100 = 2 ligand/100 nm^2
For FFFIKLLI : FFF = 1:44	4 FFFIKLLI per 45 unit cell<br>4/(45 x 2.2) x 100 = 4 ligand/100 nm^2

c

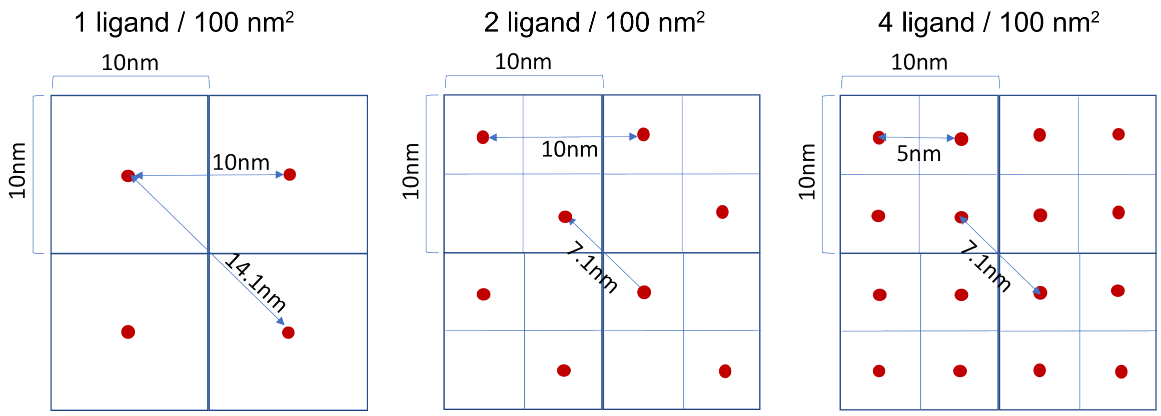

Supplementary Figure 15. Estimation of ligand density in various peptide assemblies.

(**a**) Space-filling model of crystal structure unit cell of FFF at zy, zx, xy plain. FFF motif was presented in pink. (**b**) The rough estimation of ligand density was carried out based on the conformation of the unit cell of FFF. The alteration of packing parameters induced by the insertion of FFFIKLLI was ignored since the majority of the packing molecules are FFF. Because the C-terminus of FFF that can be covalently linked with IKLLI is only exposed toward the zx plain (the image in the middle), we took the area size of this plain for surface estimation. The calculation

results of ligand density for FFFIKLLI/FFF at 1 to 249 to 1 to 44 were summarized. (**c**) The scheme represents the rough estimation of ligand distance in regard of three different densities.

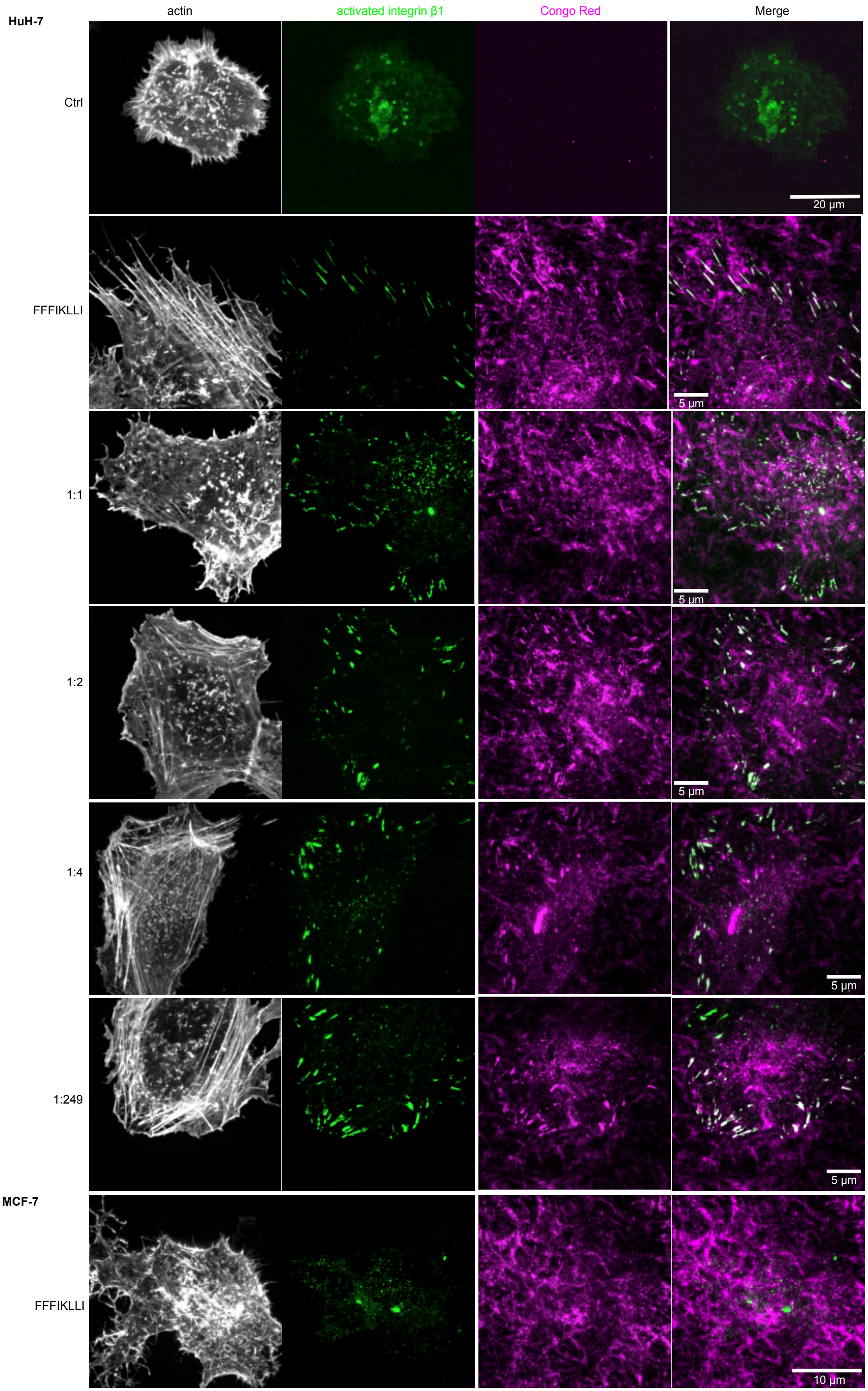
actin
activated integrin β1
Congo Red
Merge
HuH-7
Ctrl
20 μm
FFFIKLLI
5 μm
1:1
5 μm
1:2
5 μm
1:4
5 μm
1:249
5 μm
MCF-7
FFFIKLLI
10 μm

Supplementary Figure 16. Representative Immunofluorescence of activated integrin β1 (12G10), co-stained with Congo red and phalloidin upon various treatments.

Gray: phalloidin; green: anti-activated integrin β1; magenta: Congo red. At least three independent experiments were performed.

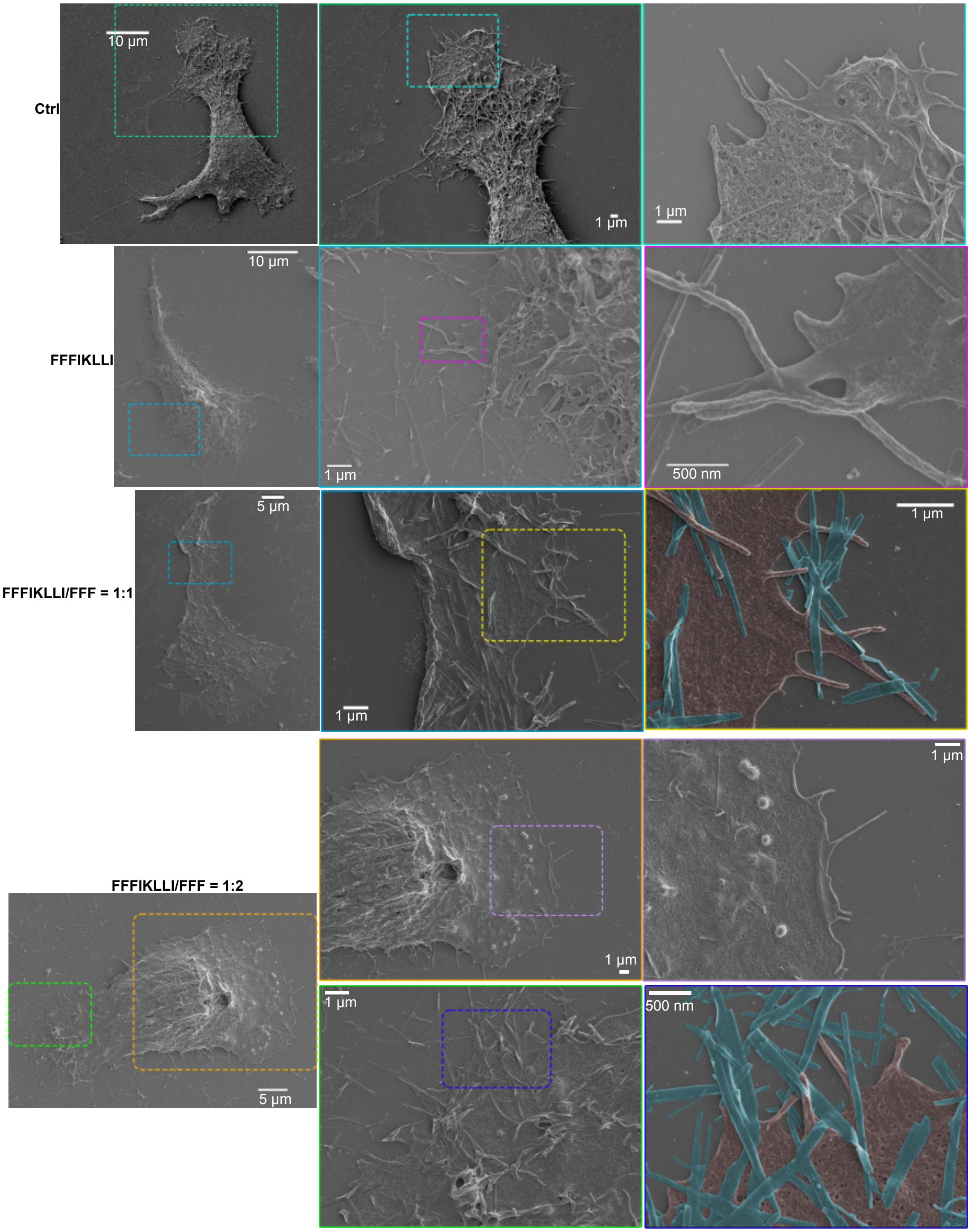

Ctrl
10 μm
1 μm
1 μm
FFFIKLLI
10 μm
1 μm
500 nm
FFFIKLLI/FFF = 1:1
5 μm
1 μm
1 μm
FFFIKLLI/FFF = 1:2
5 μm
1 μm
1 μm
1 μm
500 nm

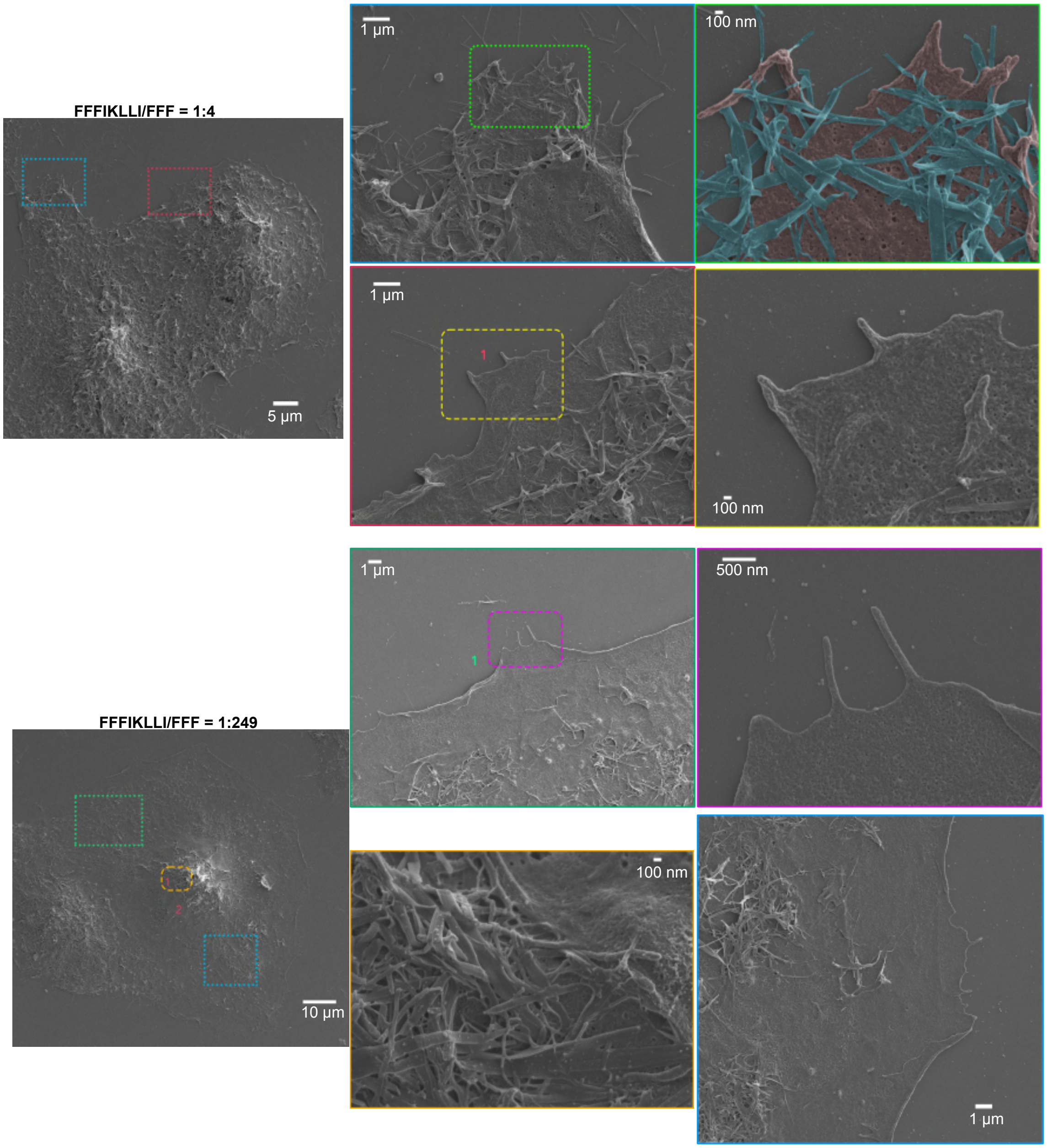

Supplementary Figure 17. SEM imaging of peptide-assembly-treated HuH-7 cell.
Representative SEM images of HuH-7 cell with or without the treatment of nanofilaments formed by FFFIKLLI (100 μM) and FFF at various ratios for 12 hr. Cell bodies were highlighted in pink and the nanofilaments were highlighted in blue for false color. Two independent experiments were performed.

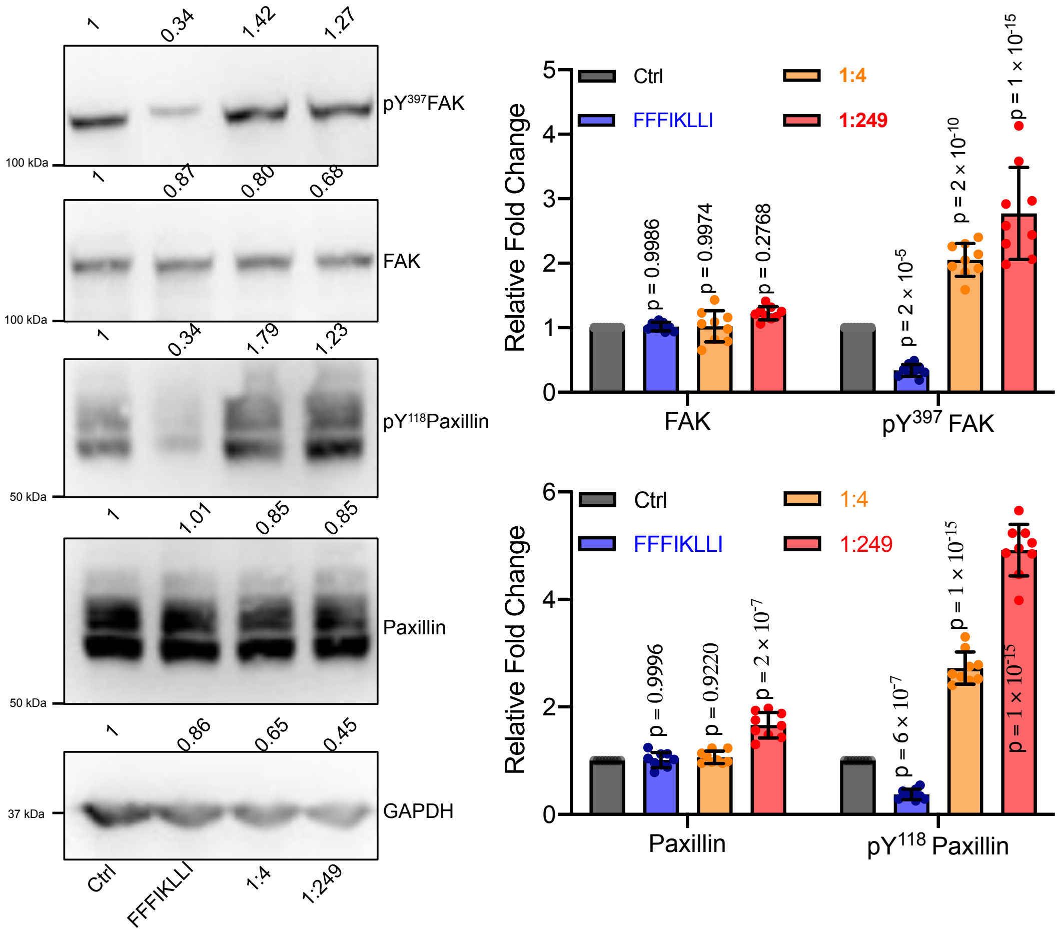

Supplementary Figure 18. Protein expression of HuH-7 cells upon various treatments. Protein expression of pY397 FAK, FAK, pY118 Paxillin, Paxillin and GADPH in HuH-7 cells treated with or without peptide assembly for 12 hr Kruskal-Wallis with Dunn's multiple comparisons test was used for analysis of the data. n = 9 biological replicates. Data are presented as mean ± s.d.. Source numerical data are available in source data.

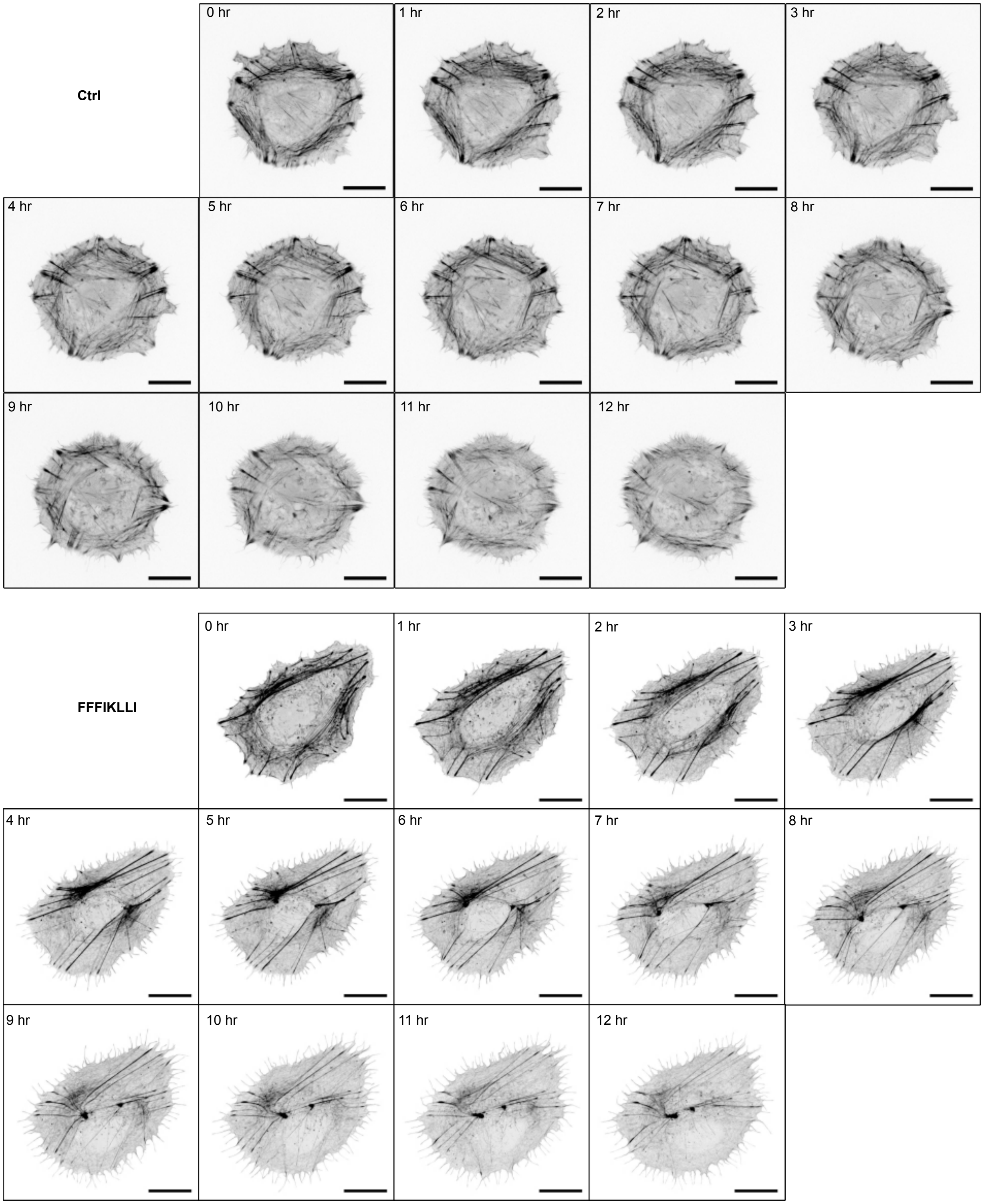
Ctrl
0 hr
1 hr
2 hr
3 hr
4 hr
5 hr
6 hr
7 hr
8 hr
9 hr
10 hr
11 hr
12 hr
FFFIKLLI
0 hr
1 hr
2 hr
3 hr
4 hr
5 hr
6 hr
7 hr
8 hr
9 hr
10 hr
11 hr
12 hr

FFFIKLLI/FFF = 1:1

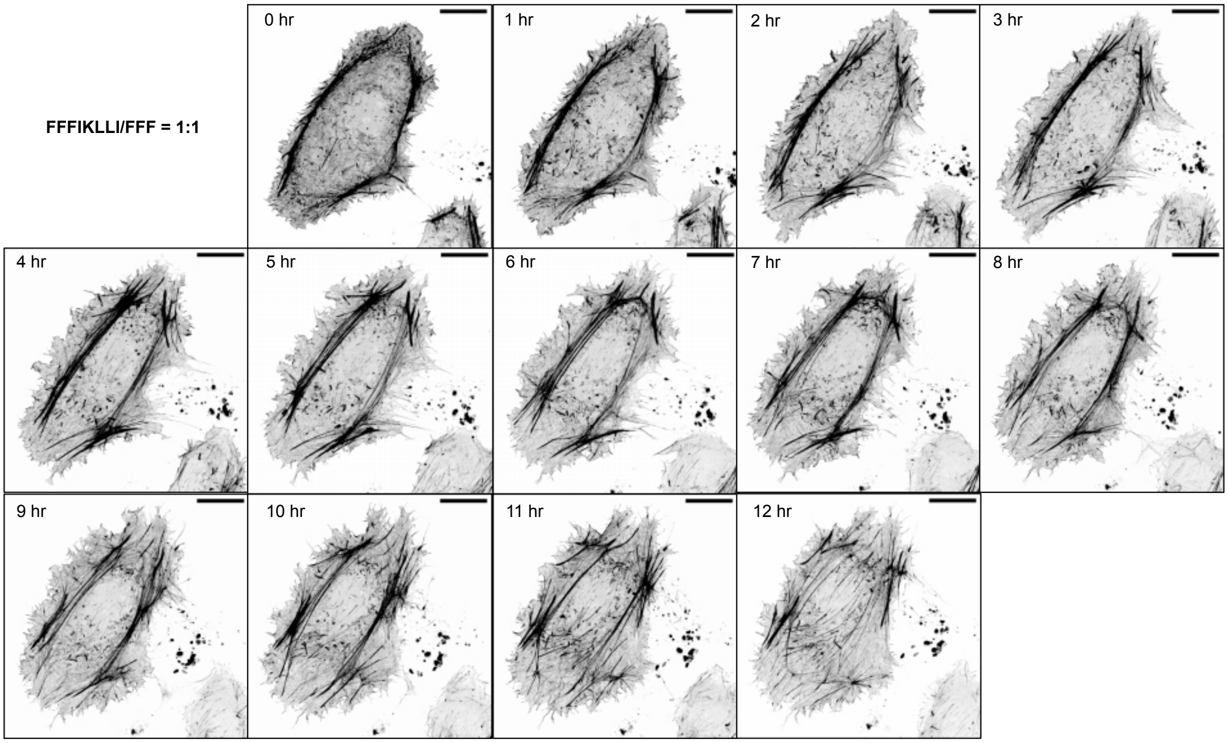

FFFIKLLI/FFF = 1:2

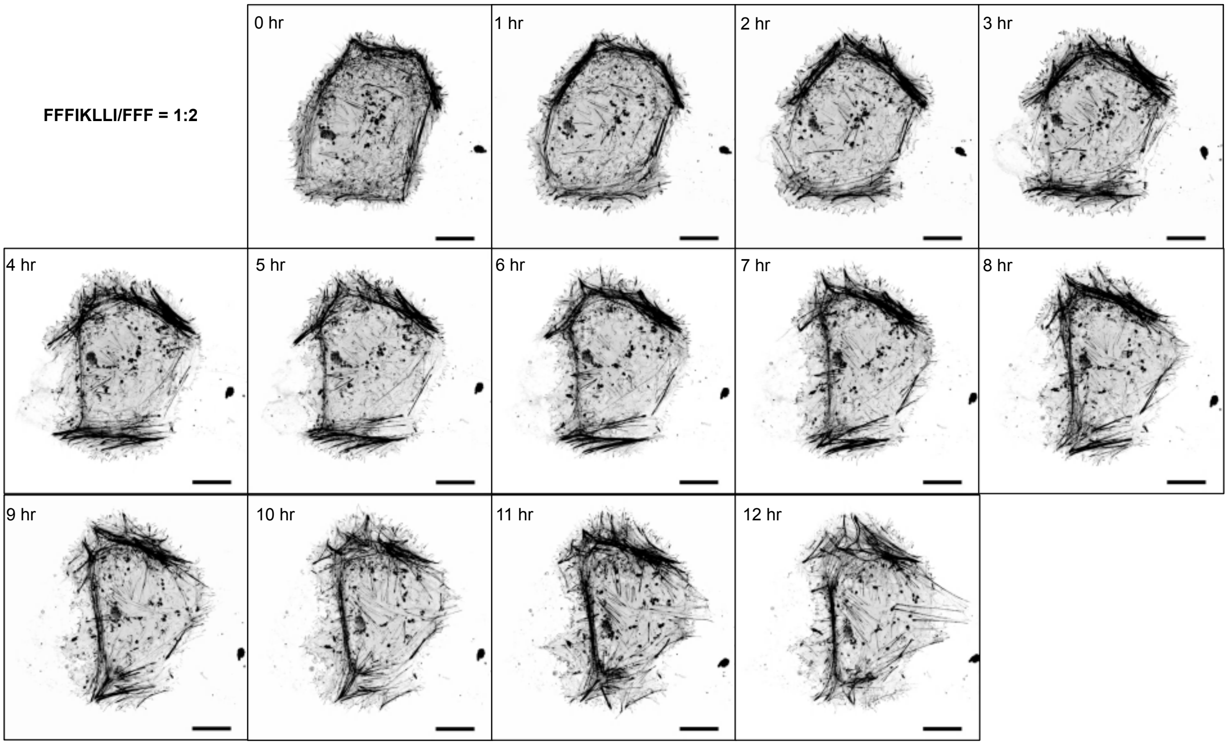

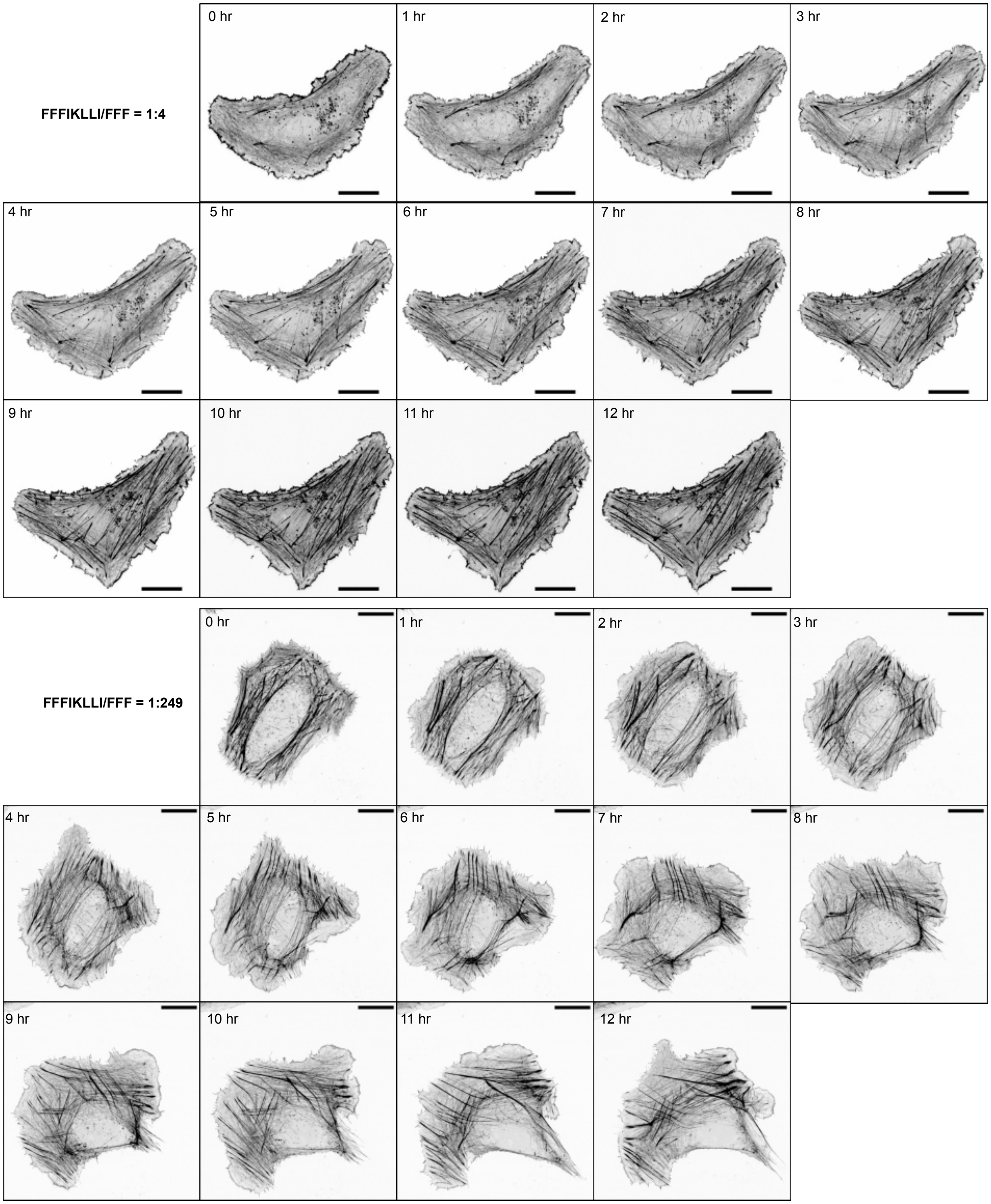
FFFIKLLI/FFF = 1:4
0 hr
1 hr
2 hr
3 hr
4 hr
5 hr
6 hr
7 hr
8 hr
9 hr
10 hr
11 hr
12 hr
FFFIKLLI/FFF = 1:249
0 hr
1 hr
2 hr
3 hr
4 hr
5 hr
6 hr
7 hr
8 hr
9 hr
10 hr
11 hr
12 hr

Supplementary Figure 19. **Time-lapse images of mRuby-Lifeact-7 transfected HuH-7 cells upon treatments.**

Representative time-lapse images of mRuby-Lifeact-7 transfected HuH-7 cells with the treatment of nanofilaments for 12 hr. Scale bars represent 20 μm. Three independent experiments were performed.

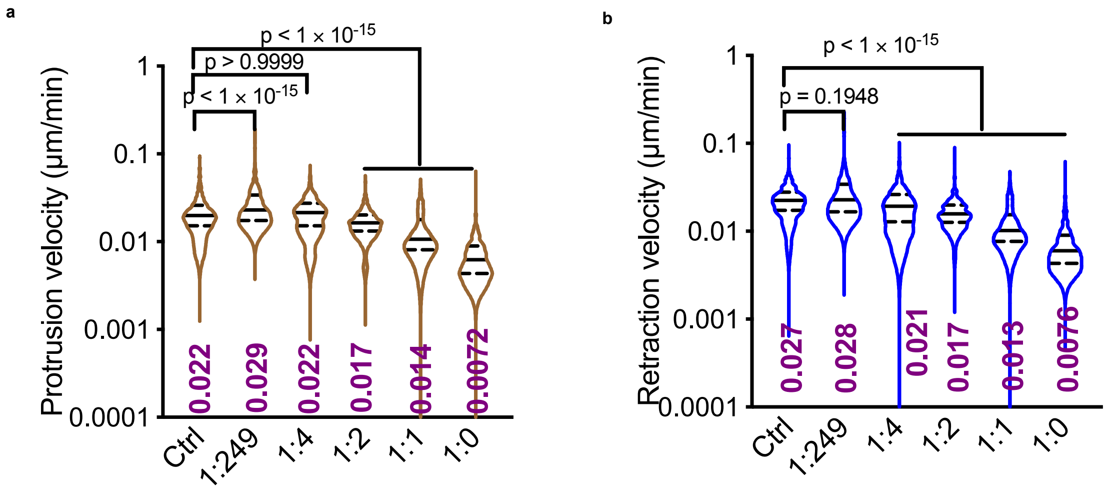

Supplementary Figure 20. Protrusion and retraction velocity of HuH-7 cells upon treatments.

Violin plot of all protrusion (**a**) or retraction velocity (**b**) values collected from each time point for every cell. Kruskal-Wallis with Dunn's multiple comparisons test was used for analysis of the data. Median and quartiles were presented in the plot. n = 8, 7, 10, 9, 9, 9 cells. Source numerical data are available in source data.

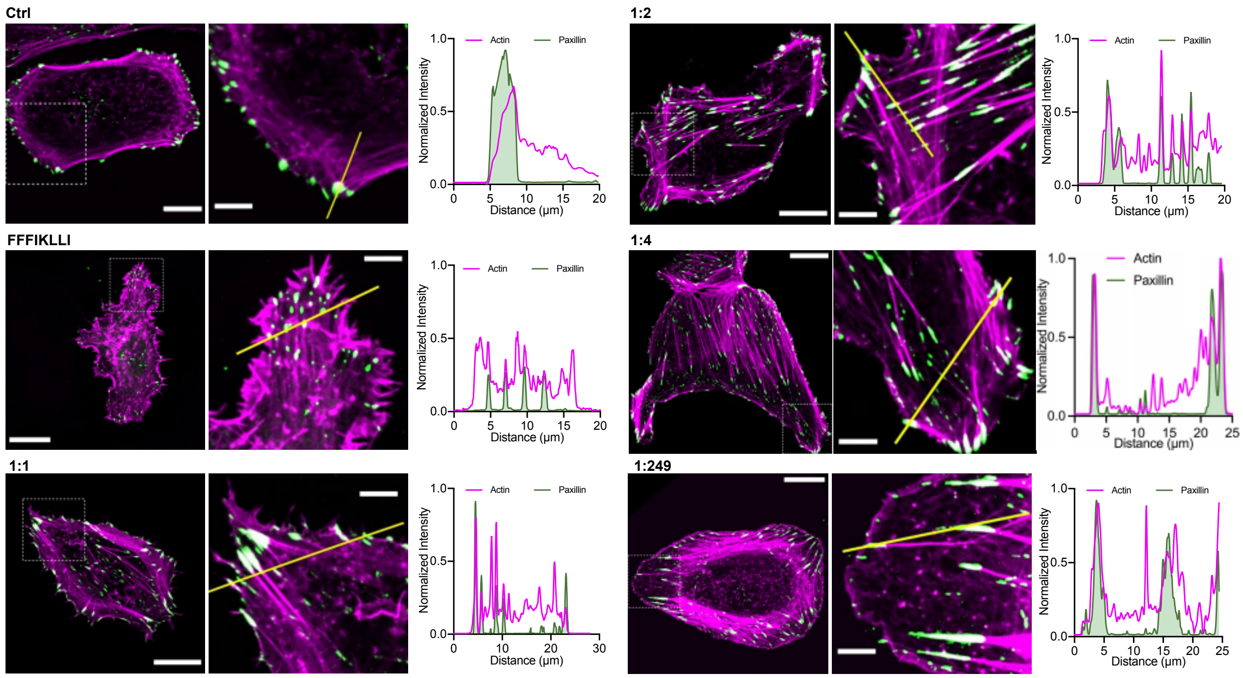

Supplementary Figure 21. Immunofluorescence of paxillin co-stained with phalloidin on HuH-7 cells.

Immunofluorescence of paxillin co-stained with phalloidin for each treatment condition. Green: anti-paxillin; magenta: phalloidin. Scale bar represents 20 µm (the first column) and 5 µm (the second column), respectively. The third column, intensity profiles. Three independent experiments were performed. Source numerical data are available in source data.

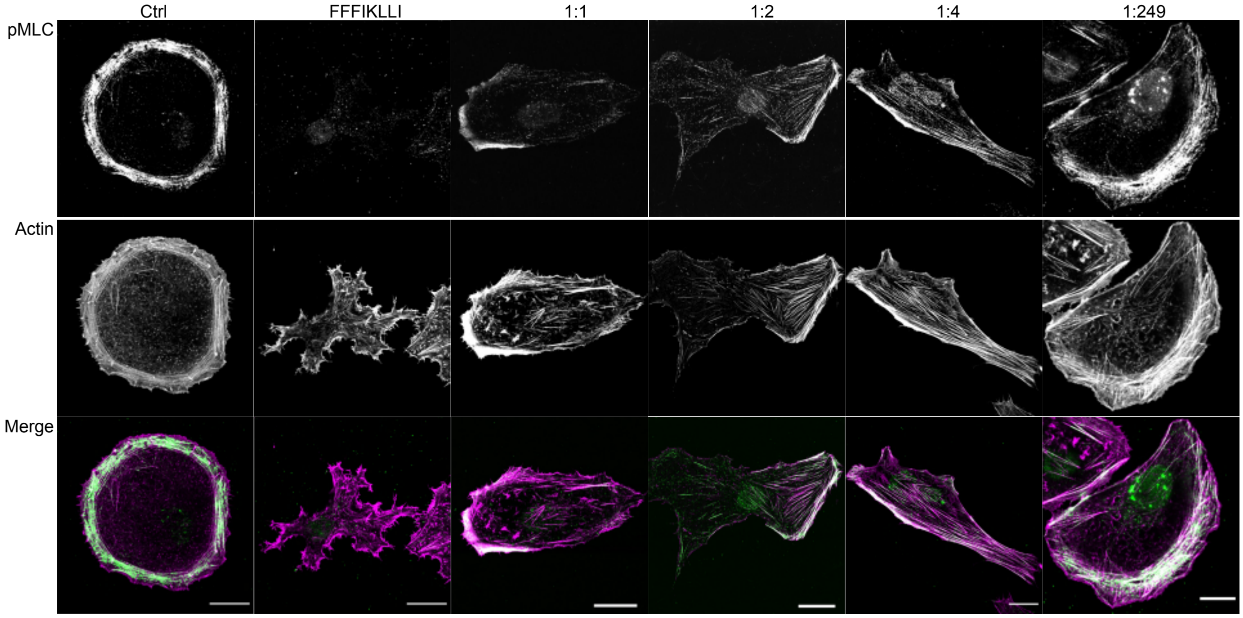

Supplementary Figure S22. Immunofluorescence of phospho-Myosin Light Chain 2 (pMLC) co-stained with phalloidin on HuH-7 cells.

Immunofluorescence of phospho-Myosin Light Chain 2 (pMLC) co-stained with phalloidin for each treatment condition. The first row, immunofluorescence of pMLC; the second row, phalloidin staining; the third row, merge images, green: anti-pMLC, magenta, phalloidin. Scale bars represent 20 µm. Three independent experiments were performed.

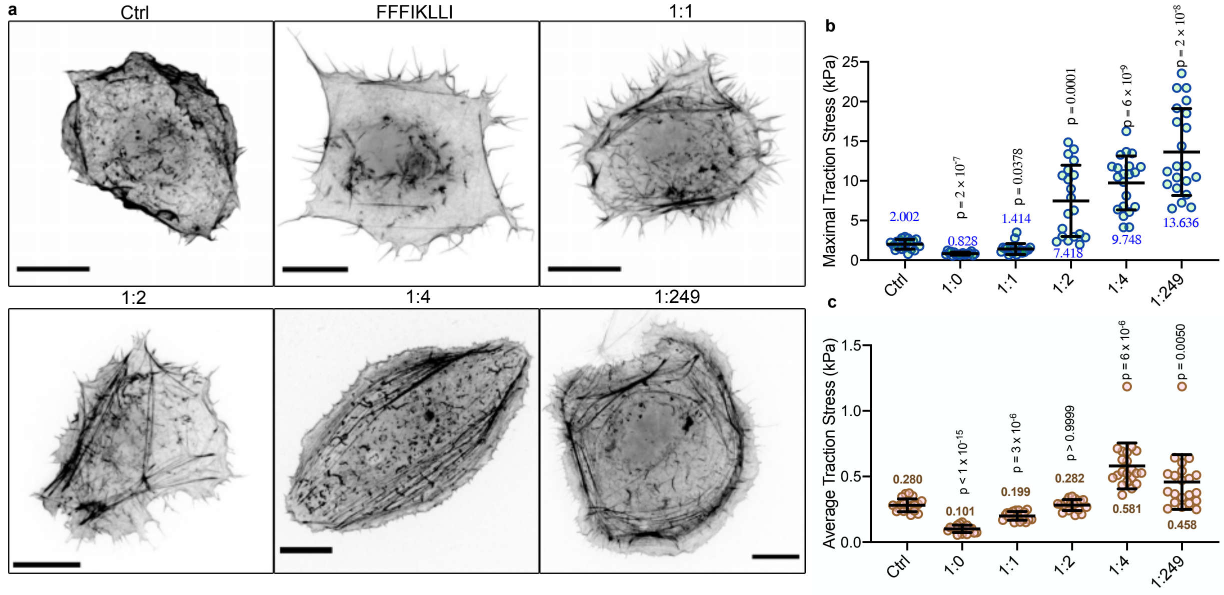

Supplementary Figure 23. Peptide assemblies with various ligand densities control the cell traction force.

(**a**) Representative images showing the morphology of HuH-7 cells cultured on PDMS subtracts. Cell was transfected with mRuby-Lifeact-7 and treated with peptide assemblies for 12 hr. Scale bar, 20 μm. (**b-c**) Average and Maximal traction stress of each condition. The average traction stress represents the mean traction force of each cell. Kruskal-Wallis with Dunn's multiple comparisons test was used for analysis of the data. Error bars represent standard deviation. n = 20 (Ctrl), 18 (1:0), 19 (1:1), 20 (1:2), 21 (1:4), 21 (1:249) cells. At least three independent experiments were performed. Data are presented as mean ± s.d.. Source numerical data are available in source data.

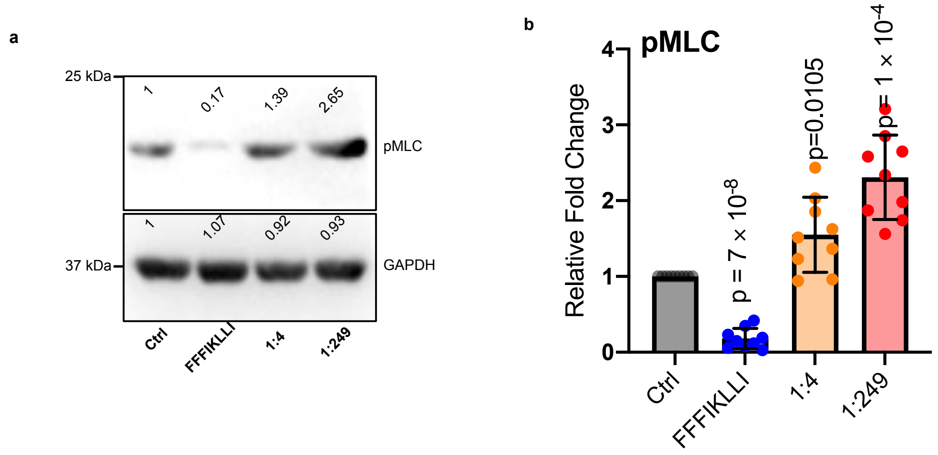

Supplementary Figure 24. Protein expression of Phospho-Myosin Light Chain 2 (Thr18/Ser19) (pMLC) and GADPH in HuH-7 cells.

Immunoblotting (**a**) analysis (**b**) of Phospho-Myosin Light Chain 2 (Thr18/Ser19) (pMLC) and GADPH expression in HuH-7 cells treated with or without peptide assembly for 12 hr. Kruskal-Wallis with Dunn's multiple comparisons test was used for analysis of the data. n = 9 biological replicates. Data are presented as mean ± s.d.. Source numerical data are available in source data.

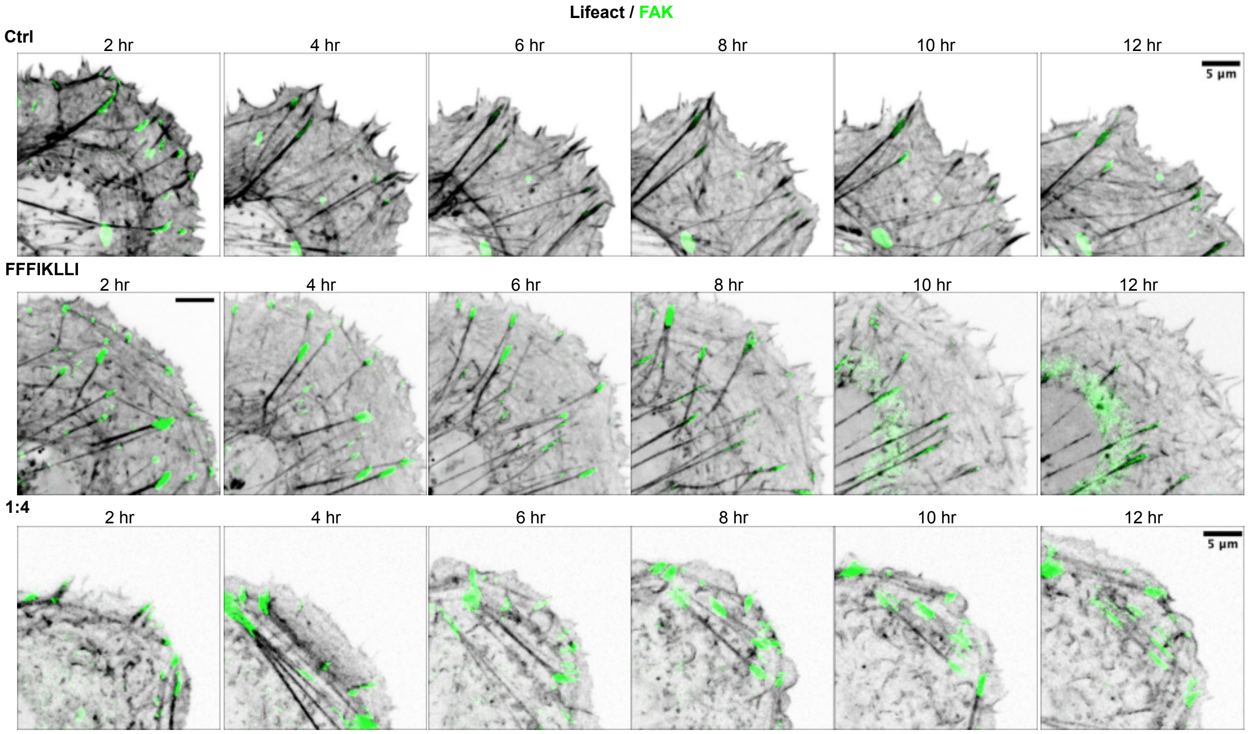

Supplementary Figure 25. The dynamics of actin cytoskeleton and FAK in HuH-7 cells.

Representative time-lapse images showing the dynamics of actin cytoskeleton (grey) and focal adhesion proteins (green) in HuH-7 cells co-transfected with pGFP-FAK upon the treatment of peptide assemblies for 12 hrs. Scale bars represent 5µm. Three independent experiments were performed.

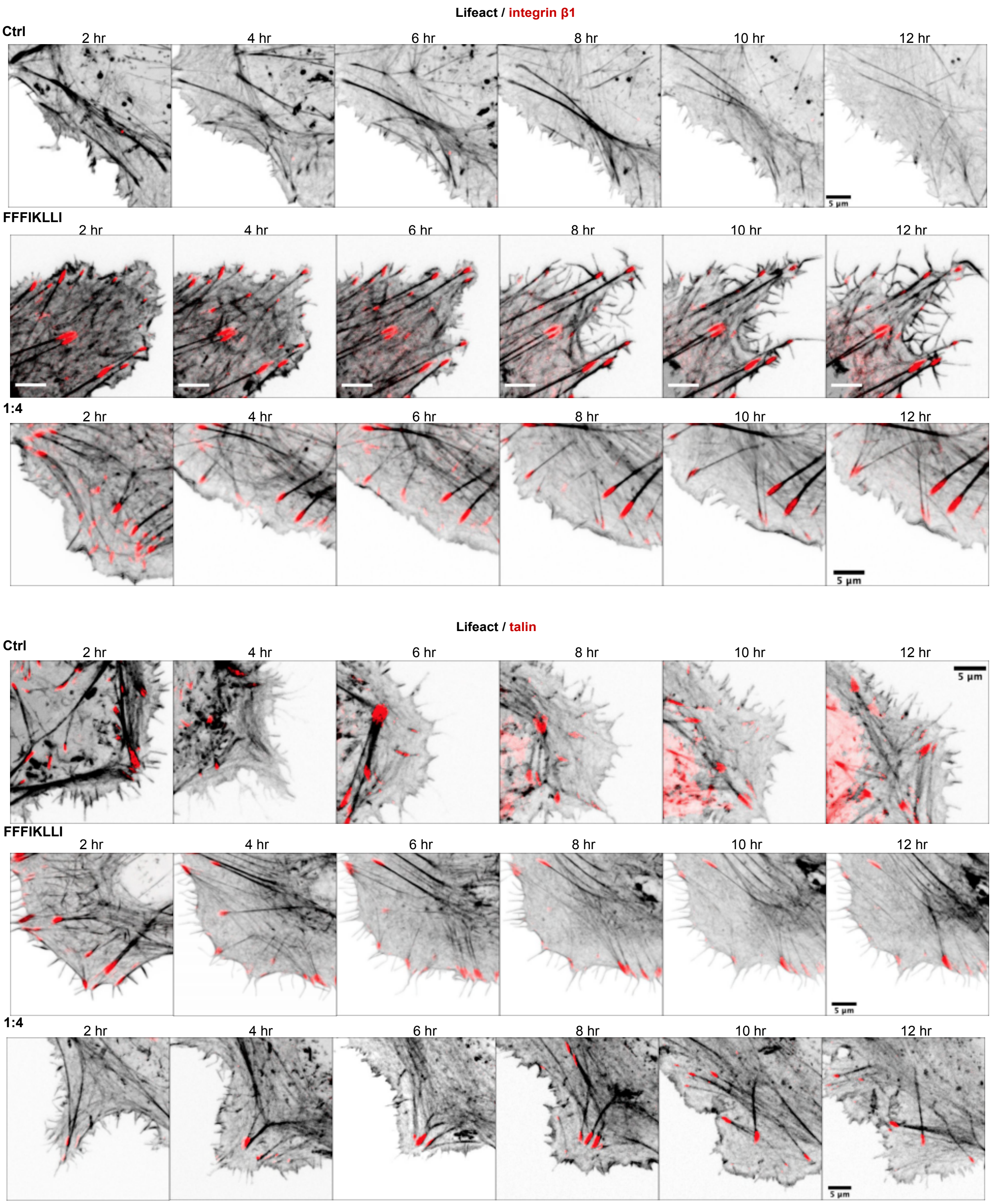

Supplementary Figure 26. The dynamics of actin cytoskeleton, integrin, and talin in HuH-7 cells.

Representative time-lapse images showing the dynamics of actin cytoskeleton (grey) and focal adhesion proteins(red) in HuH-7 cells co-transfected with mRuby-Lifeact-7 and mVenus-Integrin-

Beta1 or EGFP-talin upon the treatment of peptide assemblies for 12 hrs. Scale bars represent 5μm. Three independent experiments were performed.

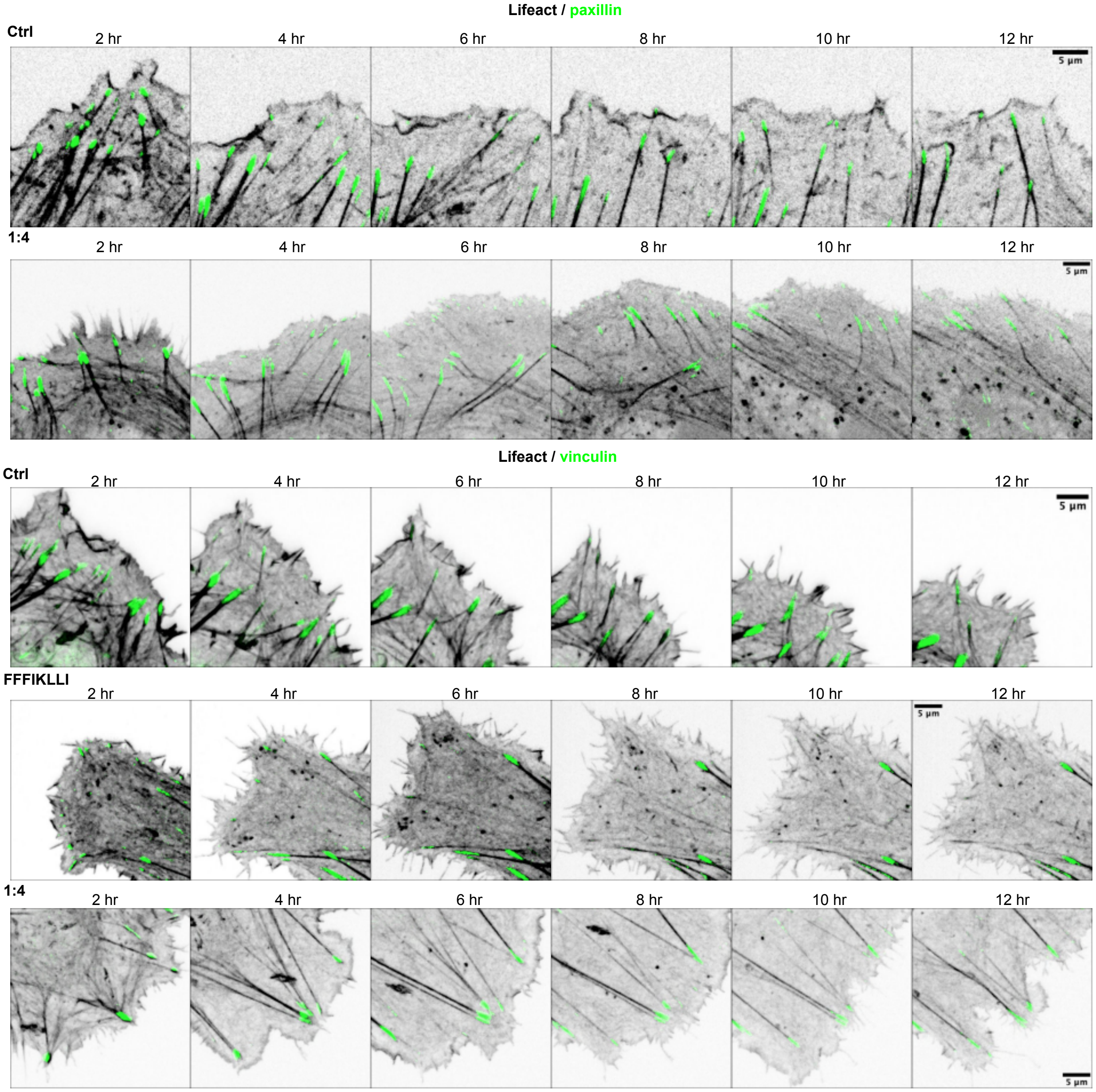

Supplementary Figure 27. The dynamics of actin cytoskeleton, paxillin, and vinculin in HuH-7 cells.

Representative time-lapse images showing the dynamics of actin cytoskeleton (grey) and focal adhesion proteins(green) in HuH-7 cells co-transfected with mRuby-Lifeact-7 and mGFP-paxillin or pEGFP-vinculin upon the treatment of peptide assemblies for 12 hrs. Scale bars represent 5μm. Three independent experiments were performed.

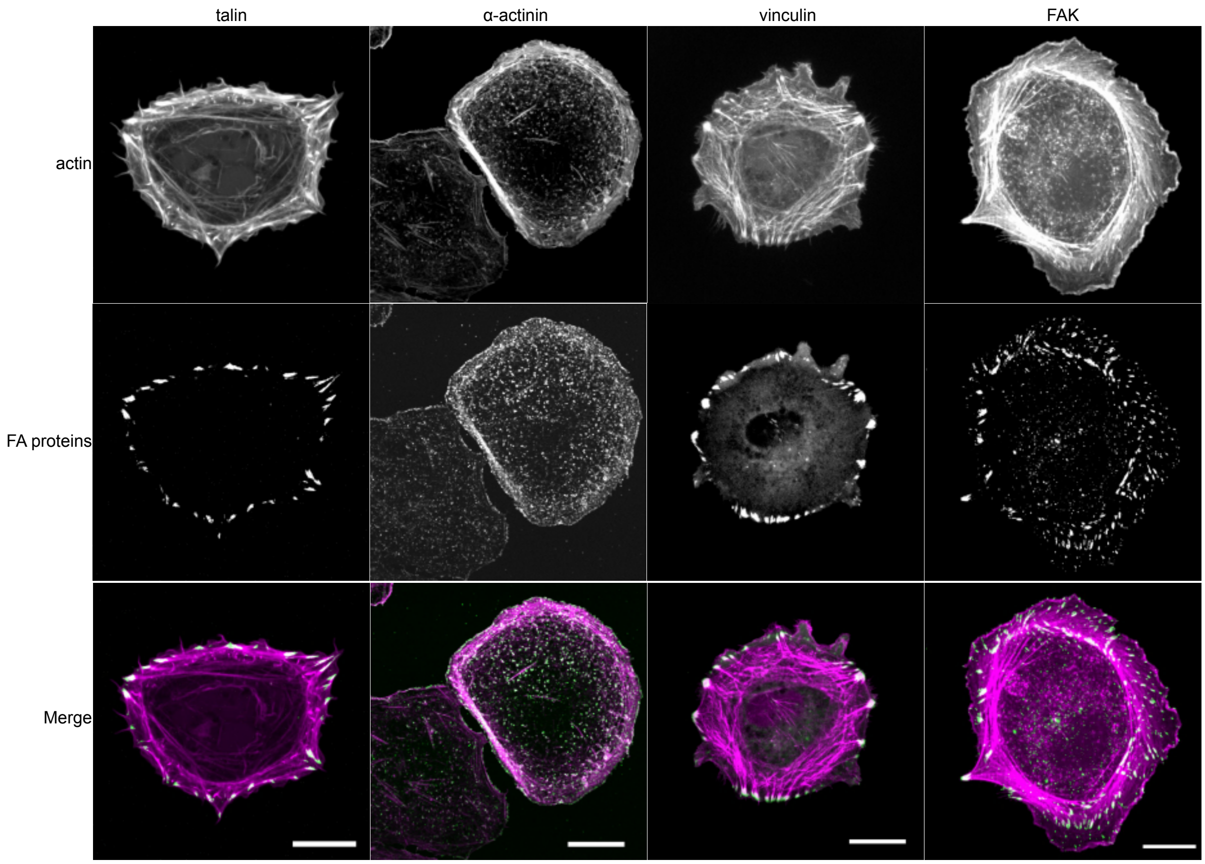

Supplementary Figure 28. Immunofluorescence of focal adhesion proteins co-stained with phalloidin in HuH-7 cells.

Representative images showing the immunofluorescence of focal adhesion (FA) proteins co-stained with phalloidin. HuH-7 cells were incubated without FFFIKLLI. The first column, phalloidin staining; the second column, immunofluorescence of FA proteins; the third row, merge images, green: FA proteins, magenta, phalloidin. Scale bars represent 20 μm. At least three independent experiments were performed.

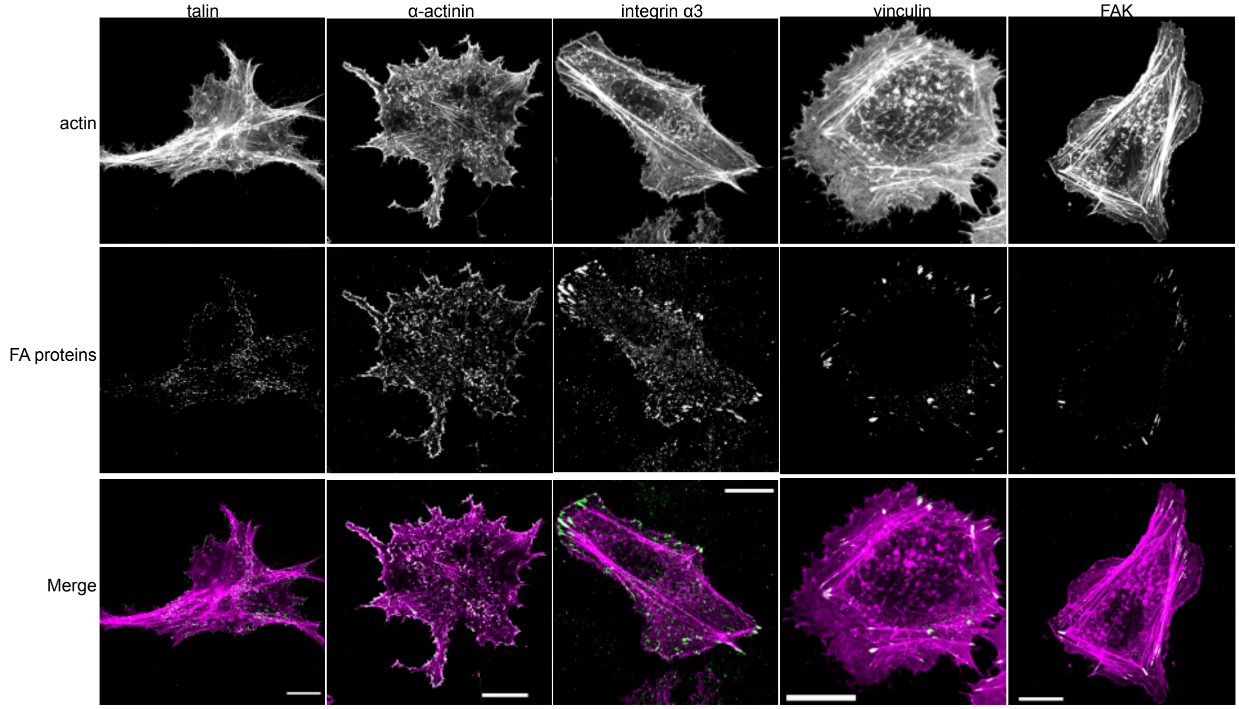

Supplementary Figure 29. Immunofluorescence of focal adhesion proteins co-stained with phalloidin in HuH-7 cells.

Representative images showing the immunofluorescence of focal adhesion (FA) proteins co-stain with phalloidin. HuH-7 cells were incubated with FFFIKLLI for 12 hr before fixed. The first column, phalloidin staining; the second column, immunofluorescence of FA proteins; the third row, merge images, green: FA proteins, magenta, phalloidin. Scale bar: 20 µm. At least three independent experiments were performed.

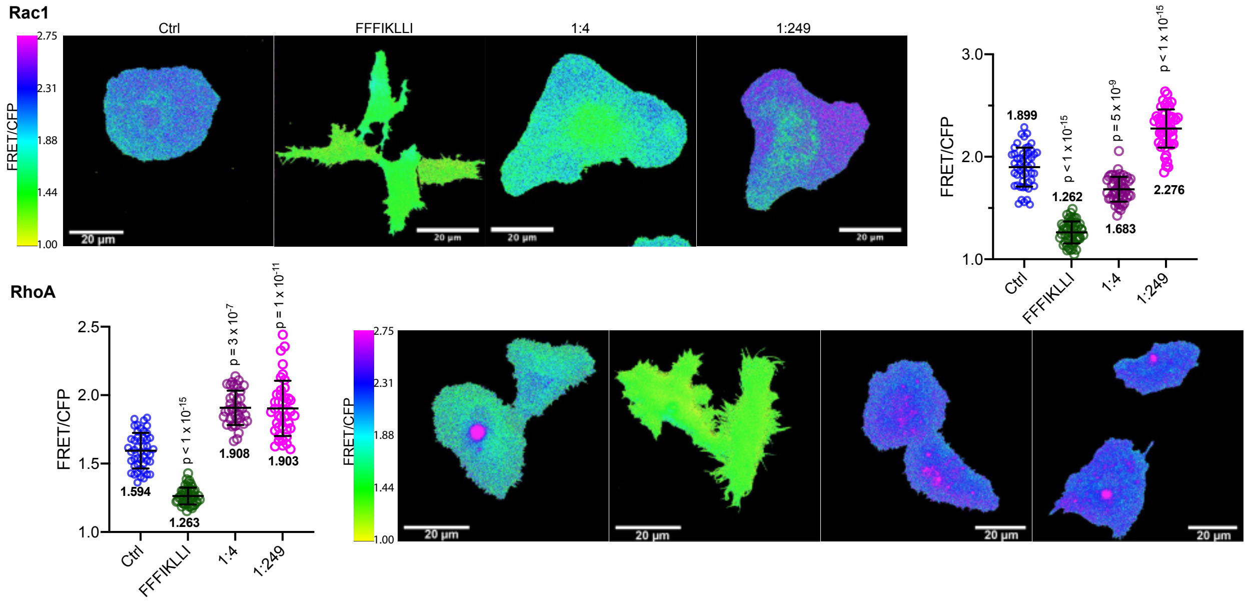

Supplementary Figure 30. Rac1 and RhoA activity in HuH-7 cells upon various treatments. Representative images and quantitative analysis of HuH-7 cells expressing RaichuEV-Rac1 or DORA-RhoA with or without the treatment of peptide assemblies for 12 hrs. The images were coded according to a pseudo color scale, which ranges from yellow to purple with an increase in FRET activity. Scale bars represent 20 µm. n=50 cells for each group. Kruskal-Wallis with Dunn's multiple comparisons test was used for analysis of the data. Data are presented as mean ± s.d.. Source numerical data are available in source data.

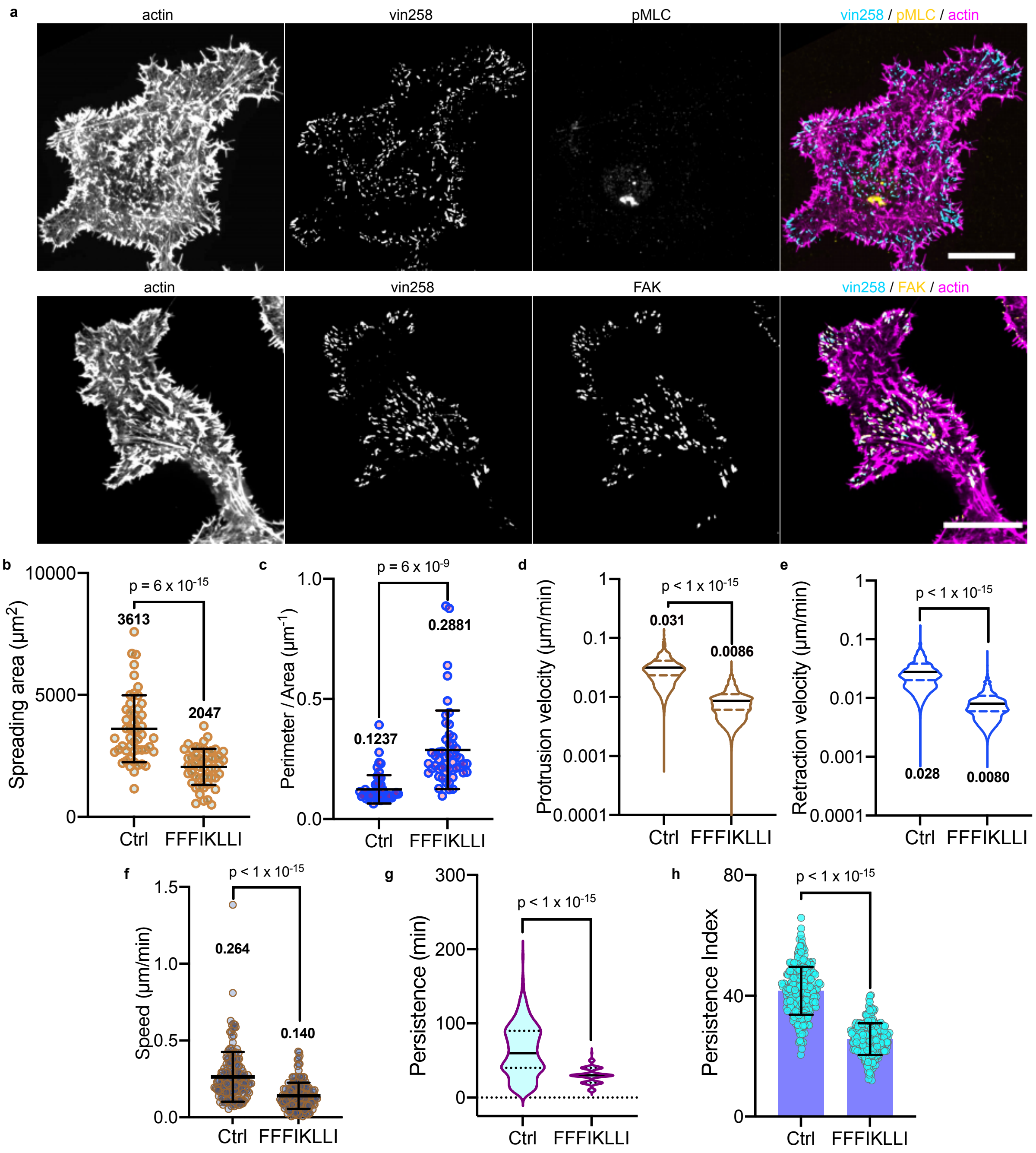

Supplementary Figure 31. Vin258 maintained the FAs on the periphery edge of HuH-7 cells. (**a**) Representative images showing the immunofluorescence of pMLC or FAK co-stain with phalloidin. HuH-7 cells were transfected with Pefgpc1/Gg Vcl 1-258 and treated with FFFIKLLI for 12 hr. Scale bars represent 20 µm. Three independent experiments were performed. (**b-c**) Spreading area and P/A ratio of vin258-transfected HuH-7 cells with or without treating with FFFIKLLI for 12 hr. Two-sided Mann-Whitney test was used for analysis of the data. Error bars represent standard deviation. n = 51 cells. Data are presented as mean ± s.d.. (**d-e**) Violin plot of all protrusion or retraction velocity values collected from each time point for transfected HuH-7

cells pretreated with or without FFFIKLLI for 12 hr. Two-sided Mann-Whitney test was used for analysis of the data. Median and quartiles were presented in the plot. n = 10 cells for each group. (**f-h**) The migration speed, directional persistence and persistence index of randomly selected migrating transfected HuH-7 cells. Two-sided Mann-Whitney test was used for analysis of the data. Data are presented as mean ± s.d . n= 169, 176 cells, respectively. Source numerical data are available in source data.

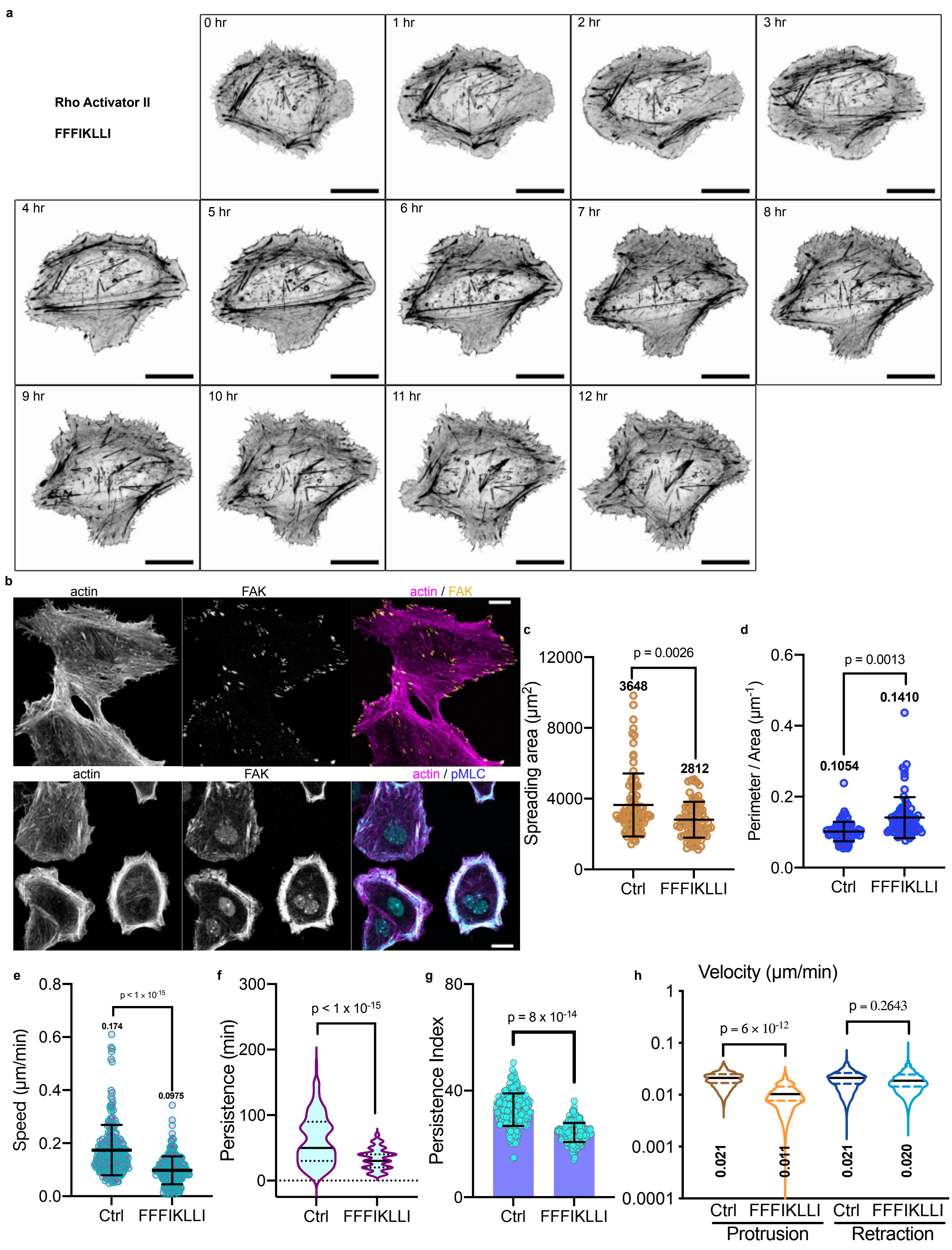
a
Rho Activator II
FFFIKLLI
0 hr
1 hr
2 hr
3 hr
4 hr
5 hr
6 hr
7 hr
8 hr
9 hr
10 hr
11 hr
12 hr
b
actin
FAK
actin / FAK
actin
FAK
actin / pMLC
c
p = 0.0026
3648
2812
Spreading area (μm²)
Ctrl
FFFIKLLI
d
p = 0.0013
0.1410
0.1054
Perimeter / Area (μm⁻¹)
Ctrl
FFFIKLLI
e
p < 1 x 10⁻¹⁵
0.174
0.0975
Speed (μm/min)
Ctrl
FFFIKLLI
f
p < 1 x 10⁻¹⁵
Persistence (min)
Ctrl
FFFIKLLI
g
p = 8 x 10⁻¹⁴
Persistence Index
Ctrl
FFFIKLLI
h
Velocity (μm/min)
p = 6 x 10⁻¹²
p = 0.2643
0.021
0.011
0.021
0.020
Ctrl
FFFIKLLI
Ctrl
FFFIKLLI
Protrusion
Retraction

Supplementary Figure 32. Rho activations reboot trailing tail retraction in HuH-7 cells.

(**a**) Representative images showing the time-lapse imaging of Rho-preactivated HuH-7 cells (mRuby-Lifeact-7 transfected) upon the treatment of FFFIKLLI. Scale bars represent 20 µm. Three independent experiments were performed. (**b**) Representative images showing the immunofluorescence of FAK and pMLC co-stain with phalloidin. Cells were preincubated with Rho Activator II and treated with FFFIKLLI for 12 hr. Scale bar represents 20 µm. Three independent experiments were performed. **(c, d).** Spreading area and P/A ratio of Rho-preactivated cells with or without treating with FFFIKLLI for 12 hr. Two-sided Mann-Whitney test was used for analysis of the data. Data are presented as mean ± s.d.. n = 85, 76 cells, respectively. (**e-g**) The migration speed, directional persistence and persistence index of randomly selected migrating Rho-preactivated HuH-7 cells. Two-sided Mann-Whitney test was used for analysis of the data. Data are presented as mean ± s.d.. n= 219, 221 cells, respectively. (**h**) Violin plot of all protrusion or retraction velocity values collected from each time point for Rho-activated HuH-7 cells and treated with or without FFFIKLLI for 12 hr. Two-sided Mann-Whitney test was used for analysis of the data. Median and quartiles were presented in the plot. n = 9, 12 cells respectively. Source numerical data are available in source data.

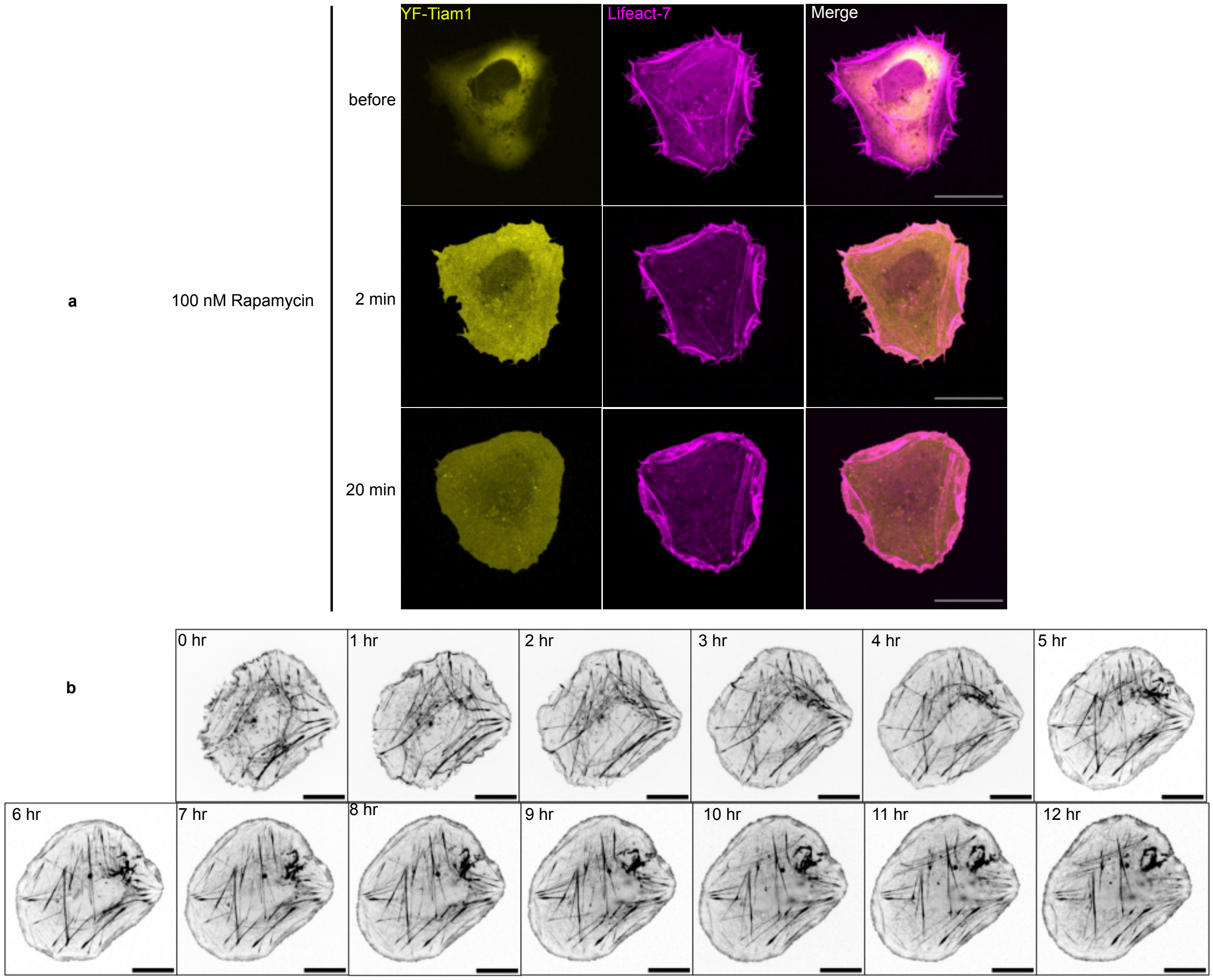

Supplementary Figure 33. Tiam1/Rac1 activation resume the cell protrusion.

(**a**) Representative imagines showing the lamellipodia stared to form within 2 minutes after Rac1 activation. Scale bars represent 20 µm. Three independent experiments were performed. (**b**) Representative time-lapse imaging of Rac1-activated HuH-7 cells (co-transfected with mRuby-Lifeact-7) upon the treatment of FFFIKLLI. Scale bars represent 20 µm. Three independent experiments were performed.

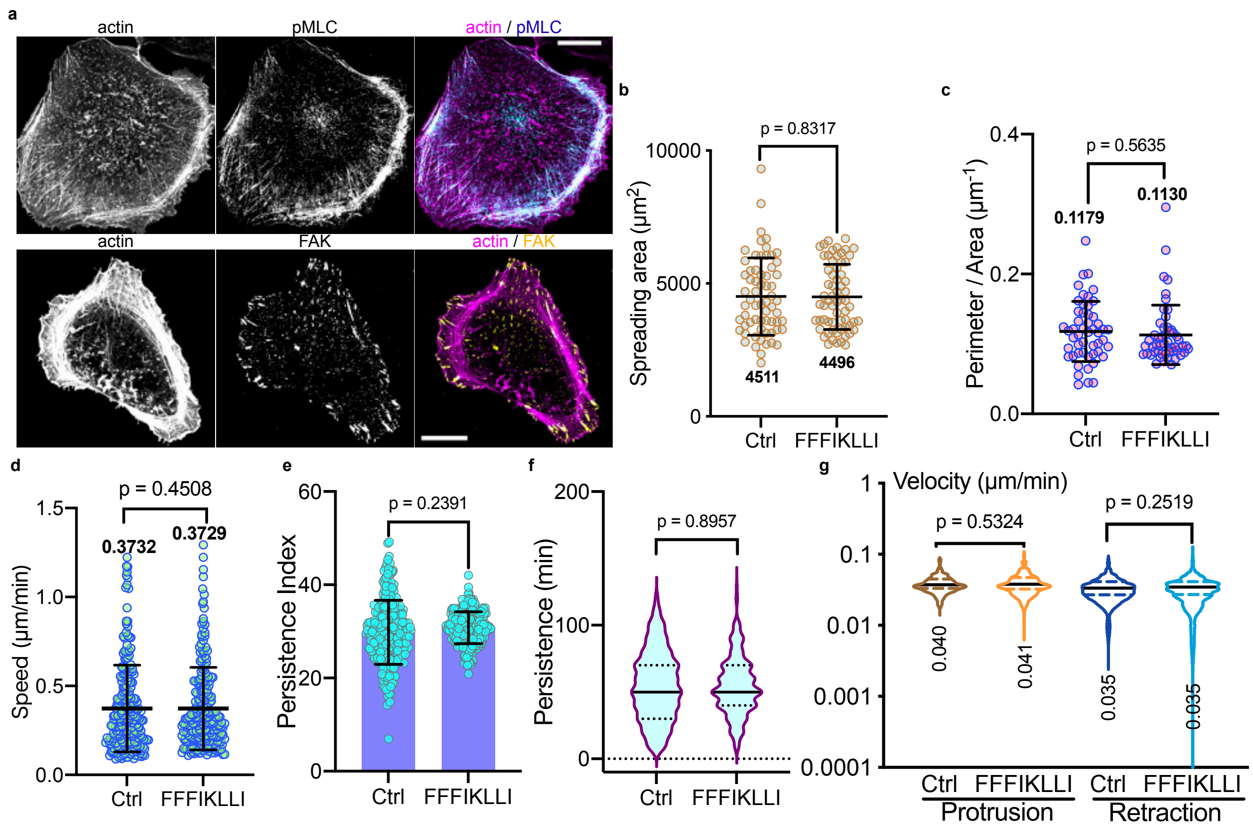

Supplementary Figure 34. Tiam1/Rac1 activation reboots the cell migration.

(**a**) Representative images showing the immunofluorescence of FAK and pMLC co-stain with phalloidin. Tiam1/Rac1 was preactivated right before starting the 12 hr treatment of FFFIKLLI. Scale bars represent 20 μm. At least three independent experiments were performed. (**b, c**) Spreading area and P/A ratio of Tiam1/Rac1-preactivated cells with or without treating with FFFIKLLI for 12 hr. Two-sided Mann-Whitney test was used for analysis of the data. Error bars represent standard deviation. n = 64 cells. Data are presented as mean ± s.d. (**d-f**) The migration speed, directional persistence time and persistence index of randomly selected migrating Tiam1/Rac1-activated HuH-7 cells. Two-sided Mann-Whitney test was used for analysis of the data. Data are presented as mean ± s.d.. n= 230, 232 cells, respectively. (**g**) Violin plot of all protrusion or retraction velocity values collected from each time point for Tiam1/Rac1-activated HuH-7 cells and treated with or without FFFIKLLI for 12 hr. Two-sided Mann-Whitney test was used for analysis of the data. Median and quartiles were presented in the plot. n = 7, 8 cells respectively. Source numerical data are available in source data.

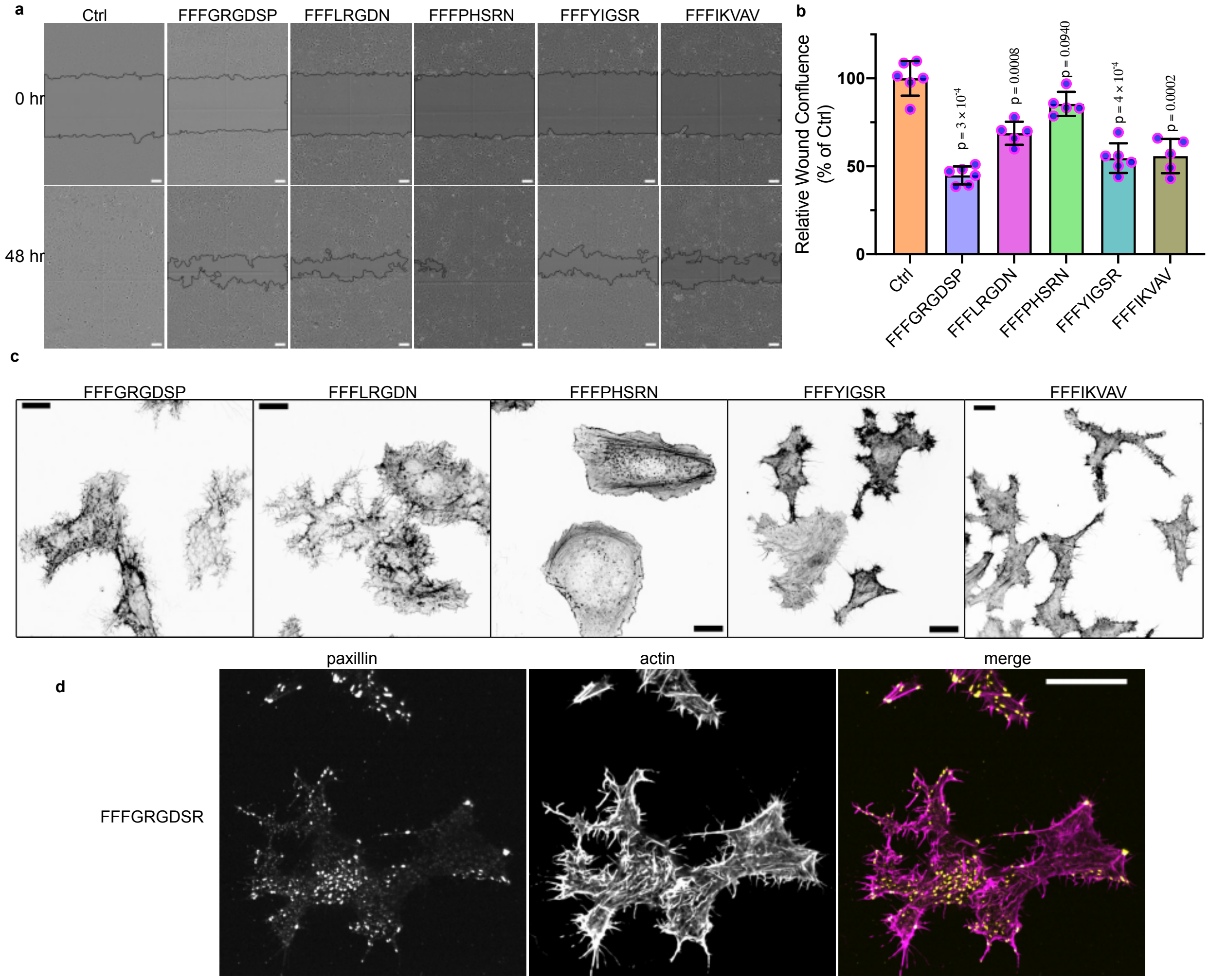

Supplementary Figure 35. The other assembling ligands affect cell migration.

(**a-b**) Representative images and 48hr-Wound healing rate of HuH-7 cells upon the treatment of 200 µM assembling ligands. Scale bars represent 100 µm. At least five independent experiments were performed. Kruskal-Wallis with Dunn's multiple comparisons test was used for analysis of the data. (**c**) Representative images showing the phalloidin staining of HuH-7 cells incubated with 200 µM assembling ligands for 12 hr. Scale bars represent 20 µm. At least three independent experiments were performed. (**d**) Representative images showing immunofluorescence of paxillin co-stain with phalloidin. HuH-7 cells were incubated with 200 µM assembling ligands for 12 hr. Scale bar represents 20 µm. Three independent experiments were performed. Source numerical data are available in source data.

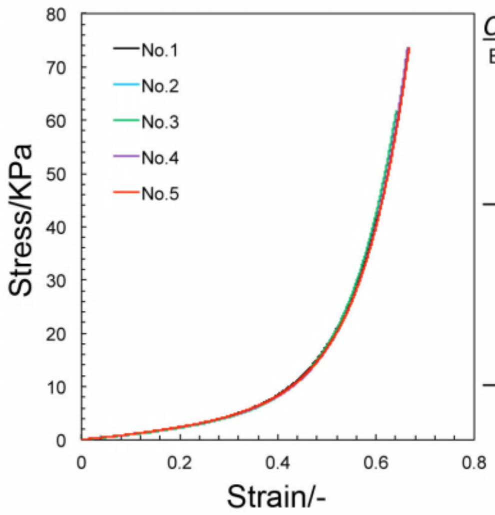

Compression test condition
EZ test (Shimadzu)

Thickness: ca 5 mm
Width: ca 9 mm, Length: ca 9 mm
Rate: 0.5 mm/min

No.	Rate / mm/min	Thickness / mm	Width /mm	Length / mm	Young's modulus / KPa
1	0.5	5.0	8.5	8.5	12.3
2	0.5	5.0	8.5	8.5	11.7
3	0.5	5.0	9.0	9.0	11.9
4	0.5	5.0	8.0	8.5	12.1
5	0.5	5.0	8.0	8.5	12.5
				Ave	12.1
				SD	0.29

Supplementary Figure 36. The stiffness of the PDMS substrate characterized using compression test.

Lot No.	:P210114-YW125651		
Column	:Diamonsil C18, 4.6*250mm, 5um		
Solvent A	:0.1%Trifluoroacetic in 100% Acetonitrile		
Solvent B	:0.1%Trifluoroacetic in 100% Water		
Gradient	:	A	B
	0.0min	32%	68%
	25.0min	57%	43%
	25.1min	100%	0%
	30.0min	Stop	
Flow rate	:1.0ml/min		
Wavelength	:220nm		
Volume	:20ul		

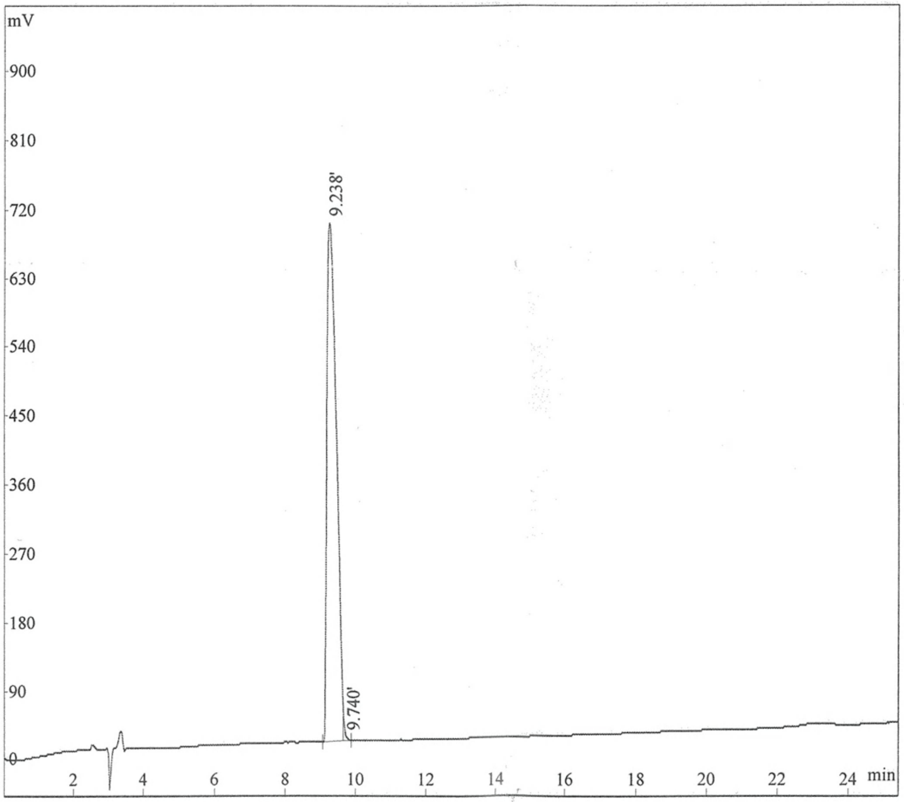

Rank	Time	Quantity	Area	Height
1	9.238	99.51	11982890	679242
2	9.740	0.4857	58488	3542
Total		100	12041378	682784

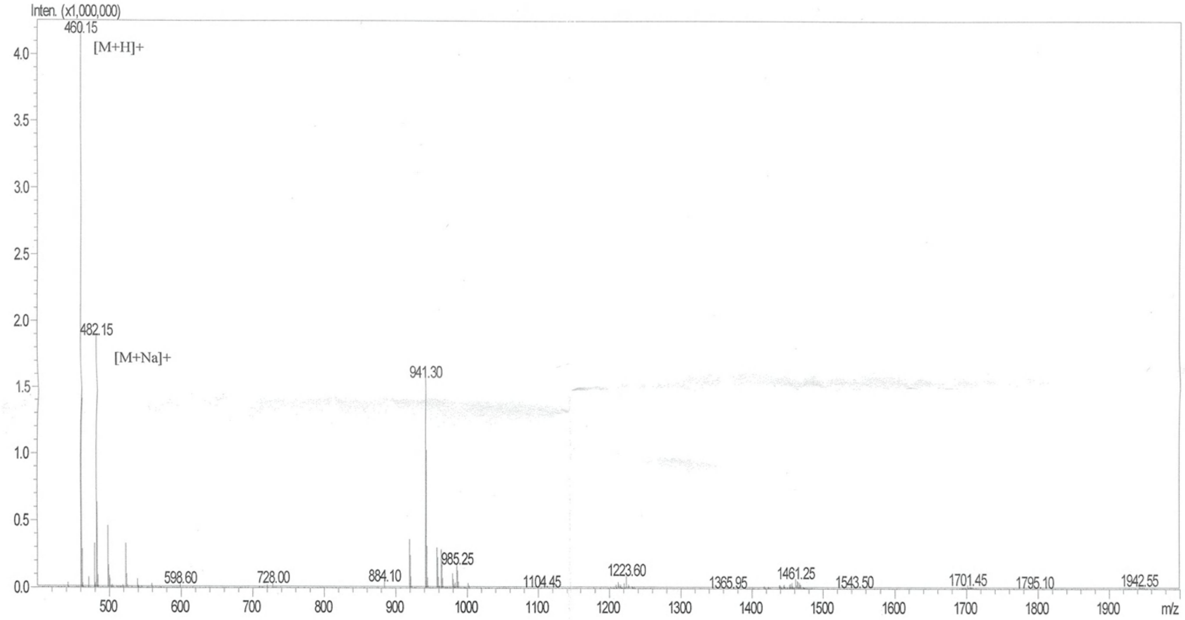

Mw	: 459.57		
Lot No.	: P210114-YW125651		
Probe	:ESI	Probe bias	:+4.5kv
Nebulizer Gas Floe	:1.5L/min	Detector	:1.2kv
CDL	:-20.0v	T.Flow	:0.2ml/min
CDL Temp	:250℃	B.conc	:50%H2O/50%ACN
Block Temp	:400℃		

Supplementary Figure 37. LC-MS spectra of FFF.

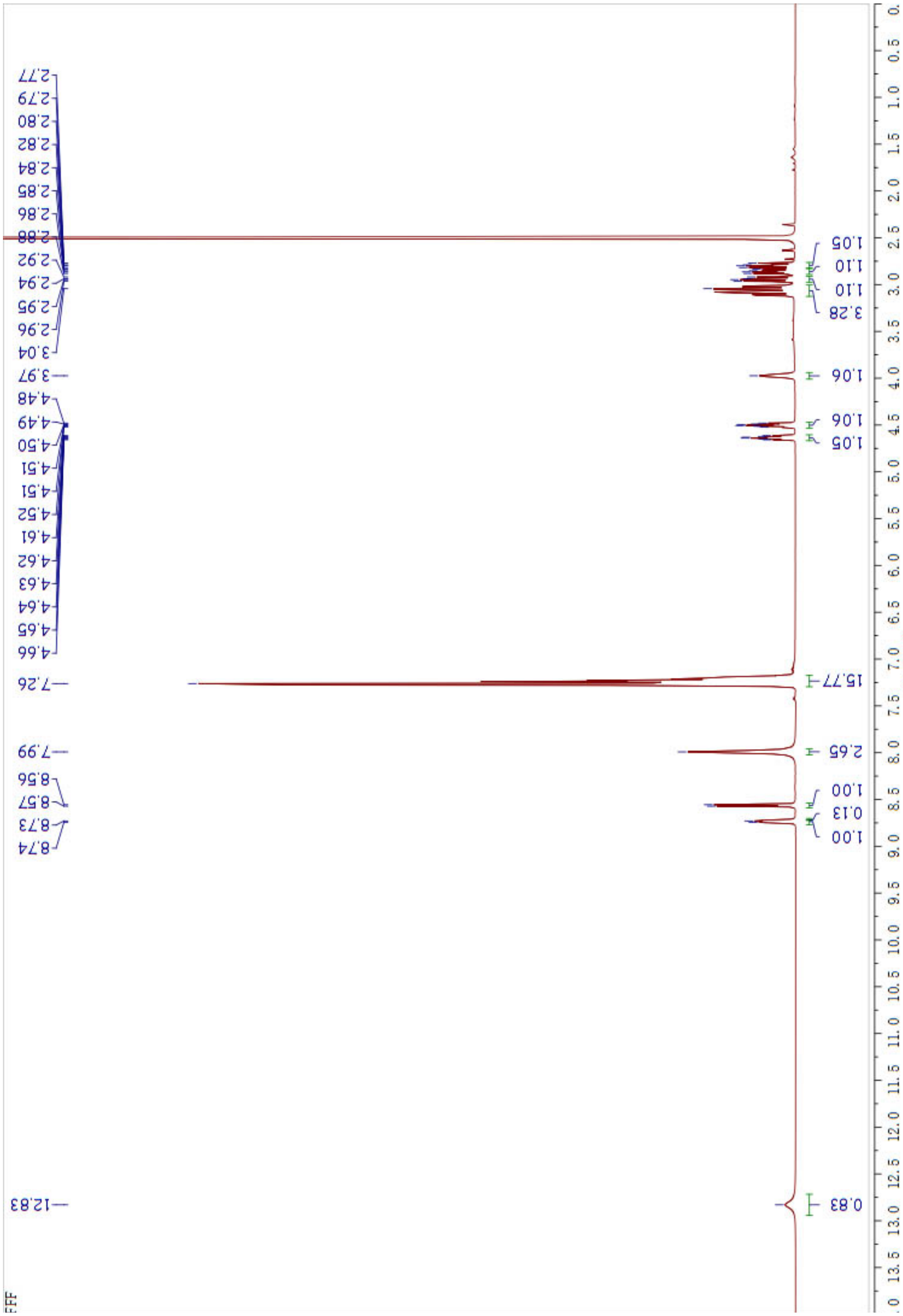

Supplementary Figure 38. ^{1}**H NMR of FFF.**

Lot NO :P190510-YS725631

Number :0200049

Column :250*4.6mm,Kromasil-C18-5um

Solvent A:0.1%TFA in 100%water

Solvent B:0.1%TFA in 100%acetonitrile

Gradient :		A	B
	0.1min	68%	32%
	25.0min	43%	57%
	25.1min	0%	100%
	30.0min	stop	

Flow rate:1.0ml/min

Wavelength(nm):220

Volume :10ul

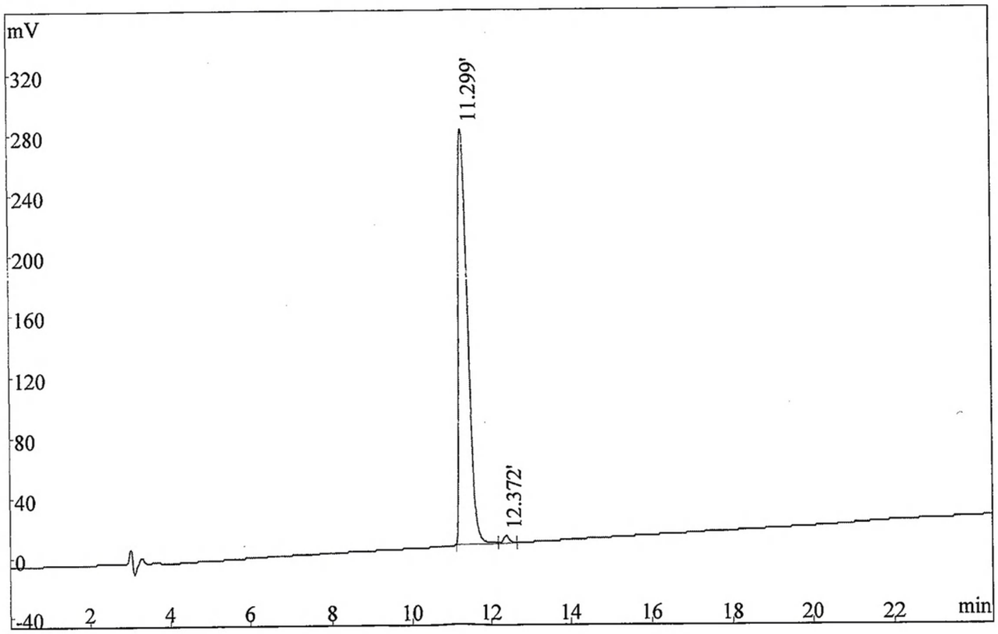

Rank	Time	Conc.	Area	Height
1	11. 299	98. 94	4131588	275311
2	12. 372	1. 062	44343	4677
Total		100	4175931	279988

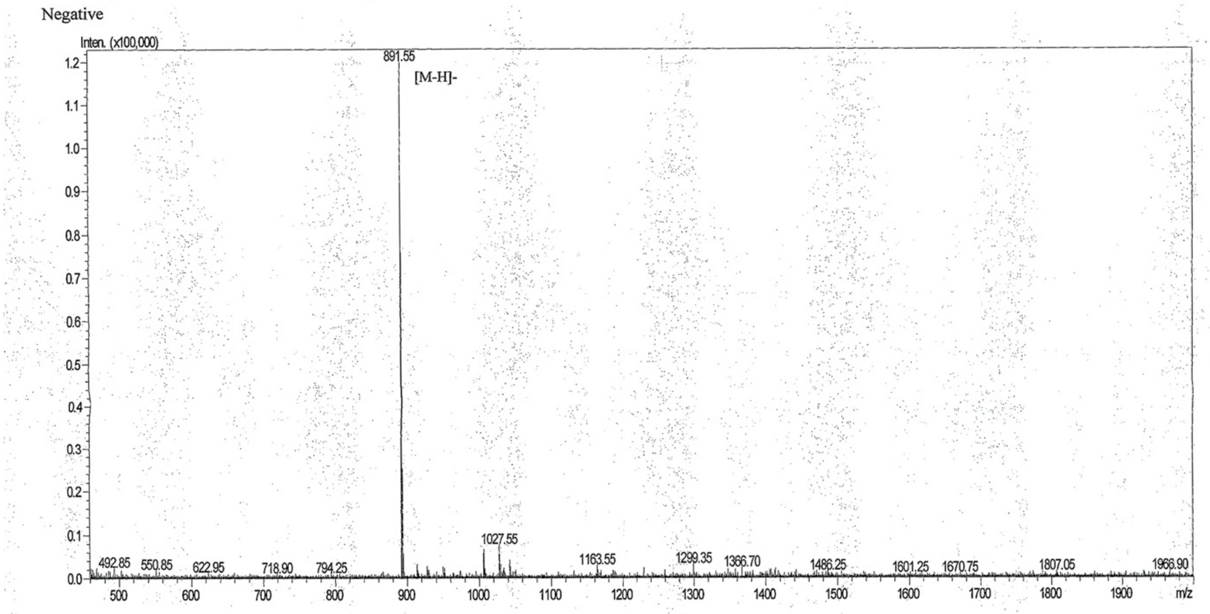

Injection Volume : 1

Sample Name : FI-7

Mw : 893.17

Lot No. : P190510-YS725631

Probe :ESI Probe bias :+4.5kv

Nebulizer Gas Floe :1.5L/min Detector :1.2kv

CDL :-20.0v T.Flow :0.2ml/min

CDL Temp :250℃ B.conc :50%H2O/50%ACN

Block Temp :400℃

Supplementary Figure 39. LC-MS spectra of FFIKLLI.

The purity of the peptide is 98.94%.

Lot No.	:P210114-YW687883
Column	:Diamonsil C18, 4.6*250mm, 5um
Solvent A	:0.1%Trifluoroacetic in 100% Acetonitrile
Solvent B	:0.1%Trifluoroacetic in 100% Water

Gradient		A	B
	0.0min	40%	60%
	25.0min	65%	35%
	25.1min	100%	0%
	30.0min	Stop	

Flow rate	:1.0ml/min
Wavelength	:220nm
Volume	:20ul

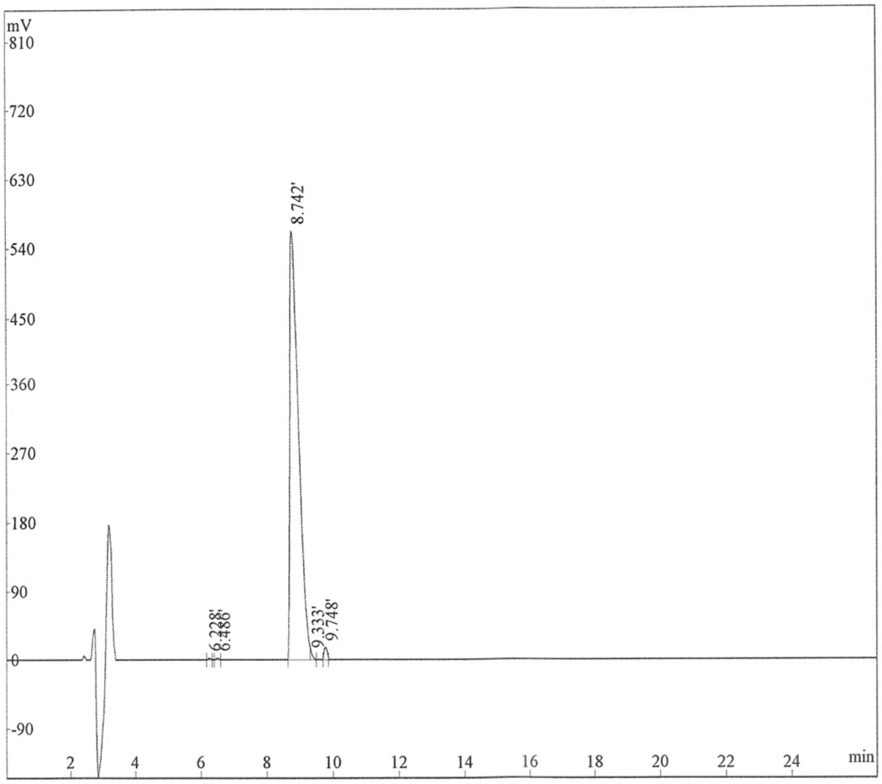

Rank	Time	Quantity	Area	Height
1	6.228	0.1375	13406	2662
2	6.486	0.1991	19405	3339
3	8.742	98.32	9583033	560515
4	9.333	0.4022	39207	6733
5	9.748	0.943	91913	15890
Total		100	9746964	589139

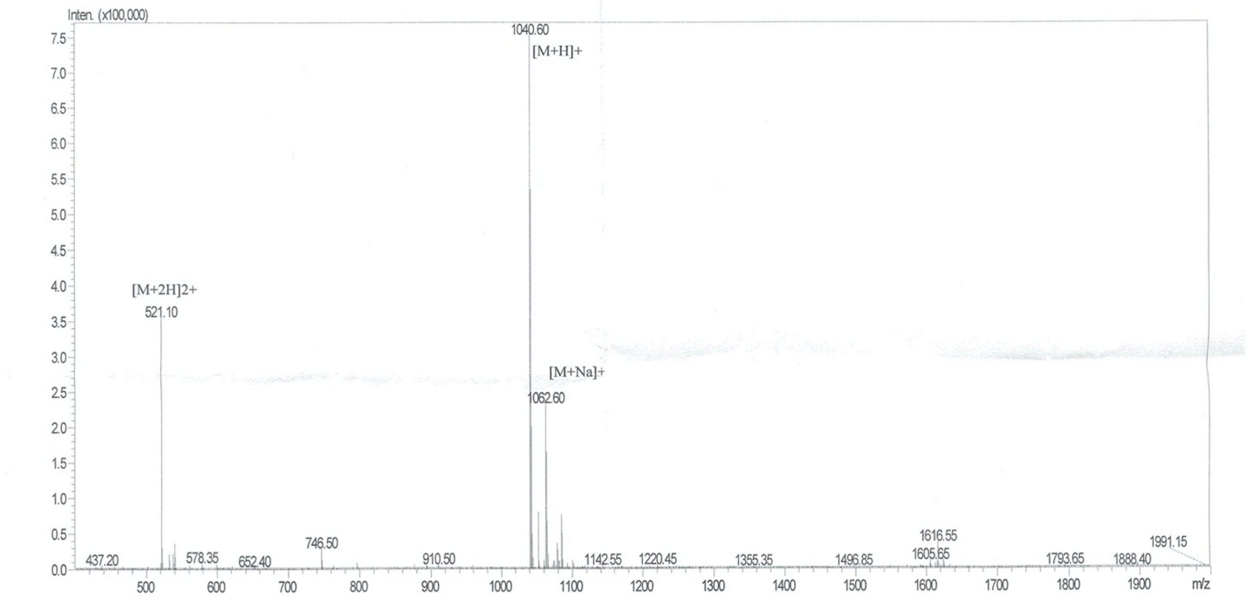

Mw	: 1040.44		
Lot No.	: P210114-YW687883		
Probe	:ESI	Probe bias	:+4.5kv
Nebulizer Gas Floe	:1.5L/min	Detector	:1.2kv
CDL	:-20.0v	T.Flow	:0.2ml/min
CDL Temp	:250℃	B.conc	:50%H2O/50%ACN
Block Temp	:400℃		

Supplementary Figure 40. LC-MS spectra of FFFIKLLI.

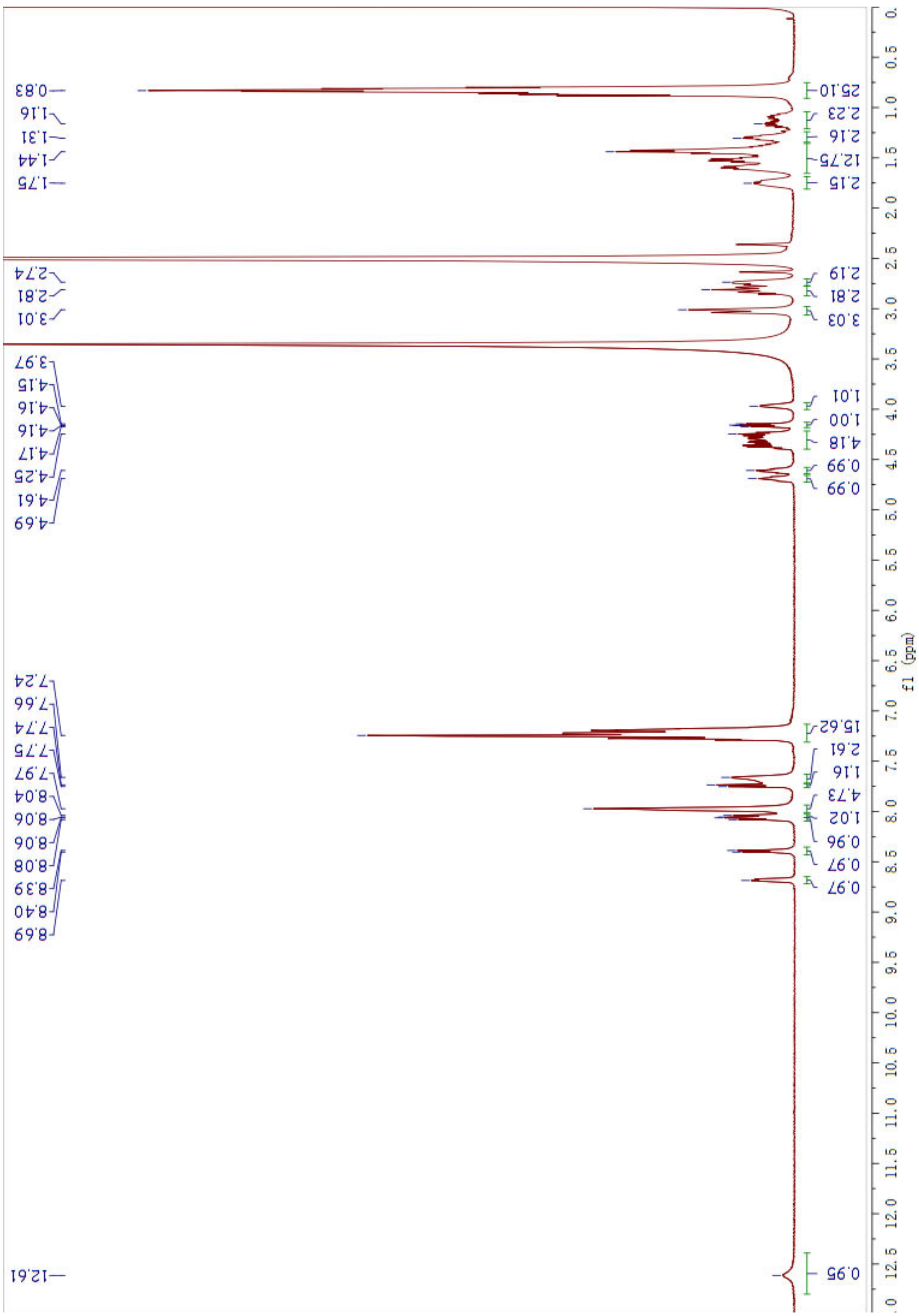

Supplementary Figure 41. ^{1}**H NMR of FFFIKLLI.**

Lot No.	:P191203-YS766468
Column	:Diamonsil C18, 4.6*250mm, 5um
Solvent A	:0.1%Trifluoroacetic in 100% Acetonitrile
Solvent B	:0.1%Trifluoroacetic in 100% Water

Gradient	:	A	B
	0.0min	35%	65%
	25.0min	60%	40%
	25.1min	100%	0%
	30.0min	Stop	

Flow rate	:1.0ml/min
Wavelength	:220nm
Volume	:20ul

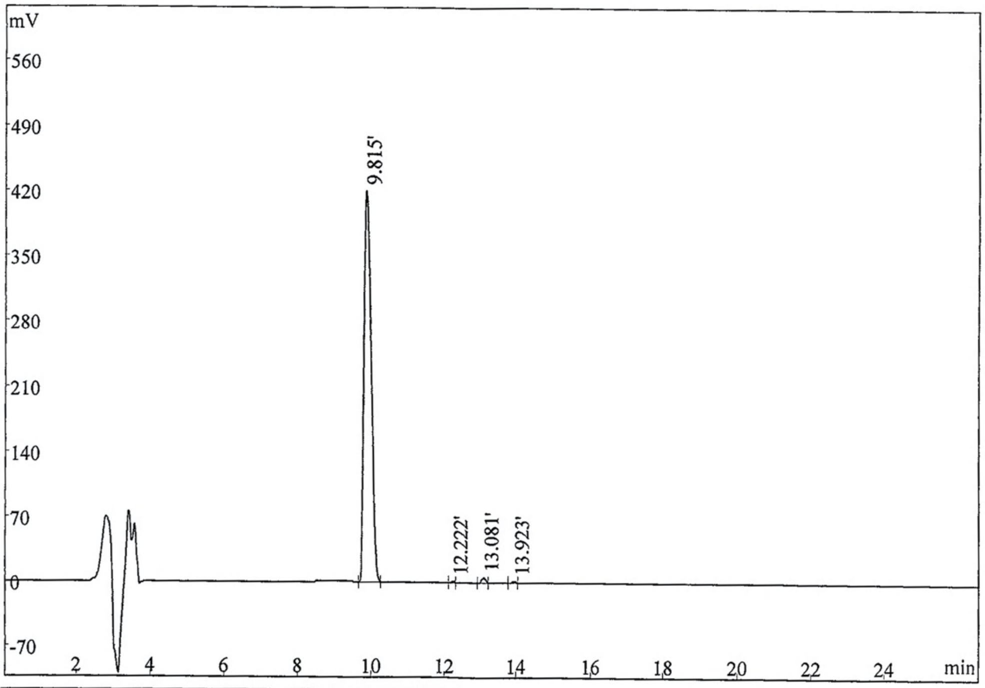

Rank	Time	Quantity	Area	Height
1	9.815	98.72	5120495	418503
2	12.222	0.2486	12896	1939
3	13.081	0.7815	40537	5163
4	13.923	0.2506	12999	1792
Total		100	5186927	427397

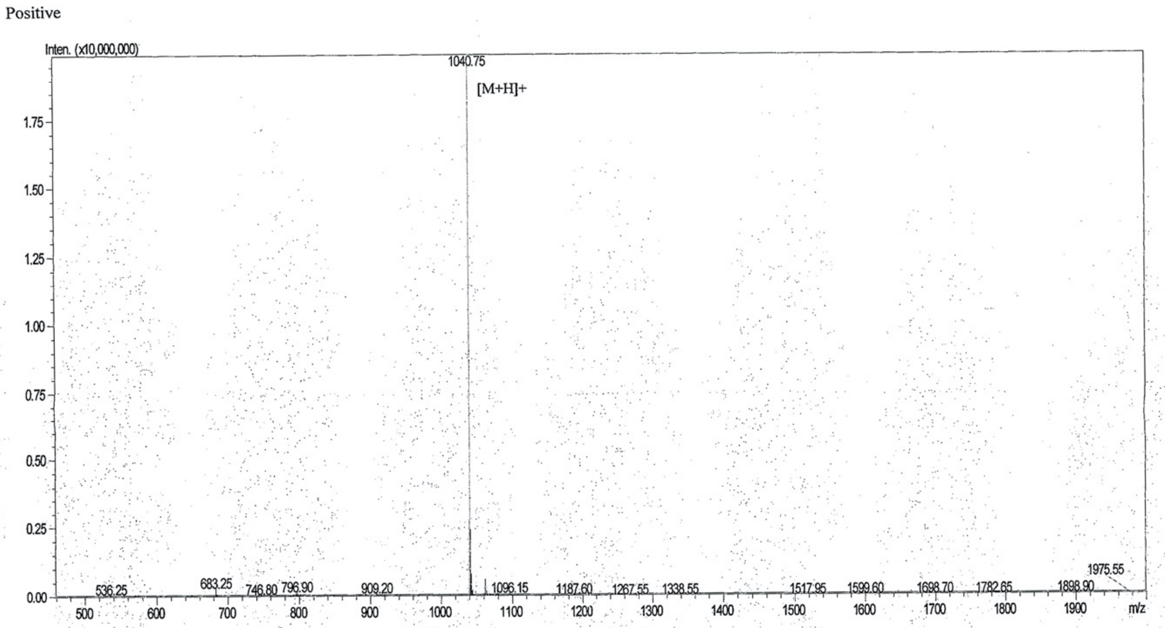

Mw : 1040.34
Lot No. : P191203-YS766468

Probe	:ESI	Probe bias	:+4.5kv
Nebulizer Gas Floe	:1.5L/min	Detector	:1.2kv
CDL	:-20.0v	T.Flow	:0.2ml/min
CDL Temp	:250℃	B.conc	:50%H2O/50%ACN
Block Temp	:400℃		

Supplementary Figure 42. LC-MS spectra of FFFKLIIL.

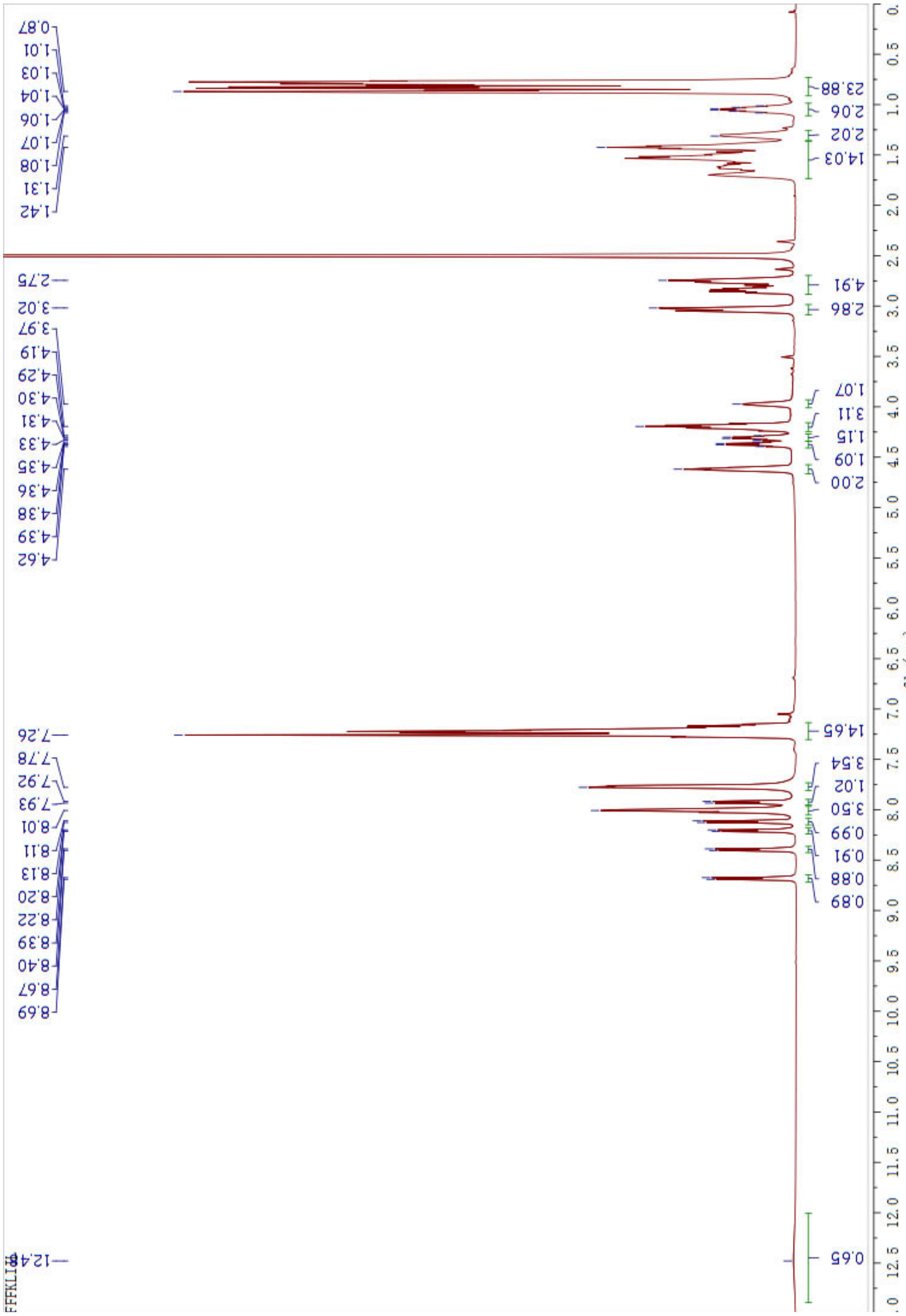

Supplementary Figure 43. ^{1}H NMR of FFFKLIIL.

Lot No ;P201123-YW848469

Column :4.6*250mm, kromasil C18-5

Solvent A :0.1%Trifluoroacetic in 100% Acetonitrile

Solvent B :0.1%Trifluoroacetic in 100% Water

Gradient :

	A	B
0.01min	25%	75%
25min	50%	50%
25.1min	100%	0%
30min	Stop	

Flow rate :1.0ml/min

Wavelength :220nm

Volume:5ul

File opened: F:848469-25-50.hw, where

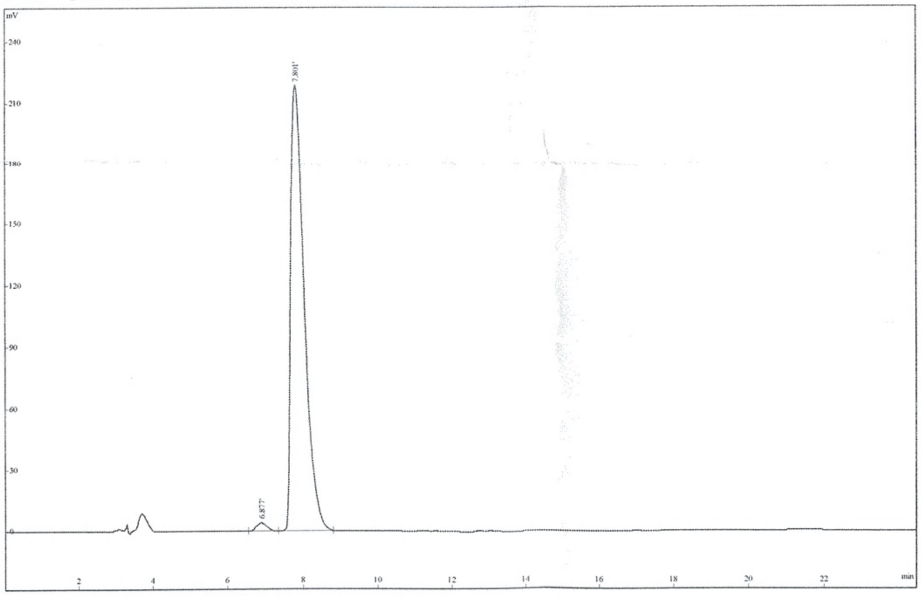

Rank	Time	Conc.	Area	Height
1	6.877	1.467	81003	4093
2	7.801	98.54	5441318	218925
Total		100	5522321	223018

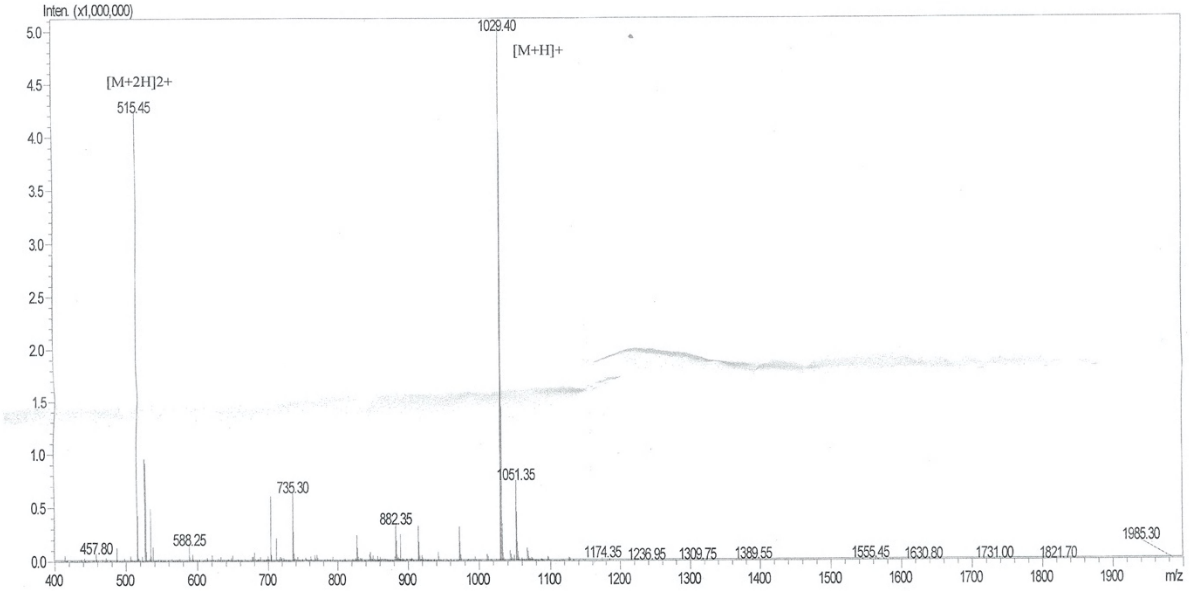

Mw	: 1029.20		
Lot No.	: P201123-YW848469		
Probe	:ESI	Probe bias	:+4.5kv
Nebulizer Gas Floe	:1.5L/min	Detector	:1.2kv
CDL	:-20.0v	T.Flow	:0.2ml/min
CDL Temp	:250℃	B.conc	:50%H2O/50%ACN
Block Temp	:400℃		

Supplementary Figure 44. LC-MS spectra of FFFGRGDSP.

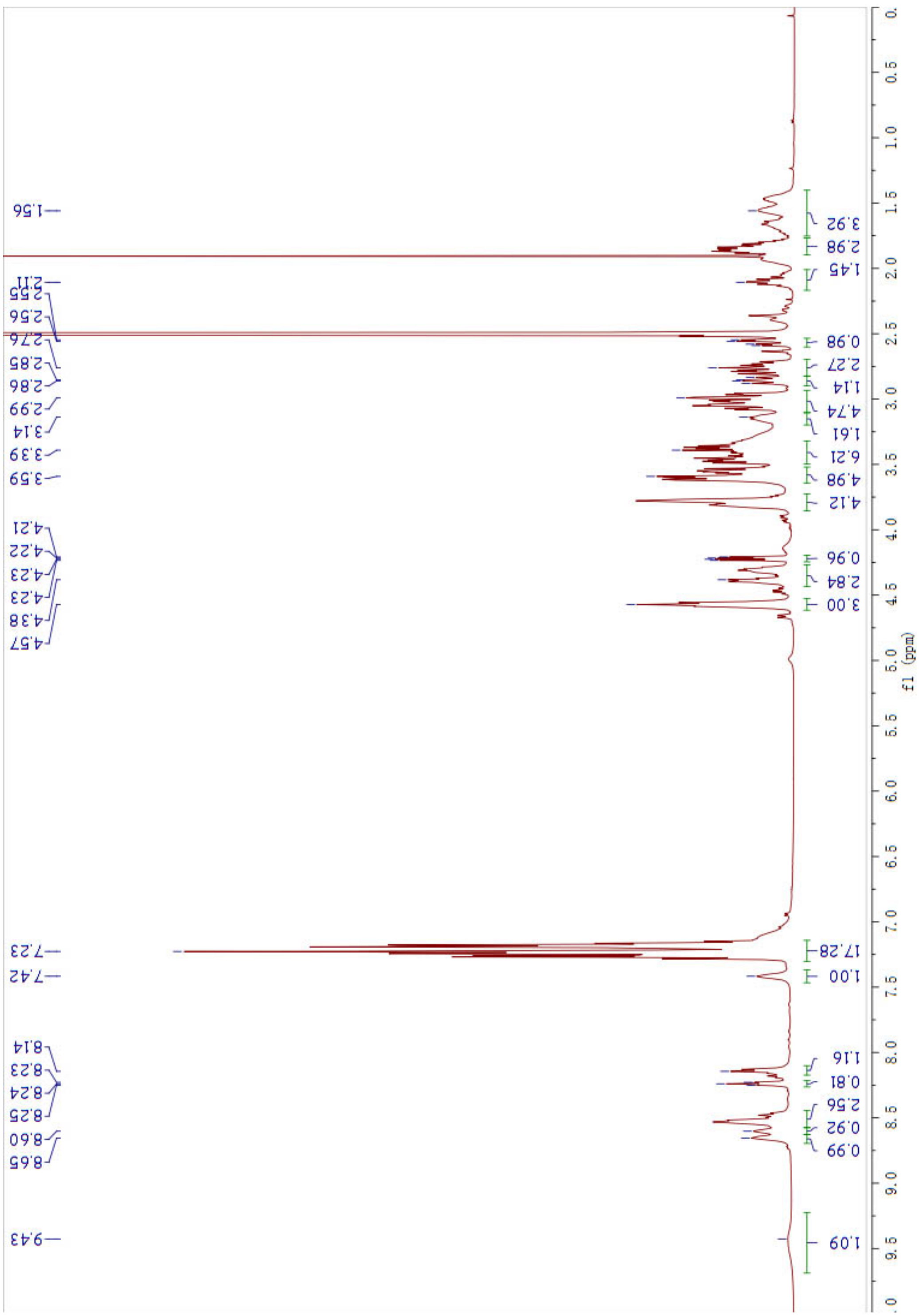

Supplementary Figure 45. ^{1}**H NMR of FFFGRGDSP.**

Lot No. : P201013-YW837788
Column : 4.6*250mm, 5um, 100A, Agela
Solvent A : 0.1% trifluoroacetic in 100% acetonitrile
Solvent B : 0.1% trifluoroacetic in 100% water

Gradient		A	B
	0.01min	26%	74%
	25min	51%	49%
	25.1min	100%	0%
	30min	STOP	

Flow rate : 1.0ml/min
Wavelength : 220nm
Volume :
10ul

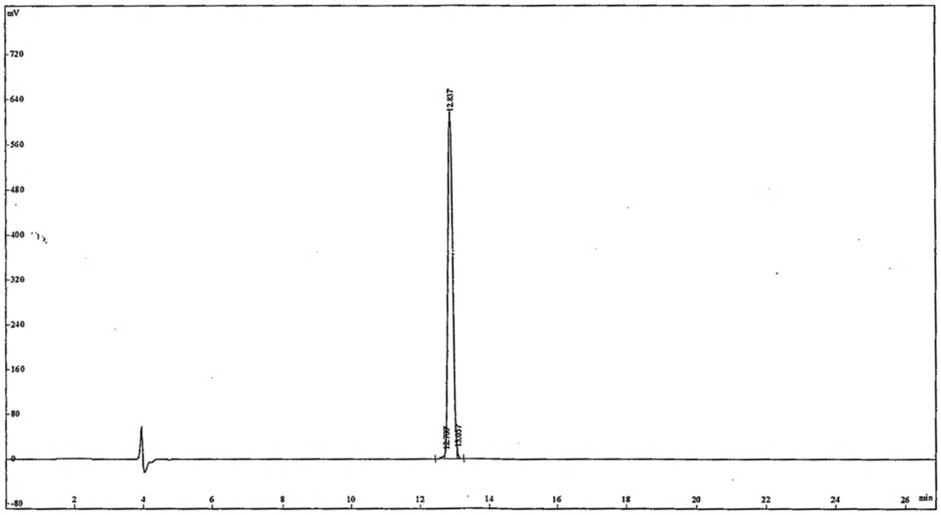

Peak No.	Ret Time	Conc	Area	Height
1	12.700	0.9246	57443	15317
2	12.837	98.73	6133771	623094
3	13.057	0.3464	21519	10059
Total		100	6212733	648470

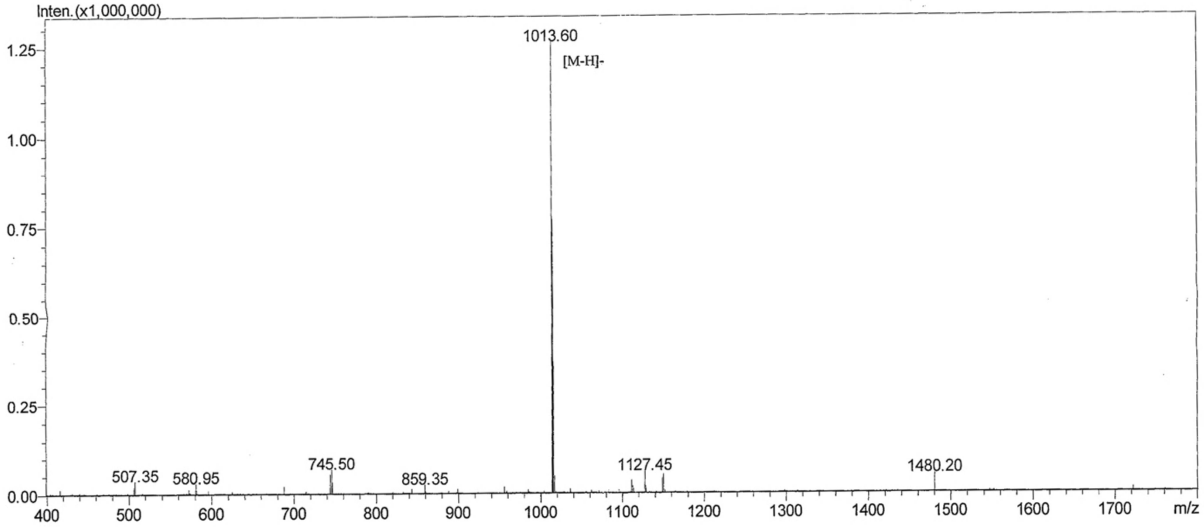

M.W.:	1015.21		
Lot. No.:	P201013-YW837788		
Instrument	SHIMADZU LCMS-2010EV		
Probe:	ESI	Probe Bias:	+4.5kv
Nebulizer Gas Flow:	1.5L/min	Detector:	1.5kv
CDL:	-20.0v	T. Flow:	0.2ml/min
CDL Temp.:	250 °C	B. Conc.:	50%H2O/50%ACN
Block Temp.:	200 °C		

Supplementary Figure 46. LC-MS spectra of FFFLRGDN.

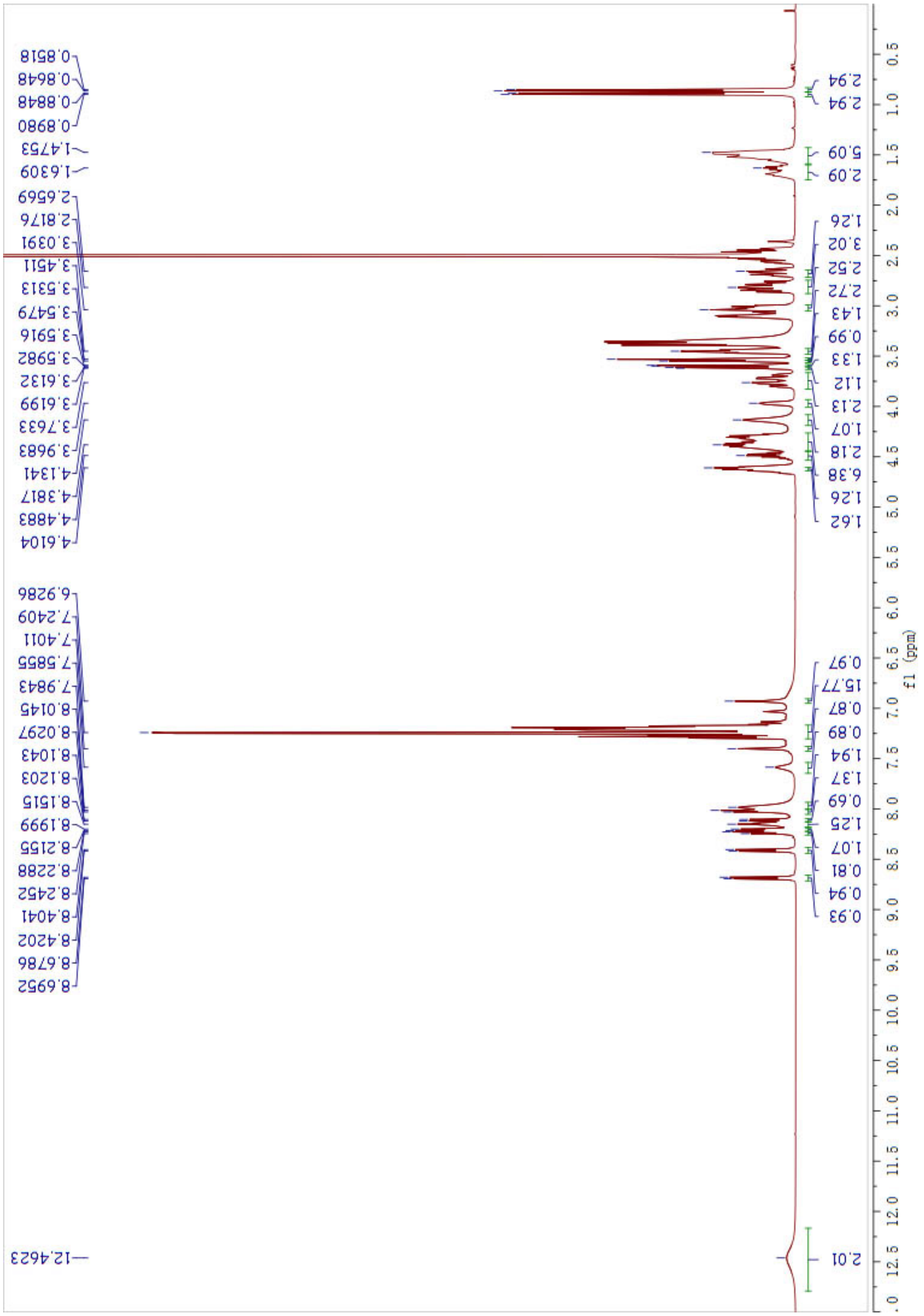

Supplementary Figure 47. ^{1}H NMR of FFFLRGDN.

Lot No　:P190510-YS725632
Number　:0200049
Column　:250*4.6mm, Kromasil-C18-5um
Solvent A:0.1%TFA in 100%water
Solvent B:0.1%TFA in 100%acetonitrile

Gradient :	A	B
0.1min	72%	28%
25.0min	47%	53%
25.1min	0%	100%
30.0min	stop	

Flow rate:1.0ml/min
Wavelength(nm):220
Volume:5ul

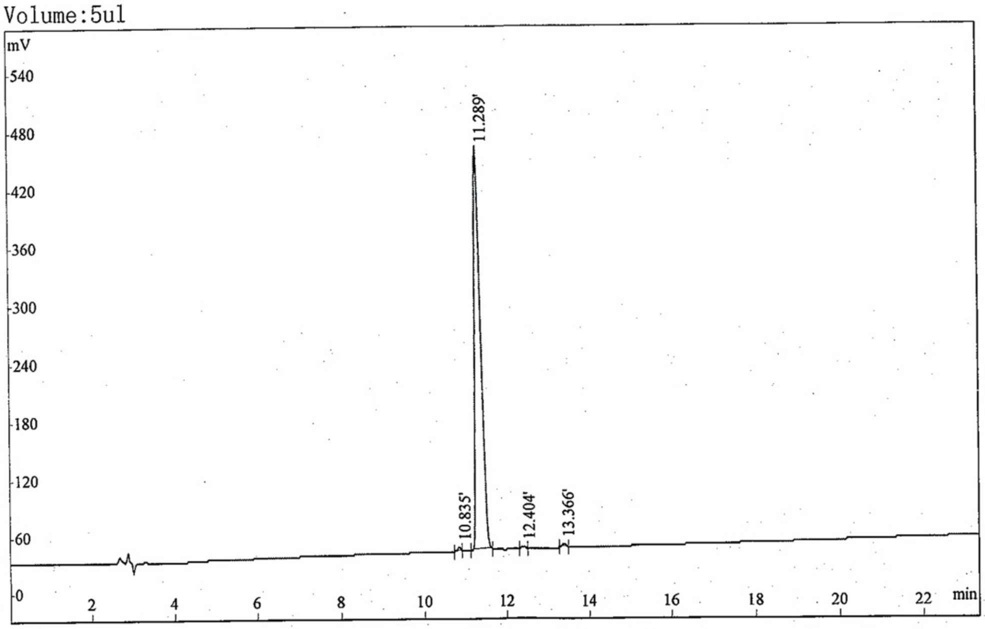

Rank	Time	Conc.	Area	Height
1	10.835	0.4214	18034	3546
2	11.289	98.7	4224327	421554
3	12.404	0.3394	14527	2434
4	13.366	0.5327	22798	3146
Total		100	4279686	430680

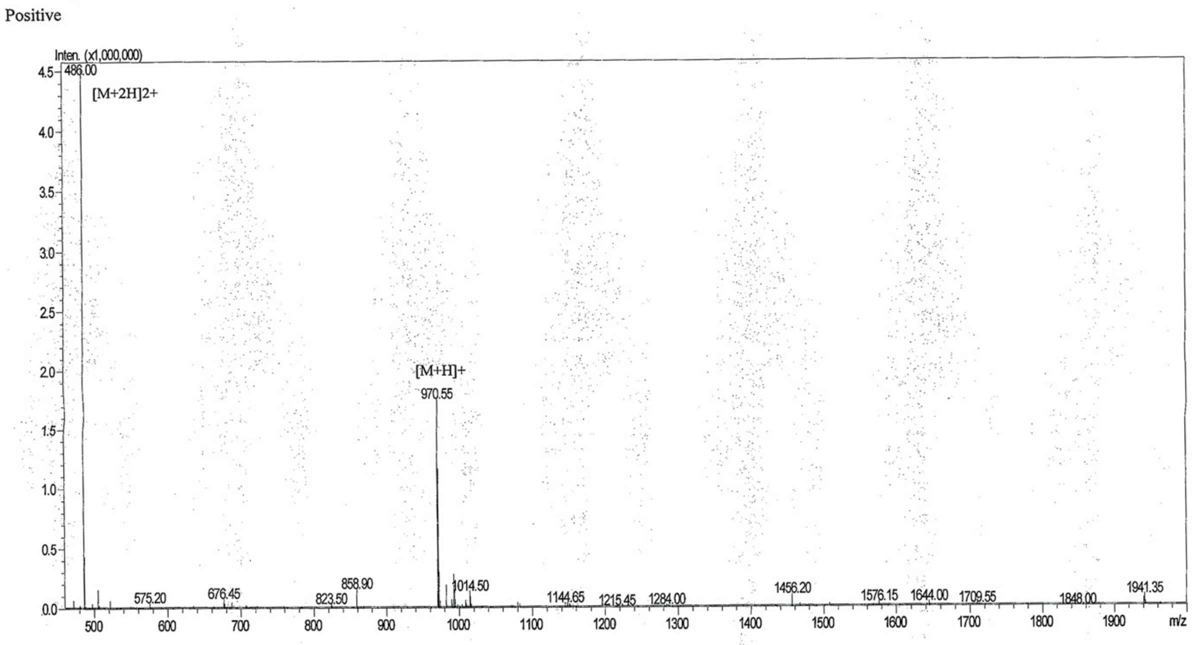

Mw : 970.21
Lot No. : P190510-YS725632

Probe	:ESI	Probe bias	:+4.5kv
Nebulizer Gas Floe	:1.5L/min	Detector	:1.2kv
CDL	:-20.0v	T.Flow	:0.2ml/min
CDL Temp	:250℃	B.conc	:50%H2O/50%ACN
Block Temp	:400℃		

Supplementary Figure 48. LC-MS spectra of FFFIKVAV.

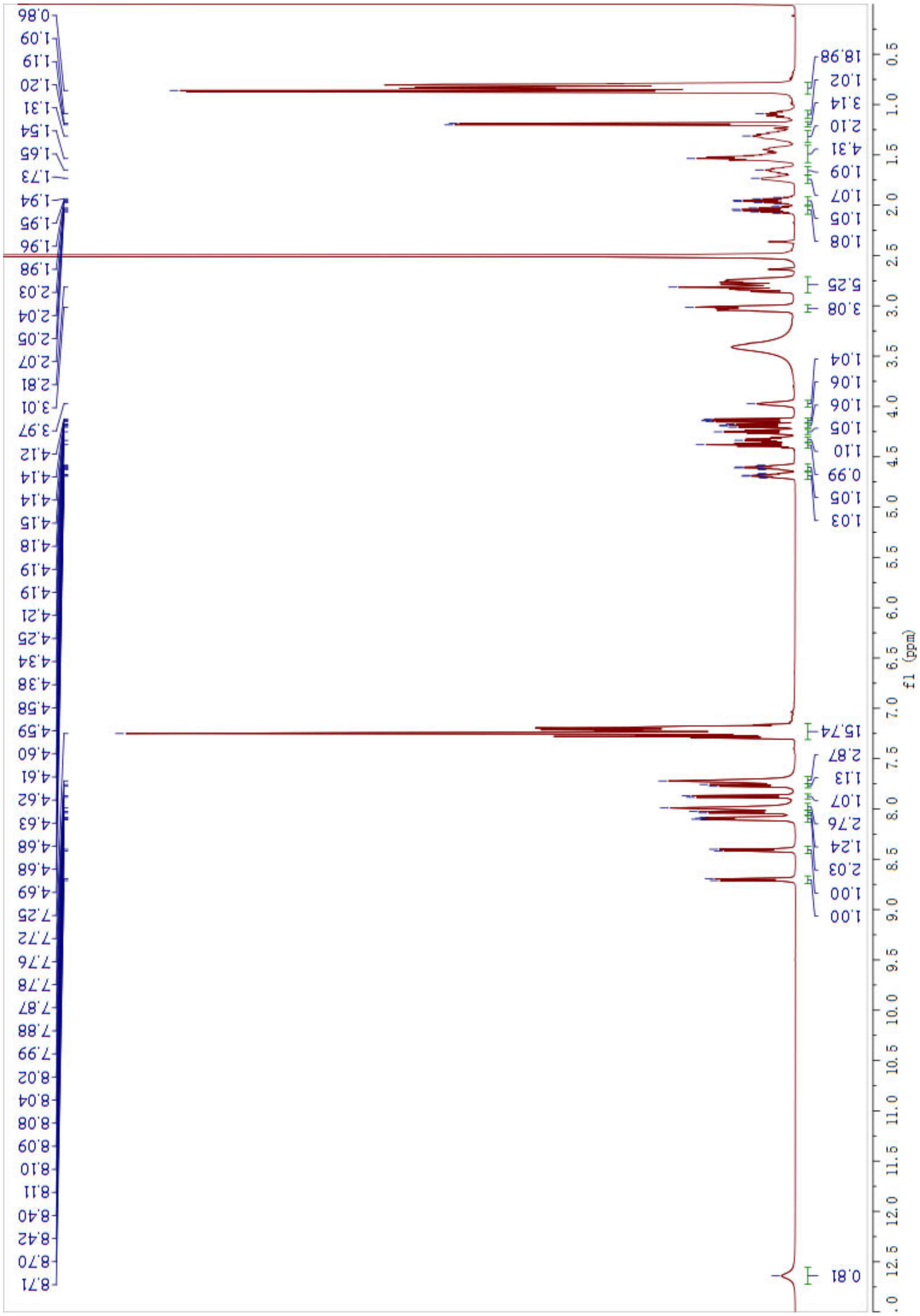

Supplementary Figure 49. ^{1}H NMR of FFFIKVAV
