# Supplementary material for: Control cell migration by engineering integrin ligand assembly
Source: Nat Commun. 2022 Aug 25;13:5002. doi: 10.1038/s41467-022-32686-2 (PMC9411606; doi:10.1038/s41467-022-32686-2)
Supplement: Supplementary file 3 — Reporting Summary [file 41467_2022_32686_MOESM3_ESM.pdf]

nature portfolio

Corresponding author(s): Ye Zhang
Last updated by author(s): 2022/07/27

Reporting Summary

Nature Portfolio wishes to improve the reproducibility of the work that we publish. This form provides structure for consistency and transparency in reporting. For further information on Nature Portfolio policies, see our Editorial Policies and the Editorial Policy Checklist.

Statistics

For all statistical analyses, confirm that the following items are present in the figure legend, table legend, main text, or Methods section.

n/a	Confirmed	
☐	☒	The exact sample size (*n*) for each experimental group/condition, given as a discrete number and unit of measurement
☐	☒	A statement on whether measurements were taken from distinct samples or whether the same sample was measured repeatedly
☐	☒	The statistical test(s) used AND whether they are one- or two-sided<br>*Only common tests should be described solely by name; describe more complex techniques in the Methods section.*
☐	☒	A description of all covariates tested
☐	☒	A description of any assumptions or corrections, such as tests of normality and adjustment for multiple comparisons
☐	☒	A full description of the statistical parameters including central tendency (e.g. means) or other basic estimates (e.g. regression coefficient) AND variation (e.g. standard deviation) or associated estimates of uncertainty (e.g. confidence intervals)
☐	☒	For null hypothesis testing, the test statistic (e.g. *F*, *t*, *r*) with confidence intervals, effect sizes, degrees of freedom and *P* value noted<br>*Give P values as exact values whenever suitable.*
☒	☐	For Bayesian analysis, information on the choice of priors and Markov chain Monte Carlo settings
☒	☐	For hierarchical and complex designs, identification of the appropriate level for tests and full reporting of outcomes
☒	☐	Estimates of effect sizes (e.g. Cohen's *d*, Pearson's *r*), indicating how they were calculated

Our web collection on statistics for biologists contains articles on many of the points above.

Software and code

Policy information about availability of computer code

Data collection	Materials Studio® 2020 for simulation, MO Control version 1.6 for MicroScale Thermophoresis, VICTOR Nivo version 3.0 for microplate reader, FujiFilm LAS-3000 Luminescent Image Analyzer IDX4 for western-blotting, Analytik Jena qPCRsoft version 3.2 for qRT-PCR and Metamorph 7.8.13.0 for confocal imaging, Amnis IDEAS version 6.0 for flow cytometry.
Data analysis	GraphPad Prism version 9.0, imageJ 2.3.0/1.53q, Matlab_R2018a, Zeiss zen lite

For manuscripts utilizing custom algorithms or software that are central to the research but not yet described in published literature, software must be made available to editors and reviewers. We strongly encourage code deposition in a community repository (e.g. GitHub). See the Nature Portfolio guidelines for submitting code & software for further information.

Data

Policy information about availability of data

All manuscripts must include a data availability statement. This statement should provide the following information, where applicable:
- Accession codes, unique identifiers, or web links for publicly available datasets
- A description of any restrictions on data availability
- For clinical datasets or third party data, please ensure that the statement adheres to our policy

Data supporting the findings of this study are available within the article and its Supplementary Information files.

March 2021

Human research participants

Policy information about studies involving human research participants and Sex and Gender in Research.

Reporting on sex and gender	N/A
Population characteristics	N/A
Recruitment	N/A
Ethics oversight	N/A

Note that full information on the approval of the study protocol must also be provided in the manuscript.

Field-specific reporting

Please select the one below that is the best fit for your research. If you are not sure, read the appropriate sections before making your selection.

☒ Life sciences ☐ Behavioural & social sciences ☐ Ecological, evolutionary & environmental sciences

For a reference copy of the document with all sections, see nature.com/documents/nr-reporting-summary-flat.pdf

Life sciences study design

All studies must disclose on these points even when the disclosure is negative.

Sample size	Sample sizes were not statistically pre-determined but were based on the general requirements of assays. For example, more than 160 biological samples were selected for random cell migration assay. We followed the same sample size selections based on the related research and experimental assays that were cited in the main text.
Data exclusions	No data were excluded from the analyses.
Replication	The number of independent experiments for each data panel is indicated in the figure legends and in the source data files. Data shown in the figures represent the aggregate of all independent experiments in most cases. Other data are from a representative experiment (eg. western blots) and in those cases the number of independent experiments that reproduced the finding is also indicated in the figure legends. Few minor experiments were performed once with multiple technical replicates and are indicated in the figure legends.
Randomization	No formal randomization techniques were applied. Samples were allocated randomly to experiments and processed in an arbitrary order.
Blinding	There is no clinical research involved in this study, no blinding was used here.

Reporting for specific materials, systems and methods

We require information from authors about some types of materials, experimental systems and methods used in many studies. Here, indicate whether each material, system or method listed is relevant to your study. If you are not sure if a list item applies to your research, read the appropriate section before selecting a response.

Materials & experimental systems

n/a	Involved in the study	
☐	☒	Antibodies
☐	☒	Eukaryotic cell lines
☒	☐	Palaeontology and archaeology
☒	☐	Animals and other organisms
☒	☐	Clinical data
☒	☐	Dual use research of concern

Methods

n/a	Involved in the study	
☒	☐	ChIP-seq
☐	☒	Flow cytometry
☒	☐	MRI-based neuroimaging

Antibodies

Antibodies used	The following antibodies have been applied in this study:<br>anti- integrin beta 1 (12G10, ab30394, Abcam, 1:100 for WB and 1:200 for ICC and Flow Cyt),

anti-paxillin (Y113, ab32084, Abcam, 1:200, ICC),
anti-GAPDH (6C5, ab8245, Abcam, 1:1000, WB)
anti-integrin alpha 6 (EPR18124,ab191551, Abcam, 1:200, WB),
anti-CD49c (integrin alpha 3, ASC-1, #MA5-28565, Invitrogen, 1:50 for ICC, 1:100 for WB and Flow Cyt),
anti-talin 1 (8D4, ab157808, Abcam, 1:100, ICC),
anti-vinculin (EPR8185, ab129002, Abcam, 1:100, ICC),
anti-FAK (#3285, Cell signaling Technology, 1:200, ICC),
anti-α-actinin (H-2, sc-17829, Santa Cruz Biotechnology, 1:200, ICC),
anti-Phospho-Myosin Light Chain 2 (Thr18/Ser19) (pMLC, #3674, Cell Signaling Technology, 1:200 for WB and 1:100 for ICC)
anti-laminin-5 (P3H9-2, MAB1947, Chemicon, 5 μg/ml for functional blocking),
anti-fibronectin (IST-9, ab6328, Abcam, 20 μg/ml for functional blocking)
Mouse IgG-Isotype Control (ab37355, Abcam, 1:100 for Flow Cyto)
Goat Anti-Mouse IgG H&L (Alexa Fluor® 488) (ab150113, Abcam, 1:1000 for Flow Cyt and ICC)
Goat Anti-Rabbit IgG H&L (Alexa Fluor® 488) (ab150077, Abcam, 1:1000 for ICC)
Goat Anti-Rabbit IgG H&L (Alexa Fluor® 568) (ab175471, Abcam, 1:1000 for ICC)
Donkey Anti-Rabbit IgG H&L (Alexa Fluor® 647) (ab150075, Abcam, 1:1000 for ICC)
Goat Anti-Mouse IgG H&L (Alexa Fluor® 568) (ab175473, Abcam, 1:1000 for ICC)
Goat Anti-Mouse IgG H&L (Alexa Fluor® 647) (ab150115, Abcam, 1:1000 for ICC)
Goat anti-Mouse IgG (H+L) Cross-Adsorbed Secondary Antibody, HRP (G-21040, Invitrogen, 1:1000 for WB)
Goat anti-Rabbit IgG (H+L) Secondary Antibody, HRP (#31460, Invitrogen, 1:1000 for WB)

Validation

All antibodies are commercially available and have been validated by the manufacturers:

anti- integrin beta 1 (12G10, ab30394, Abcam, https://www.abcam.com/integrin-beta-1-antibody-12g10-ab30394.html):
According to the manufacture, this monoclonal antibody to integrin beta 1 has been knockout validated in ICC/IF and flow cytometry. The expected signal was observed in wild type cells and was not seen in knockout cells.

anti-paxillin (Y113, ab32084,Abcam, https://www.abcam.com/paxillin-antibody-y113-ab32084.html):
According to the manufacture, this recombinant Rabbit monoclonal antibody to paxillin has been knockout validated.

anti-GAPDH (6C5, ab8245, Abcam, https://www.abcam.com/gapdh-antibody-6c5-loading-control-ab8245.html):
According to the manufacture, this GAPDH antibody can be used as a loading control antibody. There is no cross-reaction with GAPDH from yeast. Preliminary data indicates that the GAPDH antibody- loading control ab8245 recognizes the monomer (36 kDa) and also the dimer forms of GAPDH, but not the tetrameric form of the protein.

anti-integrin alpha 6 (EPR18124, ab181551, Abcam, https://www.abcam.com/integrin-alpha-6-antibody-epr18124-ab181551.html):
According to the manufacture, this rabbit monoclonal antibody to integrin alpha 6 has been knockout validated.

anti-CD49c (integrin alpha 3, ASC-1, MA5-28565, Invitrogen, https://www.thermofisher.cn/cn/zh/antibody/product/CD49c-Integrin-alpha-3-Antibody-clone-ASC-1-Monoclonal/MA5-28565?adobe_mc=MCMID%7C76839238593868506262246185686987625884%7CMCAID%3D311CCFECD388F28C-400013B67BF04FC3%7CMCORGID%3D5B135A0C5370E6B40A490D44%40AdobeOrg%7CTS=1614293705):
According to the manufacture, this antibody has been verified by relative expression to ensure that the antibody binds to the antigen stated.

anti-talin 1 (8D4, ab157808, Abcam, https://www.abcam.com/talin-1-antibody-8d4-ab157808.html):
According to the manufacture, this antibody recognizes an epitope within the rod domain of Talin 1.

anti-vinculin (EPR8185, ab129002, Abcam, https://www.abcam.com/vinculin-antibody-epr8185-ab129002.html):
According to the manufacture, their Abpromise guarantee covers the use of ab129002 in the application of WB, IP, and ICC/IF.

anti-FAK (#3285, Cell signaling Technology, https://www.cellsignal.com/products/primary-antibodies/fak-antibody/3285):
According to the manufacture, this antibody has been Knockout validated in WB and IF.

anti-α-actinin (H-2, sc-17829, Santa Cruz Biotechnology, https://www.scbt.com/p/alpha-actinin-antibody-h-2?productCanUrl=alpha-actinin-antibody-h-2&_requestid=8426724):
According to the manufacture, this antibody has been validated by relative expression in WB and ICC/IF.

anti-Phospho-Myosin Light Chain 2 (Thr18/Ser19) (pMLC, #3674, Cell Signaling Technology, https://www.cellsignal.com/products/primary-antibodies/phospho-myosin-light-chain-2-thr18-ser19-antibody/3674):
According to the manufacture, this antibody has been validated by relative expression in WB.

anti-laminin-5 (P3H9-2, MAB1947, Chemicon, https://www.sigmaaldrich.com/US/en/product/mm/mab1947):
According to the manufacture, Detect Laminin-5 using this Anti-Laminin-5 Antibody, clone P3H9-2 validated for use in ELISA, IP, WB, IC, IH and it is suitable for the application of inhibiting LM-5 binding.

anti-fibronectin (IST-9, ab6328, Abcam, https://www.abcam.com/fibronectin-antibody-ist-9-bsa-and-azide-free-ab6328.html):
According to the manufacture website, this antibody has been validated in inhibition of FN binding.

Eukaryotic cell lines

Policy information about cell lines and Sex and Gender in Research

Cell line source(s)	Human hepatocellular carcinoma cell lines HuH-7 and Hep G2, human gastric adenocarcinoma cell line MKN1, human breast cancer cell line MCF-7 and human cervical cancer cell line HeLa were purchased from Riken BioResource Research Center. Human lung carcinoma cell line A549, human glioblastoma astrocytoma cell line U-87 MG, and human ectocervical cell line Ect1/E6E7 were purchased from American Type Culture Collection (ATCC).
Authentication	none of the cell lines were further authenticated in our lab.
Mycoplasma contamination	All of the cell lines were tested negative for mycoplasma.
Commonly misidentified lines (See ICLAC register)	No commonly misidentified lines were used

Flow Cytometry

Plots

Confirm that:

☒ The axis labels state the marker and fluorochrome used (e.g. CD4-FITC).

☒ The axis scales are clearly visible. Include numbers along axes only for bottom left plot of group (a 'group' is an analysis of identical markers).

☒ All plots are contour plots with outliers or pseudocolor plots.

☒ A numerical value for number of cells or percentage (with statistics) is provided.

Methodology

Sample preparation	Cells were resuspended in culture medium and filtered through a 40 µm cell strainer.
Instrument	ImageStream X Mark II, Merck
Software	amnis IDEAS (Version 6.0)
Cell population abundance	The abundance of the relevant cell populations within post-sort fractions was 90-98% in experiments.
Gating strategy	Cells were isolated in a single-cell manner for positive FITC/GFP signals.

☒ Tick this box to confirm that a figure exemplifying the gating strategy is provided in the Supplementary Information.
